# LncRNA LYPLAL1-DT screening from type 2 diabetes with macrovascular complication contributes protective effects on human umbilical vein endothelial cells via regulating the miR-204-5p/SIRT1 axis

**DOI:** 10.1038/s41420-022-01019-z

**Published:** 2022-05-04

**Authors:** Xiao Zhu, Yihan Liu, Jia Cui, Jianyi Lv, Changlong Li, Jing Lu, Xueyun Huo, Jingtao Dou, Zhigang Bai, Zhenwen Chen, Xiaoyan Du

**Affiliations:** 1 Department of medical genetics and biological development, School of Basic Medical Sciences, Capital Medical University, No.10 Xitoutiao, Youanmen, Fengtai District, Beijing, 100069 China; 2 Institute of Acupuncture and Moxibustion, China Academy of Chinese Medical Sciences, Beijing, 100700 China; 3 Department of Endocrinology, Chinese PLA General Hospital, Beijing, 100853 China; 4 Department of General Surgery, Beijing Friendship Hospital, Capital Medical University, Beijing Key Laboratory of Cancer Invasion and Metastasis Research & National Clinical Research Center for Digestive Diseases, Beijing, China

**Keywords:** Apoptosis, Long non-coding RNAs

## Abstract

Long noncoding RNAs (lncRNAs) are involved in diabetes related diseases. However, the role of lncRNAs in the pathogenesis of type 2 diabetes with macrovascular complication (DMC) has seldomly been recognized. This study screened lncRNA profiles of leukocytes from DMC patients and explored protective role of lncRNA LYPLAL1-DT in endothelial cells (EC) under high glucose (HG) and inflammatory conditions (IS). Between DMC and healthy controls, 477 differential expression lncRNAs (DE-lncRNAs) were identified. The enrichment and pathway analysis showed that most of the DE-lncRNAs belonged to inflammatory, metabolic, and vascular diseases. A total of 12 lncRNAs was validated as significant DE-lncRNAs in expanding cohorts. Furthermore, these DE-lncRNAs were shown to be significantly related to hypoxia, HG, and IS in EC, especially lncRNA LYPLAL1-DT. LYPLAL1-DT overexpression results in the promotion of the proliferation, and migration of EC, as well as an elevation of autophagy. Overexpressed LYPLAL1-DT reduces the adhesion of monocytes to EC, boosts anti-inflammation, and suppresses inflammatory molecules secreted in the medium. Mechanistically, LYPLAL1-DT acts as competing endogenous RNA (ceRNA) by downregulating miR-204-5p, therefore enhancing SIRT1 and protecting EC autophagy function; thus, alleviating apoptosis. Finally, exosome sequencing revealed LYPLAL1-DT expression was 4 times lower in DMC cells than in healthy samples. In general, we identified LYPLAL1-DT having protective effects on EC as ceRNA mediated through the miR-204-5p/SIRT1 pathway. Therefore, it inhibits the autophagy of EC as well as modulating systemic inflammation. This approach could be regarded as a new potential therapeutic target in DMC.

## Introduction

Over the past three decades, the prevalence of diabetes has sharply increased worldwide. It is a major cause of blindness, kidney failure, heart attacks, stroke, and lower limb amputation [1]. Diabetes is usually recognized as a vascular disease characterized by vasoregulatory changes, inflammatory activation resulting from high glucose (HG), and hypoxia (HO). Diabetic vascular complications include micro and macrovascular dysfunctions occurring in several organs, such as the muscles, eyes, skin, kidneys, brain, and heart [2]. More than 50% of diabetic patients die of diabetic macrovascular complications (DMC) such as cardiovascular disease, making it a major cause of morbidity and mortality. The leading cause of DMC is hyperglycemia induced endothelial injury or dysfunction, and inflammation, therefore resulting in atherosclerosis [3]. Hence, understanding the molecular mechanisms causing endothelial injury under hyper-glucose and inflammatory states would provide a fundamental understanding in the rational design of a DMC therapeutic agent.

Long non-coding RNAs (lncRNAs) are recognized as a class of non-coding single-stranded RNAs longer than 200 bases with no evident function of coding proteins [4]. Most lncRNAs are located in the nucleus, however can also be found in the cytoplasm where they function as molecular scaffolds, motifs in alternative splicing, or in the modification of chromatin structures [5]. Growing evidence has proved that dysregulated or dysfunctional lncRNAs have emerging roles in more than 200 diseases, and new associations continue to accumulate within diabetic literature [6]. LncRNAs operate through many molecular mechanisms, as an example lncRNA MALAT1 either acts as a competing endogenous RNA (ceRNA) for miR-205-5p in human mesenchymal stem cells regulating VEGF production improving endothelial cell tubule formation [7], or upregulate the inflammatory mediators IL-6 and TNF-a through activation of serum amyloid antigen 3 [8]. Studies investigating the link between lncRNAs and the development of diabetic complications have just begun [4]. Advances in sequencing and microarray technology enable the identification of large numbers of putative lncRNA loci and accelerate the progress within this field of research [9, 10]. Although several studies have focused on the correlation of lncRNA and vascular diseases [11–13], the role and characteristics of vascular abnormalities in diabetes caused by lncRNAs involved in hyperglucose and inflammation remain poorly understood [11, 14–17]. In the present study, we screened differentially expressed lncRNAs in circulating leukocytes isolated from diabetic macrovascular complicated individuals using RNA sequencing. We identified new lncRNAs related to DMC abnormal endothelial cells and further explored the protective effects of lncRNA LYPLAL1-DT as a ceRNA to inhibit miR-204-5p and upregulate SIRT1 in order to promote autophagy of endothelial cells (EC), as well as ameliorate inflammation of EC. These results reveal a novel lncRNA as a potential diagnostic marker and therapeutic target for DMC treatment in clinical practice.

## Results

### Transcriptomic profiles in patients with diabetic macrovascular complications

**Fig. 1:**
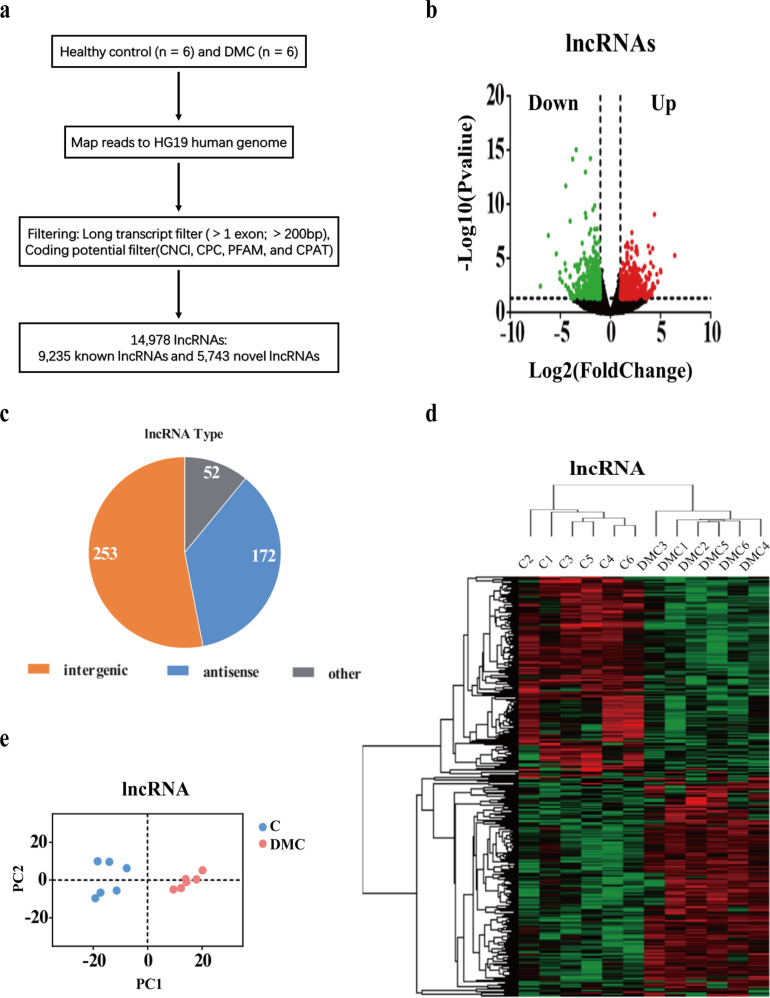
Characteristics of lncRNAs transcription in type 2 diabetes macrovascular complication. **a** A schematic illustration of the procedure to identify and define lncRNAs in the leukocytes of DMC patients. **b** Differentially expressed lncRNAs were identified from a Volcano plot showing data from DMC diabetes patient (*n* = 6) relative to healthy controls (*n* = 6). The vertical black lines correspond to 2-fold up and downregulations, respectively; and the horizontal black line represents a *p* value of 0.05. The red and green points in the plots represent the differentially expressed genes with statistical significance of the upregulation and downregulation of lncRNA, respectively. **c** Pie chart representations shows the proportion of DMC lncRNAs that are transcribed as antisense (blue), intergenic (orange), or other types (grey). **d** Differential lncRNA expression profiles were the hierarchical cluster was analyzed and shown as a heatmap. **e** Principal component analysis shows similar results also presented as a heatmap.

The transcriptome sequencing in the discovery cohorts composed of 6 patients diagnosed with DMC and 6 health controls was used to determine the transcriptomic profiles. The data process flow chart is shown in Fig. 1a. The information of the DMC participants in both the discovery and the expanded cohorts are shown in Table 1, and demonstrates that most of the clinical characteristics were not significantly different between the two cohorts. Thus, the lncRNAs selected in the discovery cohort are reliably validated in the expanded cohort.

**Table 1:** Clinical characteristics of included patients of type 2 diabetes with macrovascular complication (DMC) in discovery cohorts (*n* = 6) and in validation cohorts (*n* = 46).

Items	DMC in discovery cohorts (*n* = 6)	DMC in validation cohorts (*n* = 46)
Age (years)	55.50 ± 5.82	56.70 ± 7.89
Sex (male%)	83.33%	81.43%
BMI (kg/m2)	23.92 ± 1.24	25.66 ± 3.13
Fasting glucose (mmo/L)	7.51 ± 3.05	8.36 ± 3.64
HbA1c	8.45 ± 1.72	8.50 ± 1.93
Total cholesterol (mg/dL)	5.10 ± 0.90	10.68 ± 51.76
Fasting triglycerides (mg/dL)	1.96 ± 0.99	2.14 ± 2.20
HDL (mg/dL)	1.14 ± 0.39	4.65 ± 29.46
LDL (mg/dL)	3.26 ± 0.70	2.55 ± 0.87

First, we described the transcriptomic profiles of DMC to identify the critical genes and lncRNAs in the DMC patients. A total of 14,978 lncRNAs and 17,172 mRNAs were detected in leukocytes from the DMC patients. Of these, 9235 lncRNAs have been registered in databases and defined as known. Out of the 9235 lncRNA’s, 4647 were upregulated, and 4588 were downregulated. The other 5743 lncRNAs were identified for the first time and were defined as either upregulated (3379) or downregulated (2364) novel lncRNAs. After optimization using an adjusted *p* value (threshold of <0.05) and fold change >2, we identified 477 significantly differentially expressed (DE) lncRNAs (DMC-lncRNAs) (Table S3), among which 245 were downregulated and 232 were upregulated (Fig. 1b). The top 20 DMC-lncRNAs are shown in Table 2. We further analyzed the different biotypes of the 477 DE-lncRNAs. The results indicated that 172 lncRNAs (36.1%) were antisense, 253 (53.0%) were intergenic (lincRNA), and 52 (10.9%) belonged to other types (sense-overlapping or sense-intronic) (Fig. 1c, Table S3). Furthermore, 798 DMC-mRNAs were found by similar optimization (Table S4), with 491 downregulated and 307 upregulated (Fig. S1a). Both DMC-lncRNA and DMC-mRNA were distinguishable within DMC patients and the healthy controls by hierarchical clustering (Figs. 1d, S1b) and principal content analysis (Figs. 1e, S1c). This data clearly illustrates a distinguishable differential expression profile of the leukocytes between DMC patients and the healthy controls.

**Table 2:** The top 20 lncRNA with significantly differential expression in white blood cell from diabetes with macrovascular complication (DMC) and health control (C).

Gene	C normalize	DMC normalize	Log2 FC	FDR	Up/Down	Biotype
ENSG00000273338	109.992	10.1978	−3.43107	3.52E-12	down	antisense
MSTRG.74858	82.0596	5.99718	−3.77432	8.23E-12	down	linc
MSTRG.179846	1146.59	289.948	−1.98349	1.10E-11	down	antisense
ENSG00000270069	441.771	78.9949	−2.48347	1.03E-10	down	linc
MSTRG.159146	41.0073	1.86011	−4.46242	1.48E-09	down	antisense
MSTRG.131944	2338.92	790.908	−1.56426	8.50E-08	down	antisense
MSTRG.103146	630.434	189.15	−1.73681	1.60E-07	down	antisense
MSTRG.80841	1.10689	23.3297	4.39759	3.55E-07	up	linc
MSTRG.182419	92.9662	17.2915	−2.42665	6.15E-07	down	linc
MSTRG.159131	27.583	1.69268	−4.0264	1.23E-06	down	linc
MSTRG.187859	186.565	48.1136	−1.95516	5.75E-06	down	linc
MSTRG.106807	3095.66	1060.73	−1.54518	5.76E-06	down	antisense
ENSG00000251022	1004.1	401.354	−1.32295	6.08E-06	down	antisense
MSTRG.111791	29.3953	129.086	2.13468	1.02E-05	up	linc
MSTRG.95088	12.1535	0.16631	−6.19132	1.70E-05	down	linc
MSTRG.116382	324.357	107.277	−1.59624	2.31E-05	down	linc
ENSG00000224307	38.2435	171.609	2.16584	5.80E-05	up	linc
MSTRG.30000	75.0362	242.855	1.69444	6.61E-05	up	linc
ENSG00000269902	88.1519	12.6132	−2.80506	6.66E-05	down	linc
MSTRG.183281	353.29	137.464	−1.3618	6.86E-05	down	antisense

### Features of DE-lncRNAs and mRNAs identified in patients with DMC

**Fig. 2:**
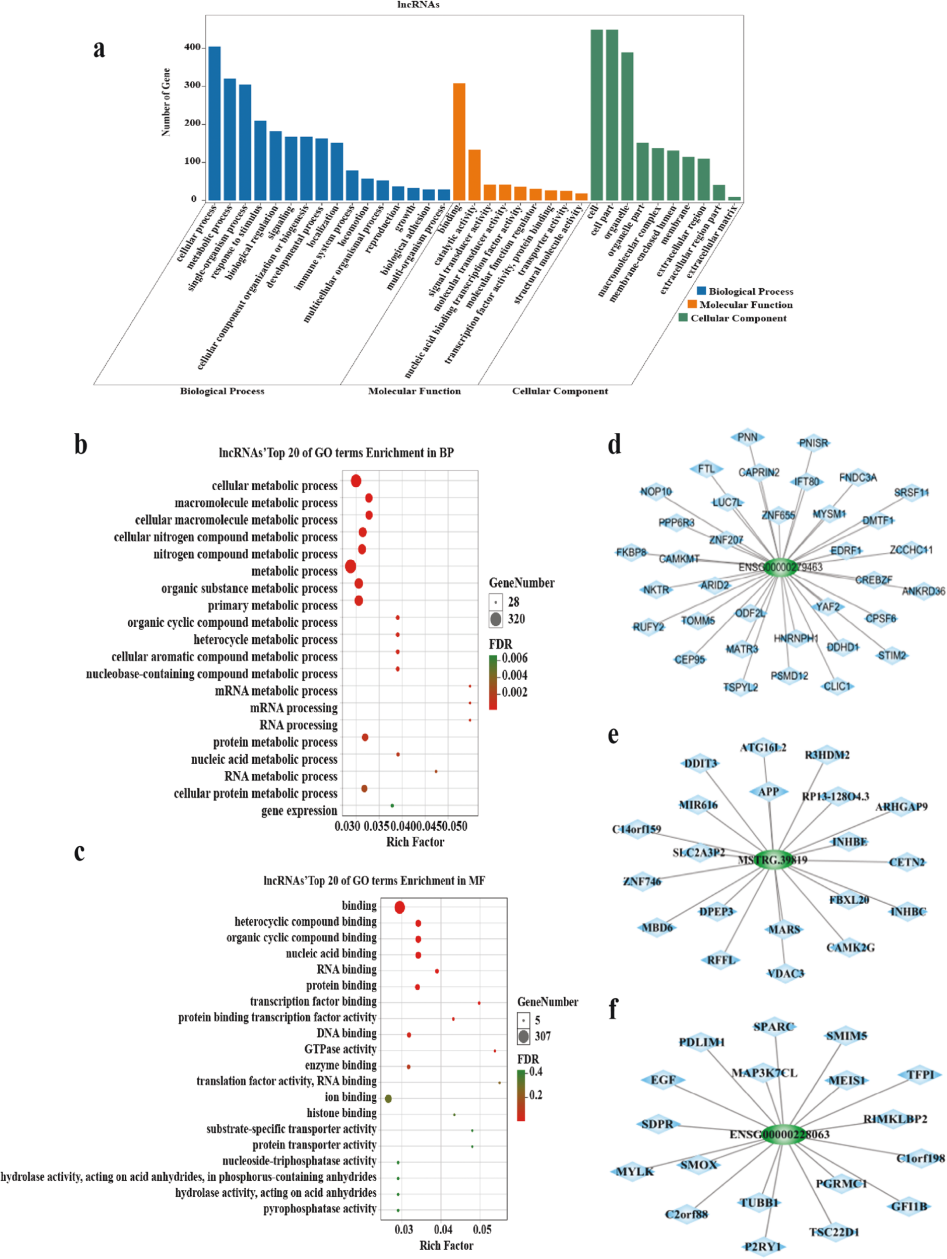
Bioinformatic analysis via enrichment analysis of GO terms for DMC-lncRNAs and co-expression networks for DMC-lncRNAs and mRNAs. **a** GO analysis of DMC-lncRNAs within biological processes, molecular functions, and cellular components. **b** The top 20 GO terms of lncRNAs enriched in biological processes. **c** The top 20 GO terms of lncRNAs enriched in molecular function. **d**–**f** The co-expression network of 3 lncRNAs including ENSG00000279463, MSTRG.39819 and ENSG00000228063.

The Gene Ontology (GO) enrichment analysis was applied to classify DMC patients’ DE-lncRNAs and DE-mRNAs. Under the biological process category, metabolic process, single organism process, response to stimulus, and cellular process were the top 4 DE-lncRNAs; similar items were found for the DE-mRNAs (Figs. 2a, S2). Under the molecular function category, both DE-lncRNAs and DE-mRNAs showed the highest percentages in catalytic activity and signal transducer activity items (Figs. 2a, S2). The top 20 items for DE-lncRNA demonstrated that most biological processes involved metabolic processes (17/20, 85%) (Fig. 2b). The highest molecular function is binding including RNA, DNA, and protein binding (Fig. 2c). These results imply that DE-lncRNAs identified from the DMC patients are involved in transcription regulation.

We structured co-expression networks to determine if lncRNAs are associated with lncRNAs or mRNAs. There were 509 genes involved in our co-expression networks consisting of 117 lncRNAs and 392 mRNAs (Fig. S3), with 7 lncRNAs and 18 mRNAs harboring more than 10 related genes in the CNC network. The top 10 lncRNAs and mRNAs with the number of genes to which they are related are shown in Table 3. In this CNC network, lncRNAs ENSG00000279463, MSTRG.39819, and ENSG00000228063 were the top 3 lncRNAs with the greatest number of related genes (35, 20, and 18) (Fig. 2d–f). The related genes such as TOMM5, and MYLK have been reported to have implications in diabetes [18], and PDLIM1 and CAMK2G were found to be related to atherosclerosis [19]. We also analyzed the characteristics of 9059 transcripts of novel lncRNAs identified in DMC patients. Most novel lncRNA transcripts harbored 2 exons (7060, 77.93%) (Fig. S4a). The lengths of most of the novel lncRNAs (71.91%) were less than 2000 bp (Fig. S4b). The conservation analysis in humans indicated that more than half (61.3%) had conservation scores (CS) less than 0.1 and 6.77% less than 0.01 between humans and other species (Fig. S4c). The distribution of chromosomal transcripts demonstrated that novel lncRNAs were primarily distributed on chr1 to chr6, and that most lncRNAs with low CS in humans were from the same chromosome (Fig. S5). These results reflect that lncRNAs in leukocytes may have an important effect on DMC.

**Table 3:** The top 10 lncRNAs and mRNAs with numbers of related genes in co-expression network.

Gene	Biotype	Target gene counts
ENSG00000279463	lncRNA	35
POLR2K	mRNA	24
APP	mRNA	21
RAC1	mRNA	20
MSTRG.39819	lncRNA	17
ENSG00000228063	lncRNA	17
RHOA	mRNA	17
GNGT2	mRNA	16
CDC42	mRNA	15
MAPK3	mRNA	15

### Validation measurement of DE-lncRNAs in expanding groups

**Fig. 3:**
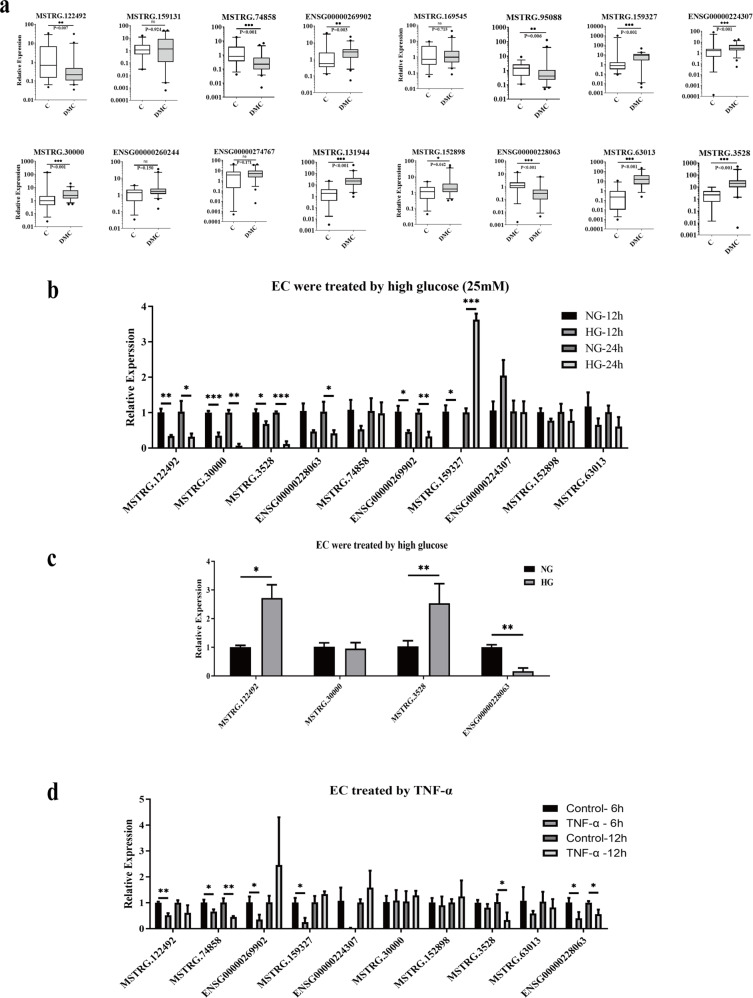
Validation of lncRNAs in DMC patients and lncRNAs expression levels induced by high glucose and TNF-α in HUVEC (DMC *n* = 46, healthy control *n* = 36). **a** 12 of the 16 lncRNAs showed significant difference in expression levels between the DMC patients (*n* = 46) and the healthy controls (*n* = 36) analyzed by real-time PCR. **b** The lncRNAs expression in HUVEC after treated with 25 mM glucose for 12 h and 24 h. **c** The lncRNAs expression in HUVEC after treatment with 25 mM glucose and then resuming normal culture for 72 h. **d** The lncRNAs expression in HUVEC after being 3induced by TNF-α for 12 h and 24 h. “NS”: no significant difference; “*” indicated significant difference with *p* < 0.05, “**” indicated *p* < 0.01 and “***” indicated *p* < 0.001.

To confirm the DE-lncRNAs, we independently detected 16 lncRNAs in the validation group of DMC patients (*n* = 46) and healthy controls (*n* = 36) via real-time PCR. The expression levels of these lncRNAs in the validation cohort are shown in Fig. 3a. Of the 16 tested lncRNAs, 12 lncRNAs (75%) displayed dramatically different expression levels between the DMC and the healthy control groups; 4 lncRNAs were downregulated, and the others were upregulated in DMC. Nine novel lncRNAs and 3 known lncRNAs were positively confirmed. In addition, 8 lncRNAs belonged to the lincRNA, whereas 4 lncRNAs belonged to the antisense group (Table 4). Comparing the validation results and sequencing data revealed that most lncRNAs (75%, 12/16) displayed similar trends, with 10 lncRNAs exhibiting the same significantly positive results as observed in the sequencing data (Fig. S6). Among the 12 significantly different expression levels of lncRNAs, 8 lncRNAs harbor predicted target genes (Table S5).

**Table 4:** The results of analysis by the categorical features of 16 lncRNAs which were chosen in validation cohorts. “*” marked the validation positive lncRNA.

Categorical features	LncRNA	Ratio of lncRNA with significant difference
Known lncRNA	ENSG00000269902*, ENSG00000224307*, ENSG00000228063*, ENSG00000260244, ENSG00000274767	3/5 (60.00%)
Novel lncRNA	MSTRG.122492*, MSTRG.74858*, MSTRG.95088*. MSTRG.159327*, MSTRG.30000*, MSTRG.131944*, MSTRG.152898*, MSTRG.63013*, MSTRG.3528*, MSTRG.159131, MSTRG.169545	9/11 (81.82%)
LincRNA	ENSG00000269902*, ENSG00000224307*, MSTRG.122492*, MSTRG.74858*, MSTRG.95088*, MSTRG.30000*, MSTRG.63013*, MSTRG.3528*, MSTRG.159131, MSTRG.169545	8/10 (80.00%)
Antisense lncRNA	ENSG00000228063*, ENSG00000274767, MSTRG.159327*, MSTRG.131944*, MSTRG.152898*	4/5 (80.00%)

We sought to identify potential orthologs of our selected lncRNAs by comparing their sequences with previously identified murine lncRNAs. Of the 12 validation-positive lncRNA, 8 (66.67%) exhibited orthologous sequences in the mouse genome (Table 5). Thus, these validated lncRNAs exhibited comparable conservation in humans and mice, which could lead to the use of a mouse model to directly investigate lncRNA biomarkers.

**Table 5:** The information of 12 lncRNAs positively validated in the expanding cohort in present study and their potential orthologous sequences comparing with mouse data.

LncRNA	Novel/known	The log2 value in the RNA-seq data	Orthologous sequence with mouse
ENSG00000269902	known	−2.81	Not found
ENSG00000224307	known	2.17	Not found
ENSG00000228063	known	−1.22	Not found
MSTRG.122492	novel	−3.65	chr3:58,478,307-58,478,532
MSTRG.74858	novel	−3.77	chr11:121,806,938-121,808,036
MSTRG.95088	novel	−6.19	chr2:45,237,395-45,239,069
MSTRG.159327	novel	1.81	chr5:143,473,978-143,503,553
MSTRG.30000	novel	1.69	chr2:91,023,089-91,023,445
MSTRG.131944	novel	−1.56	chr3:136,106,245-136,114,883
MSTRG.152898	novel	2.91	Not found
MSTRG.63013	novel	1.73	chr17:23,644,824-23,660,240
MSTRG.3528	novel	2.64	chr4:118,424,930-118,425,109

### DE-lncRNAs response to HG and IS conditions in EC

The pathological changes of DMC are primarily due to higher serum glucose levels inducing abnormal metabolism, consequently influencing the functions and homeostasis of ECs [20–22]. A better understanding of lncRNAs response in EC under HG and IS is essential. Therefore, we tested the expression of 10 lncRNAs which are positively validated and exhibited the same significant trend as observed in the sequencing data within the HUVEC cell line. As shown in Figs. 3b, S7a, 3 lncRNAs exhibited increased levels after a 12 h and 24 h treatment with 25 nM, and 30 nM of glucose, respectively. LncRNA ENSG00000228063 was significantly downregulated after a 24 h treatment with HG in ECs (Figs. 3b, S7a).

Based on the “metabolic memory,” which is defined as a phenomenon that the vasculature can remember transient hyperglycemia for quite an extended period even after the reestablishment of normoglycemia [23], we tested if there was a metabolic memory of these 4 lncRNAs in ECs. The results illustrated that the lncRNA MSTRG.122492 and MSTRG.3528 were significantly upregulated, and ENSG000000228063 was dramatically downregulated (Fig. 3c). We also performed HO and IS treatment on ECs to mimic the environment of the pathological changes in DMC. The results showed that the expression of lncRNA MSTRG.3528 was significantly higher after a 12 h HO stimulus, the others exhibited no change (Fig. S7b). The expression of lncRNA MSTRG.74858 and ENSG00000228063 markedly decreased after a 6 h TNF-α treatment. MSTRG.3528 was downregulated after a 12 h treatment with TNF-α. MSTRG.122492, ENSG00000269902, and MSTRG.159327 expression was downregulated after 6 h and then returned to normal levels after 12 h (Fig. 3d). All the above results imply that ENSG00000228063 is the most sensitive in response to HG, HO, and IS.

### LncRNA LYPLAL1-DT alleviates the influence of HG and IS on the proliferation, migration, autophagy, apoptosis, and inflammatory response of ECs

**Fig. 4:**
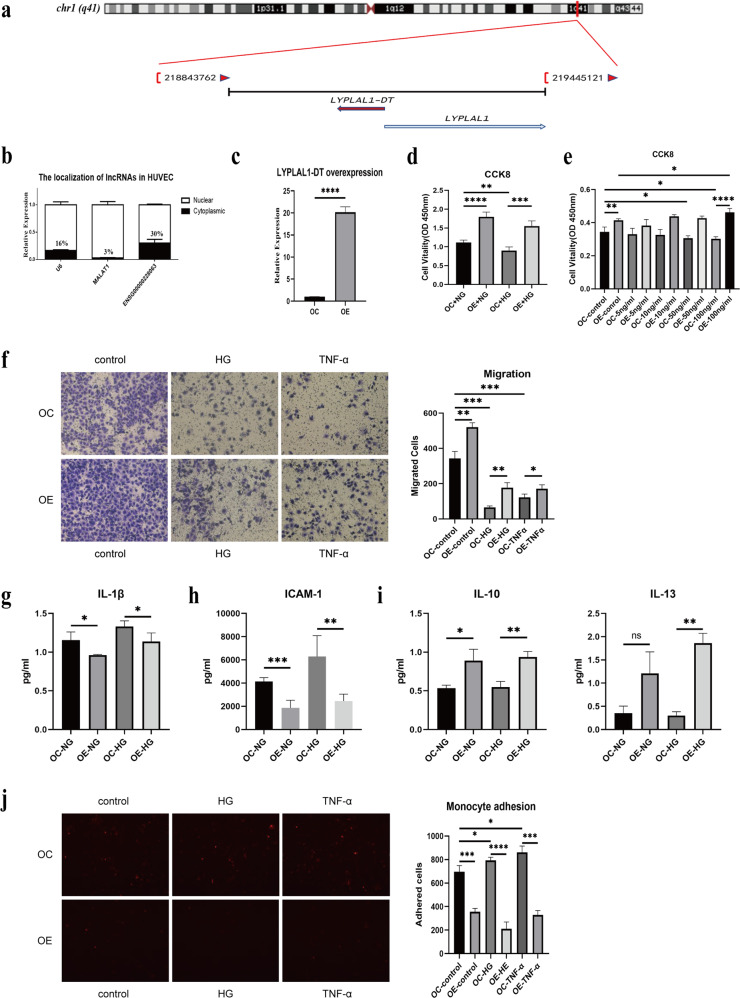
The location of LYPLAL1-DT and the biological function of LYPLAL1-DT in HUVEC. **a** The location of LYPLAL1-DT on the chromosome. **b** The Subcellular localization of LYPLAL1-DT in HUVEC. **c** Establishment of LYPLAL1-DT-overexpression cell line with a pLenti-GIII-CMV-CBH-GFP-2A-Puro vector being transfected into HUVEC was identified by real-time PCR. **d**, **e** CCK-8 assays were performed to determine the viability of LYPLAL1-DT-overexpressing cells treated with HG (25 mM) or induced by TNF-α for 12 h. **f** Transwell assays were used to investigate the migration of LYPLAL1-DT-overexpressing cells treated with HG (25 mM) or induced by TNF-α (100 ng/ml) for 12 h. **g**, **h**, **i** The HG-treated and TNF-α-induced cell culture medium were collected to detect the expression levels of cytokines, including IL-1β, ICAM-1, IL-10, and IL-13, using ELISA Kits. **j** LYPLAL1-DT-overexpression HUVECs were pretreated with HG or TNF-α as described above, then adhered Dil-stained THP-1 cells were visualized by fluorescence microscopy. “*” indicated significant difference with *p* < 0.05, “**” indicated *p* < 0.01 and “***” indicated *p* < 0.001.

Based on the HG/IS test results lncRNA ENSG00000228063 exhibited metabolic memory; we chose this transcript for further investigation. ENSG00000228063 is located on 1q41 LLP1 and is within 1 kb of LYPLAL1. Therefore, it was named LYPLAL1-DT (Ensembl ID: ENSG00000228063) (Fig. 4a). The major variant is 2577 nt in length and encoded by five exons. LYPLAL1-DT is highly conserved in humans, with homologous sequences found only in chimpanzees, and other nonhuman primate animals (Fig. S7c), having a very low homology in mice. LYPLAL1-DT is expressed across diverse human tissues, including vascular tissue and ECs. As the position of the lncRNA is closely associated with its function, we first explored the location of LYPLAL1-DT in HUVEC cells. As shown in Fig. 4b, LYPLAL1-DT is expressed in both the nucleus and the cytoplasm with primary localization in the cytoplasm, indicating that LYPLAL1-DT could exert cis-regulation or play a role as a competitive endogenous RNA (ceRNA).

To investigate the function of LYPLAL1-DT in ECs, we successfully constructed overexpressing HUVECs (OE) and their corresponding control cell line (OC) (Fig. 4C). The CCK8 staining results showed that LYPLAL1-DT significantly promotes the vitality of EC, especially under HG conditions (Fig. 4d). LYPLAL1-DT increases the proliferation of EC under an IS mimic via treatment with TNF-a (Fig. 4e). LYPLAL1-DT overexpression in EC alleviates migration under both HG and TNF-a treatment (Fig. 4f). These results reflect that LYPLAL1-DT overexpression effectively protects ECs under HG and IS stimulation by enhancing proliferation and migration.

Dysfunctional HUVECs promote vascular inflammation by expressing inflammation cytokines and surface adhesion molecules involved in DMC development. We collected the media of EC-OE under HG and analyzed the concentrations of the secreted cytokines. The results illustrate that the inflammatory molecule IL-1β (Fig. 4g) and the adhesion molecule ICAM-1 decreased (Fig. 4h); conversely, anti-inflammation molecules IL-10 and IL-13 were increased (Fig. 4i) in the EC-OE group and under HG conditions. Consistent with the high level of ICAM-1 in the cell culture media, the overexpression of LYPLAL1-DT significantly suppressed the adhesion of monocytic THP-1 cells onto the HG-treated HUVEC monolayers (Fig. 4j). These results suggest that LYPLAL1-DT plays a significant role in ameliorating the inflammatory conditions in ECs treated with HG.

### LYPLAL1-DT affected SIRT1 expression by acting as a ceRNA sponging miR-204-5p

**Fig. 5:**
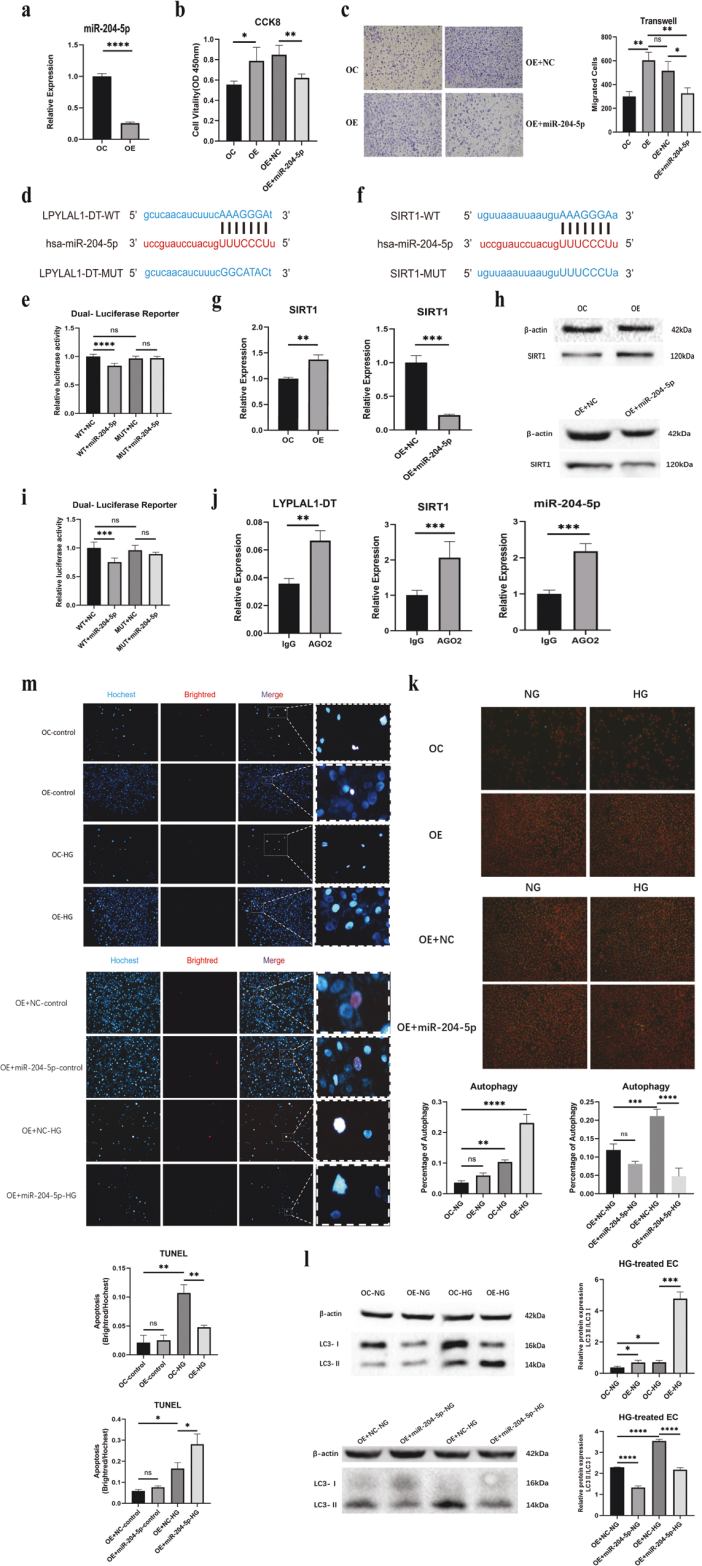
LYPLAL1-DT functions as a sponge and competes with SIRT1 to bind miR-204-5p. **a** Real-time PCR was used to identify the expression levels of miR-204-5p in LYPLAL1-DT-overexpressing cells. **b**, **c** CCK-8 and transwell assays were performed to evaluate the proliferation and migration of EC-OE transfected with miR-204-5p mimic. **d**, **f** The binding sites of miR-204-5p in LYPLAL1-DT and SIRT1 3’UTR were predicted by TargetScan. **e** Luciferase reporter plasmid containing wildtype (WT) or mutant (MUT) LYPLAL1-DT was co-transfected with miR-204-5p mimic into HUVEC. **g**, **h** The SIRT1 expression level was identified by real-time PCR and Western Blot in LYPLAL1-DT-OC/OE and LYPLAL1-DT-OE transfected cells with a miR-204-5p mimic. **i** Luciferase reporter plasmid containing wildtype (WT) or mutant (MUT) SIRT1-3’UTR were co-transfected with miR-204-5p mimic into HUVEC. **j** RIP assays were performed in HUVEC and the RNA co-precipitated with Ago2 and was used to quantify the LYPLAL1-DT, miR-204-5p, and SIRT1 expression levels by real-time PCR. **k** Acridine orange staining, and (**l**) the protein level of LC3 II/I was used to determine the number of autophagy cells under HG condition, and miR-204-5p mimic in LYPLAL1-DT-OC/OE cells. **m** LYPLAL1-DT-OC/OE cells were treated with HG and inspected apoptosis via TUNEL, meanwhile the transfection of miR-204-5p mimic significantly reversed the apoptosis level.

To explore the molecular mechanism of LYPLAL1-DT in the protection of EC, we were able to predict the miRNAs and the corresponding target genes using the bioinformatics tools TargetScan and miRcode. Upon analysis 13 miRNAs with a higher score were detected and found that 3 were upregulated and 2 were downregulated miRNAs in EC-OE compared to the control (Fig. S8). We chose miR-204-5p, the most significantly downregulated miRNA (Fig. 5a) in the EC-OE of LYPLAL1-DT to investigate its function. The miR-204-5p mimic remarkably decreases the proliferation and migration of EC-OE (Fig. 5b, c). Subsequently, we constructed luciferase reporters with both a wild and mutant LYPLAL1-DT site used for miR-204-5p binding (Fig. 5d). The results demonstrated that the mimic miR-204-5p significantly suppresses the luciferase activity of wild type LYPLAL1-DT, compared to either the mutant or empty vector controls (Fig. 5e). We detected 9 target genes of miR-204-5p that were predicted using STARBASE via real-time PCR (Fig. S9), then chose SIRT1 (Fig. 5f) and examined its expression level in EC-OE with and without the presence of the miR-204-5p mimic by real-time PCR and Western blot. SIRT1’s expression was increased in EC-OE and conversely was decreased by the miR-204-5p mimic (Fig. 5g, h). Using a dual luciferase reporter test, it was demonstrated that SIRT1 is the binding targeted gene of miR-204-5p (Fig. 5i). Further results of the RIP assay showed that LYPLAL1-DT, miR-204-5p, and SIRT1 bind to Ago2 (Fig. 5j), confirming that LYPLAL1-DT functions as a competing endogenous RNA (ceRNA) regulating the expression and function of SIRT1 via inhibition with miR-204-5p. SIRT1 is an important gene related to autophagy and apoptosis. Autophagy detected by acridine orange staining showed that LYPLAL1-DT increased the number of autophagy cells under HG condition, and miR-204-5p mimic decreased the number of autophagy cells (Fig. 5k). The protein level of LC3 II/I, the marker of autophagy, showed similar results (Fig. 5l). The apoptotic body under HG conditions demonstrated that LYPLAL1-DT decreased the number of TUNEL positive cells under HG conditions. Meanwhile, the transfection of miR-204-5p mimic significantly reversed the apoptosis level (Fig. 5m). Therefore, our results indicated that lncRNA LYPLAL1-DT inhibits miR-204-5p, consequently upregulating the expression of SIRT1. Furthermore, promoting autophagy, attenuating apoptosis levels in order to alleviate HG injury, and exerting protective effects on ECs.

### LYPLAL1-DT from leukocytes may exert protective effects on HUVEC via exosome transportation

**Fig. 6:**
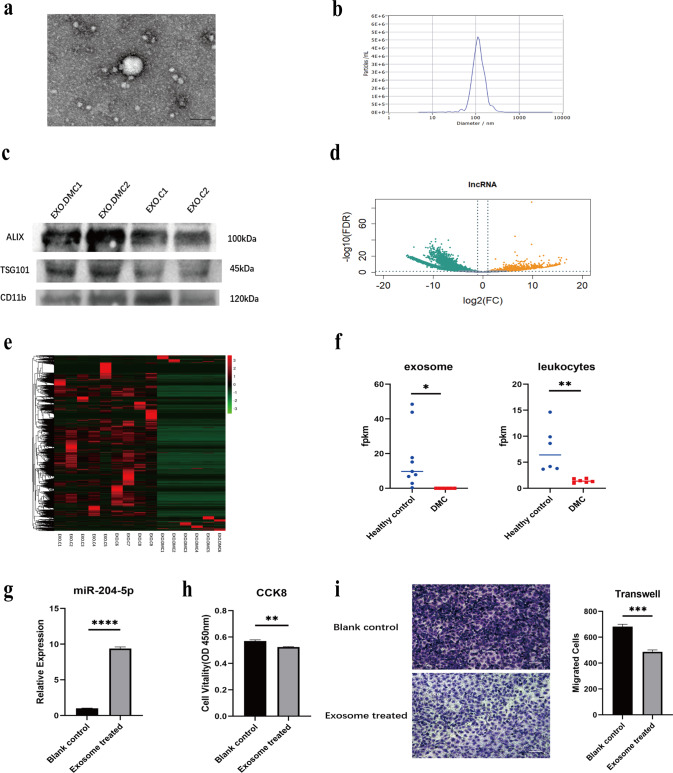
LYPLAL1-DT were transferred from leukocytes to HUVEC via exosomes in DMC patients. **a** Morphology of leukocytes-derived exosomes were shown by TEM. **b** Leukocyte-derived exosomes were observed to be between 30 and 120 nm in size analyzed by NTA. **c** Western Blot was used to examine exosome biomarker ALIX and TSG101, and the leukocyte surface marker CD11b. **d**, **e** Differentially expressed lncRNAs were identified from a Volcano plot and hierarchical cluster analysis, shown as a heatmap (DMC *n* = 6, healthy control *n* = 9). **f** RNA-sequencing of DMC patients-derived exosomes and DMC patients’ leukocytes shows a similar trend of LYPLAL1-DT expression level. **g** The expression of miR-204-5p in HUVECs increased when treated with exosomes derived from DMC patients’ serum compared with control. Exosomes derived from DMC patients’ serum significantly decreased cell viability and migration of HUVEC compared with controls detected by (**h**) CCK8 and (**i**) Transwell assays.

Exosomes regulate the biological functions of recipient cells via RNA transfer. Since LYPLAL1-DT was discovered in leukocytes, we hypothesized that LYPLAL1-DT was transmitted to HUVECs via exosomes. The serum exosomes of DMC patients and healthy controls were extracted, and RNA-sequencing was performed to confirm this hypothesis. The purified exosomes were detected by transmission electron microscopy (TEM) (Fig. 6a), NTA (Fig. 6b), and Western blot (Fig. 6c) using the positive exosome protein markers TSG101 and Alix. The RNA sequencing data showed that DE-lncRNAs (Fig. 6d, e) and DE-mRNAs (Fig. S10a, b) were found between exosomes derived from DMC and control serum, with LYPLAL1-DT having the most significantly different expression levels of the lncRNAs, harboring trends similar to the results found in leukocytes (Fig. 6f). We also detected that the leukocyte cell surface marker CD11b+ is positively expressed in exosomes (Fig. 6c), proving that exosomes may originate from leukocytes. Thus, we treated HUVEC using exosomes collected from DMC patients’ serum and healthy controls and detected miR-204-5p levels, cell viability, and migration ability. As expected, miR-204-5p levels were significantly lower in the control group than in the DMC group (Fig. 6g), while cell viability (Fig. 6h) and migration ability (Fig. 6i) of HUVECs were abbreviated in the DMC group. This data confirmed that expression of LYPLAL1-DT within the exosomes might be derived from the leukocytes affecting the ECs and protecting them from dysfunction.

## Discussion

In the present study, we constructed the transcription profiles of circulating leukocyte lncRNAs and mRNAs in DMC patients and further selected and investigated the molecular mechanisms of a novel lncRNA LYPLAL1-DT having protective effects under pathological conditions. We identified 477 lncRNAs harboring significant differences between patients with diabetic macrovascular complications and healthy controls. This data is more than a microarray analysis of differentially expressed genes screened from the transcriptome of endothelial cells induced by high glucose [24]. Functional analysis and lncRNA-mRNA network analysis showed that it contained many genes that have been reported to be related to diabetes and vascular diseases, such as TOMM5, MYLK, PDLIM1, and CAMK2G [25]. Next, we validated 12 lncRNAs in an expanding cohort. Pathological conditions that were adversely simulated showed that 4 lncRNAs were correlated with EC injury. Finally, lncRNA LYPLAL1-DT was selected and showed protective effects on ECs by combining to miR-204-5p as a ceRNA, thereby regulating the expression of SIRT1, enhancing autophagy, and alleviating EC inflammation. These results strongly suggest that circulating lncRNAs in leukocytes can be an important target for further studies of diabetic vascular complications. Meanwhile, abnormal expression of lncRNA in leukocytes affects ECs, including the adhesion of macrophages, similar to the reports that show lncRNA in the secreted bodies of ECs can adjust to the polarization effect of macrophages [26]. Upon further detection, it was also found that LYPLAL1-DT in exosomes from the serum of DMC patients are derived from leukocytes and is expressed at lower levels than in healthy controls and is similar in leukocytes. All results confirmed that lncRNAs in circulating leukocytes are crucial to the pathogenesis of DMC. Furthermore, LYPLAL1-DT is an important and potential new target for DMC research.

EC stabilization is a pivotal event in the development of diabetes-associated vascular diseases [3]. Sustained high blood glucose in diabetes will continuously stimulate blood vessels, leading to endothelial injury, further stimulating mononuclear cells to accumulate within the blood vessel wall; thus, accompanied by inflammation, and eventually leading to the occurrence of cardiovascular disease [27]. LncRNAs have emerged as critical regulators in a variety of EC biological or pathological processes. Yan B et al. reported that lncRNA-MIAT is involved in angiogenesis and the therapeutics against neovascular diseases [28]. LncRNA MALAT1 is enriched and regulates migration and vascular sprouting of ECs [12]. In this study, ECs were induced by hyperglycemia and inflammatory factors in order to simulate the development of diabetic macrovascular complications. From 12 positively validated lncRNAs in an expanded cohort, 4 lncRNAs were found to change during these treatments. Furthermore, we used a metabolic memory test and found a novel lncRNA LAPLAL1-DT that is sensitive during the stimulating condition as well as returning to normal blood sugar levels, known as hyperglycemic “metabolic memory” in diabetes-related complications [29]. It is reported that eNOS mediates metabolic memory leading to continuous aortic inflammation and endothelial dysfunction [30]; however, evidence of lncRNA involved in EC-metabolic memory is still limited. In this study, we confirmed that lncRNAs in ECs harbor the phenomenon of hyperglycemic metabolic memory. Furthermore, we found that overexpression of LYPLAL1-DT could decrease the adhesion of monophages onto the surface of ECs, enhancing the secretion of anti-inflammatory cytokines IL-10 and IL-13, which is further evidence of the protective effects of EC [31]. These findings suggest that lncRNAs are involved in regulating endothelial cells in the case of diabetes-induced vascular injury and are an important member in the regulation of diabetic macrovascular complications.

In recent years, accumulating evidence highlights a growing list of lncRNAs related to glucose homeostasis and diabetic complications. The molecular mechanisms of lncRNAs can be used as ceRNA in order to regulate target genes by acting as miRNA inhibitors, thus regulating the expression of protein-coding genes [32]. CeRNA plays a vital role in regulating the expression of hyperglycemic response genes. For example, lncRNA ca7-4 ceRNA of miR877-3p and miR5680 promotes autophagy and apoptosis of vascular ECs induced by high glucose [11]. In our study, novel lncRNA LYPLAL1-DT was found to regulate the target gene SIRT1 by acting as a ceRNA of miR-204-5p. SIRT1 is an anti-autophagy factor that can reduce cell death [33], as well as regulate the apoptosis of ECs through the mTOR pathway [34]. It is one of the most important roles in vascular biology and atherogenesis [35]. For instance, SIRT1 can also protect ECs from high glucose induced injury [33]. In the current study, we found SIRT1 is the target gene of LYPLAL1-DT, therefore elevating autophagy levels, thus decreasing apoptosis, inflammation, and monocyte adhesion of ECs. These results are similar to previous reports that SIRT1 inhibits monocyte adhesion to the vascular endothelium [36], and is involved in the inhibitory effects of endothelial cellular apoptosis [37]. More importantly, we confirmed the most recent report that miR-204-5p/SIRT1 mediates inflammation and apoptosis in EC dysfunction under cyanidin-3-O-glucoside treatment [38]. Combined with the report that SIRT1/FoxO1 pathway enhances autophagy flux in order to prevent atherosclerosis and arterial thrombosis [39], we believe SIRT1 plays a critical role in DMC. Hence, we present evidence that LYPLAL1-DT effectively protects against vascular endothelial injury in diabetes associated complications via the LYPLAL1-DT -miR-204-5p/SIRT1 pathway.

There are still some limitations in our study. First, the number of expanded samples for verification is not very large, primarily due to our relatively strict enrollment conditions. We only accepted patients who were initially diagnosed with having diabetic macrovascular complications. Second, how lncRNAs from leukocytes regulate EC function is still largely unknown, although we found that lncRNA LYPLAL1-DT is also present in serum exosomes, with a similar downregulation trend as seen in leukocytes. These exosomes positively expressed leukocytic markers, and partly demonstrates that leukocytes are the primary source of serum exosomes. Moreover, we confirmed that exosomes with lower LYPLAL1-DT could decrease cell viability and migration of ECs. Finally, this study lacks further research on the function of LYPLAL1-DT using an animal model due to the very low conservation of LYPLAL1-DT between humans and mice, as shown in Table 5 and Fig. S7. The species-specific effect of LYPLAL1-DT in DMC remains to be further elucidated. Nevertheless, our observations underscore the importance of LYPLAL1-DT in DMC using human cells.

## Conclusion

In general, we identified 12 DE-lncRNAs related to DMC, among which lncRNA LYPLAL1-DT was identified to be transmitted from leukocytes to ECs via exosomes and have protective effects on EC as ceRNA mediated through the miR-204-5p/SIRT1 pathway. Therefore, inhibiting EC autophagy as well as modulating systemic inflammation. This approach could be regarded as a new potential therapeutic target in DMC.

## Methods

### Subjects

Human blood samples collected from the Department of Endocrinology, Chinese PLA General Hospital, were grouped into either the discovery cohort with healthy control (CTR, *n* = 6), type 2 diabetes mellitus with macrovascular complication (DMC, *n* = 6), a validation cohort with healthy control (CTR, *n* = 36), or type 2 diabetes mellitus with macrovascular complication (DMC, *n* = 46) assessed by clinic examination. All consenting adult subjects (18–65 years old) with no past medical history were consecutively enrolled between July 2016 and April 2018. The whole blood samples were subsequently collected separately in tubes containing RNA after overnight fasting for 10 to 14 h. The subjects in the healthy control group were: (1) healthy with a negative diagnosis of DMC as defined by the World Health Organization (WHO) and with normal blood biochemical indexes; (2) free from all endocrine disease, and (3) aged 18–65 years old without a gender bias. Exclusion criteria were similar to our previous study [40]. The detailed grouped information of all patients were shown in Table 1. The study was approved by the Ethics Committee of Chinese PLA General Hospital (Permitted No. S2016-147-03) and all patients were given informed consent.

### Blood samples collection, exosome extraction, RNA sequencing and analysis

The whole blood sample was collected after fasting for 10–14 h, and total RNA was extracted from peripheral leukocytes as previously reported [40]. Exosomes derived from DMC patients’ serum were extracted via PEG precipitate method via a VEXTM Exosome Isolation Reagent (Vazyme, Nanjing, China). The RNA sequencing process, investigation flow, and data analysis design and methods were similar to our previous work and are shown in Fig. 1A. RNA sequencing was performed by Annoroad Gene Tech. Co., Ltd.

### Cell culture and high glucose, hypoxia, and inflammatory stimulus

Endothelial cell (EC) line human umbilical vein endothelial cells (HUVECs) were purchased from the Shanghai Institute of Cell Biology of the Chinese Academy of Sciences (Shanghai, China) and cultured in RPMI 1640 medium (Corning) at 37 °C with 5% CO2, and supplemented with 10% fetal bovine serum (Vistech). Cells were cultured with a media consisting of 25/30 mM D-glucose (high glucose, HG) or 5 mM D-glucose and 20 mM mannitol (Equilibrium osmotic pressure) (normal glucose, NG). Hypoxia treatment was proceeded by culture cell under 37 °C with 5% CO2. The inflammatory stimulus was performed by adding TNF-α at a concentration of 100 ng/mL. For metabolism memory detection, the cells were first treated with 25 mM glucose for 24 h first, then cultured in a normal medium with a glucose concentration for 72 h. At the end of treatment, the expression levels of the lncRNAs were determined.

### RNA extraction and real-time PCR

For further validation of lncRNA expression from the RNA-sequencing results, a total of 16 lncRNA were measured by real-time PCR in independent expanding cohorts of healthy control (*n* = 36), and DMC (*n* = 46) groups. The full primer list is presented in Supplementary Table S1. Total RNA extraction and PCR were performed as outlined in our previous report [40]. The 2-ΔΔ CT method was used to quantify the relative expression of each lncRNA, using β-actin as an internal control. The relative expression of each examined gene was determined in triplicates. Differences in the lncRNA expressions among the groups were evaluated with a one-way analysis of variance (ANOVA) using SPSS 18.0 software. A significant difference was considered to be *P* < 0.05.

RNA samples from cultured EC were extracted using an RNA isolation Reagent (Vazyme, Nanjing, China). Complementary DNA (cDNA) from 2 μg total RNA was synthesized using 5X All-In-One RT MasterMix (ABM, Zhenjiang, China). The amplification reaction volume was 20 μL and contained 10 μL EvaGreen 2X qPCR MasterMix (ABM, Zhenjiang, China), 1 μL cDNA, 2 μL amplification primers, and 7 μL ddH2O.

Real-time PCR measured the expression levels of the predicted miRNAs and targeted genes in EC. A similar process was used for the peripheral leukocytes. The cDNA of the miRNA was synthesized using a miRNA 1st Strand cDNA Synthesis Kit (Vazyme, Nanjing, China), and real-time PCR was performed using miRNA Universal SYBR qPCR MasterMix (Vazyme, Nanjing, China). The primers for each gene are listed in Supplementary Table S2.

### Construction of lncRNA LYPLAL1-DT overexpression in an EC line

The overexpression of LYPLAL1-DT in HUVECs and the corresponding control cells (OC) were constructed using a pLenti-GIII-CMV-CBH-GFP-2A-Puro vector (ABM, Zhenjiang, China). Cells were transfected for 24 h with a recombinant lentivirus and cultured for 72 h. The transfection efficacy was verified by GFP expression and detected by Real-time PCR.

### Cell transfection

Cells were transfected with miR-204-5p mimic and a negative control (HANBIO, Shanghai, China). MiR-204-5p mimic was transfected at a final concentration of 50 nM via an RNAfit reagent (HANBIO, Shanghai, China), following the manufacturer’s instructions.

### Cell viability, transwell assay, and Western blot

The viability and migration of HUVECs were assessed using CCK8 (Vazyme, Nanjing, China), a transwell assay, and a Wester bolt as performed in our previous studies [18]. The primary antibodies used for Western blot analysis were LC3 (CST), Caspase 3 (CST), β-actin (CST), SIRT1 (CST), Alix (CST), and TSG101 (CST).

### Luciferase assay

To explore the role of the competing endogenous RNA LYPLAL1-DT in ECs, we cloned the 3’-untranslated regions (UTR) of LYPLAL1-DT downstream of the Renilla luciferase gene to generate RLuc- LYPLAL1-DT wild type vector (WT) and the corresponding mutant type vector (MUT); thus, using the firefly luciferase gene as an internal reference. For the luciferase reporter assays, HUVECs were plated in 24-well culture plates, and then transfected with either the WT or the MUT construct with and without miRNA mimic or negative control mimic. Luciferase activities were measured using a Dual Luciferase Reporter Assay System (Vazyme, Nanjing, China), and every transfected well was analyzed in triplicate. The RLuc-SIRT1 wild type vector (WT) was generated to detect relative luciferase expression, following the above experimental steps, in order to discover the relations between miR-204-5p and SIRT1.

### HUVEC-monocyte adhesion and cytokines detection

To detect monocyte-endothelial interactions, in vitro static cell adhesion assays were performed. Human monocytic THP-1 cells were labeled via the fluorescent dye Dil (YEASEN, Shanghai, China). The cells were then co-cultured with HG-treated and TNF-α-stimulated HUVEC in a 6-well plate for 30 min. After washing with PBS three times, the fluorescence signal (red) of the adherent THP-1 cells was quantified under a fluorescence microscope.

Cytokines in the cell culture medium were analyzed using ProcartaPlex Multiplexing Immunoassay kit (eBioscience, USA) using a Luminex 200 (Luminex, USA). Results were calculated using the ProcartaPlex Analyst 1.0 software.

### Apoptosis detection and RNA immunoprecipitation assay (RIP)

The apoptosis level of HG-treated and TNF-α-stimulated HUVEC cells was detected by a TUNEL BrightGreen Apoptosis Detection Kit (Vazyme, Nanjing, China).

RIP was conducted using a Magna RIP RNA-Binding Protein Immunoprecipitation Kit (Millipore, Massachusetts, USA). Briefly, HUVECs were lysed and incubated with magnetic beads conjugated with AGO2 antibodies. After washing with a wash buffer, immunoprecipitated RNA was detected by real-time PCR.

### Acridine orange staining

The HUVEC cells were plated into the confocal dish. After HG treated for 12 h, cells were covered by Acridine orange for 30 min, then washed 3 times by PBS, 5 min each time. The fluorescence signal of the cells was quantified under a fluorescence microscope.

### Statistical analysis

Unless otherwise stated, all experiments were performed at a minimum of triplicates. Statistical analysis was performed using SPSS 19.0 software (SPSS Inc., USA). The data is represented as the mean ± SEM. Comparisons between groups were made using a student’s *t* test between two groups or a one-way ANOVA. Statistically significant differences were set as a *P* < 0.05.

## Supplementary information

Author Contribution Statement

supplementary figures and tables legends

supplementary table 1

supplementary table 2

supplementary table 3

supplementary table 4

supplementary table 5

Figure S1

Figure S2

Figure S3

Figure S4

Figure S5

Figure S6

Figure S7

Figure S8

Figure S9

Figure S10

WB

Cell Line STR Profile Report

## Data Availability

The datasets during and/or analysed during the current study available from the corresponding author on reasonable request (The sequencing data of DMC is in the process of uploading to GEO).
