# Supplementary material for: LncRNA LYPLAL1-DT screening from type 2 diabetes with macrovascular complication contributes protective effects on human umbilical vein endothelial cells via regulating the miR-204-5p/SIRT1 axis
Source: Cell Death Discov. 2022 May 4;8:245. doi: 10.1038/s41420-022-01019-z (PMC9068612; doi:10.1038/s41420-022-01019-z)
Supplement: Supplementary file 1 — Author Contribution Statement [file 41420_2022_1019_MOESM1_ESM.docx]

**Authors’ Contribution**

All authors were responsible for drafting the article and revising it for important intellectual content. They all approved the version to be published.
