# Supplementary material for: LncRNA LYPLAL1-DT screening from type 2 diabetes with macrovascular complication contributes protective effects on human umbilical vein endothelial cells via regulating the miR-204-5p/SIRT1 axis
Source: Cell Death Discov. 2022 May 4;8:245. doi: 10.1038/s41420-022-01019-z (PMC9068612; doi:10.1038/s41420-022-01019-z)
Supplement: Supplementary file 2 — supplementary figures and tables legends [file 41420_2022_1019_MOESM2_ESM.docx]

**Fig. S1** Characteristics of mRNAs transcription in DMC. (a) Differentially expressed mRNAs were identified from a Volcano plot of DMC patients compared to normal controls. The vertical black lines respectively correspond to two-fold up- and down-regulations; and the horizontal black line represents a p-value of 0.05. The red and green points in the plot represent differentially expressed genes with statistical significance for up- and down- regulation, respectively. (b) Differential mRNA expression profiles are hierarchical cluster analyzed and shown as a heatmap, wherein up-regulated genes are depicted in red and down-regulated genes are depicted in green. (c) Principal component analysis also showed the mRNAs are distinguishable between DMC and healthy control.

**Fig. S2** Gene ontology enrichment analysis of mRNAs in DMC. GO analysis of DMC-mRNAs in biological processes, molecular functions, and cellular components.

**Fig. S3** Co-expression networks of lncRNA-mRNA in DMC, consisted with 117 lncRNAs and 392 mRNAs.

**Fig. S4** Characteristics of Novel lncRNAs in DMC. (a) The transcripts of novel lncRNAs were mainly distributed in 2 and 3 exons. (b) Most amount of novel lncRNA transcripts in length are less than 2000 nt. (c) The transcripts of novel lncRNAs are distributed at the conservation score range.

**Fig. S5** The conservation score of novel lncRNAs on each chromosome.

**Fig. S6** Comparison of sequencing data and validation results of 16 lncRNAs. qRT-PCR was used to validate 16 lncRNAs expression levels compared to sequencing data. The white column indicates transcriptome sequencing data, and the black column indicates qRT-PCR amplification verification results. "*" indicates that the expression of lncRNA is significantly different between DMC patients and health controls.

**Fig. S7** LncRNA expression in HUVEC treated with high glucose or induced by hypoxia and the conservation of LYPLAL1-DT. (a) The lncRNAs expression in HUVEC after treated with 30 mM glucose for 12h and 24h. (b) The lncRNAs expression in HUVEC after induced by hypoxia for 12h and 24h. (c) The conservation of LYPLAL1-DT in a verity of species according to UCSC Genome Browser.

**Fig. S8** Predicted miRNAs were quantified in LYPLAL1-DT-OC/OE via qRT-PCR. 13 miRNAs that have potential binding sites with LYPLAL1-DT were predicted by TargetScan and miRcode. “*” indicated significant difference with *p*<0.05, “**” indicated *p*<0.01 and “***” indicated *p*<0.001.

**Fig. S9** Predicted target genes were quantified in LYPLAL1-DT-OC/OE via qRT-PCR. The target mRNAs that have potential binding sites with miR-204-5p were predicted by STARBASE. 9 of 37 predicted genes were quantified in LYPLAL1-DT-OC/OE via qRT-PCR.

**Fig. S10** Differentially expressed mRNAs from exosomes extracted from DMC patients’ serum. DE-mRNAs were identified from a Volcano plot (a) and hierarchical cluster analyzed, shown as a heatmap (b).

Table S1 A total of 16 candidate lncRNAs were further detected in the validation cohorts. The information includes the name, with significant difference in RNA-sequencing data or not, novel/known, up/down, biotype, and primers used in validation by quantitative PCR.

Table S2 The primers information of the predicted miRNAs and targeted gene for the lncRNA LYPLAL1-DT detected in EC under various conditions.

Table S3 A total of 477 significantly differentially expressed lncRNAs (DMC-lncRNAs) obtained from adjusted data.

Table S4. The differential expression of 798 mRNAs (DMC-mRNAs) identified between DMC patients and healthy control.

Table S5 The lncRNAs with predicted genes in the 8 validation positive lncRNAs and their predicted genes.
