# Supplementary material for: LncRNA LYPLAL1-DT screening from type 2 diabetes with macrovascular complication contributes protective effects on human umbilical vein endothelial cells via regulating the miR-204-5p/SIRT1 axis
Source: Cell Death Discov. 2022 May 4;8:245. doi: 10.1038/s41420-022-01019-z (PMC9068612; doi:10.1038/s41420-022-01019-z)
Supplement: Supplementary file 3 — supplementary table 1 [file 41420_2022_1019_MOESM3_ESM.docx]

Table S1 A total of 16 candidate lncRNAs were further detected in the validation cohorts. The information includes the name, with significant difference in RNA-sequencing data or not, novel/known, up/down, biotype, and primers used in validation by quantitative PCR.

| **No.** | **Name** | **Novel/Known** | **Up/**  **Down** | **Biotype** | **With significant difference in RNA-seq or not** | **Forward primer** | **Reverse primer** |
| --- | --- | --- | --- | --- | --- | --- | --- |
| 1 | MSTRG.122492 | Novel | Down | Linc | Yes | GCCCAAGGGAGGATATTCCG | ATATTTCCACCGTGTCCCCC |
| 2 | MSTRG.159131 | Novel | Down | Linc | Yes | TTGTCTTCAGGGAAACGGCT | GGTTTTGGCAAATGCCCTCC |
| 3 | MSTRG.74858 | Novel | Down | Linc | Yes | CCTGGTTAGGGCTGGTTGAA | CCTTCCTTCCTCCACCGTTC |
| 4 | MSTRG.169545 | Novel | Up | Linc | Yes | GCTGATCTGCTTTGAGGCAC | GAGGCATTTGGGGAACCCTC |
| 5 | ENSG00000224307 | Known | Up | Linc | Yes | GTGGAGACACATCTGACGGG | CTGTTGCAGAATGTGGCGAG |
| 6 | MSTRG.95088 | Novel | Down | Linc | Yes | GACCCATGAGAGCCCAAAGT | CCTGCCTGTTACCTCAGAGAG |
| 7 | MSTRG.159327 | Novel | Up | Anti sense | Yes | GCATCCAGATCAGCTTCCTACA | TGTGGGCGTCTGAGAGTTC |
| 8 | ENSG00000269902 | Known | Down | Linc | Yes | AGGTTTAACAGGTCTTACTCCCAA | TTCAGGAGGGGAAATTGAGCC |
| 9 | ENSG00000260244 | Known | Down | Sense overlapping | Yes | TAAGGGTGCAGGGATGGAGA | AGGAGACGGTAAGTCCCTCC |
| 10 | MSTRG.30000 | Novel | Up | Linc | Yes | TGTCTCAGGGAAAGGACAGC | CCTGCATCTACACCCCAACA |
| 11 | ENSG00000274767 | Known | up | Anti sense | Yes | CCTGGCACCAGCACTTTACT | AAATTGCCACAGGTCTGCCT |
|  | MSTRG.63013 | Novel | Up | Linc | Yes | AGGGCCAATACTCAGTCCCT | ATGTCCGGCACTTAGGGAGA |
| 13 | ENSG00000228063 | Known | Down | Anti sense | Yes | CAGCCTCGTGTGGACTTCTG | CTGCAAACATCCTGTGCGAG |
| 14 | MSTRG.131944 | Novel | Down | Anti sense | Yes | AAAGGACAGGTCCTCAAGCC | TCTGGTGGTGGTGACCCATA |
| 15 | MSTRG.152898 | Novel | Up | Anti sense | Yes | TCGCTTGTTAGCGGTCAGTT | ACAATATGCCGCCACAGTCA |
| 16 | MSTRG.3528 | Novel | Up | Linc | Yes | GGGAAGAGGCCAAAGCATGT | TTCCTCCCATTCCACTGACG |
