# Supplementary material for: LncRNA LYPLAL1-DT screening from type 2 diabetes with macrovascular complication contributes protective effects on human umbilical vein endothelial cells via regulating the miR-204-5p/SIRT1 axis
Source: Cell Death Discov. 2022 May 4;8:245. doi: 10.1038/s41420-022-01019-z (PMC9068612; doi:10.1038/s41420-022-01019-z)
Supplement: Supplementary file 4 — supplementary table 2 [file 41420_2022_1019_MOESM4_ESM.docx]

Table S2 The primers information of the predicted miRNAs and targeted gene for the lncRNA LYPLAL1-DT detected in EC under various conditions.

| **No.** | **Name** | **Forward primer** | **Reverse primer** |
| --- | --- | --- | --- |
| 1 | miR-503-3p | CGGGGGTATTGTTTCCGCT | AGTGCAGGGTCCGAGGTATT |
| 2 | miR-141-3p | GCGCGTAACACTGTCTGGTAA | AGTGCAGGGTCCGAGGTATT |
| 3 | miR-200a-3p | GCGCGTAACACTGTCTGGTAA | AGTGCAGGGTCCGAGGTATT |
| 4 | miR-204-3p | CGGCTGGGAAGGCAAAG | AGTGCAGGGTCCGAGGTATT |
| 5 | miR-211-3p | CGCGTTCCCTTTGTCATCCT | AGTGCAGGGTCCGAGGTATT |
| 6 | miR-34a-3p | GCGCGCAATCAGCAAGTATAC | AGTGCAGGGTCCGAGGTATT |
| 7 | miR-449a | CGCGTGGCAGTGTATTGTTA | AGTGCAGGGTCCGAGGTATT |
| 8 | miR-503-5p | CGTAGCAGCGGGAACAGTT | AGTGCAGGGTCCGAGGTATT |
| 9 | miR-204-5p | CGCGTTCCCTTTGTCATCCT | AGTGCAGGGTCCGAGGTATT |
| 10 | miR-211-5p | CGTAGCAGCGGGAACAGTT | AGTGCAGGGTCCGAGGTATT |
| 11 | miR-34a-5p | CGCGTGGCAGTGTCTTAGCT | AGTGCAGGGTCCGAGGTATT |
| 12 | U6 | ACACGCACAAACGAGAAAGG | AGTGCAGGGTCCGAGGTATT |
| 13 | SIRT1 | GACTCCAAGGCCACGGATAG | TGTTCGAGGATCTGTGCCAA |
| 14 | SORT1 | CAACACGCACCAGCATGTGT | CAGTGCTATCTCCAACCCAGG |
| 15 | CTNNBIP1 | CCTATGCAGGGGTGGTCAAC | CGACCTGGAAAACGCCATCA |
| 16 | KHDRBS1 | ATTCTTGGACCACAAGGGAATAC | GCCATAAGAGCATAAGCCTCACA |
| 17 | CCDC6 | GCCGAACTAGAACAGCATCTT | GGGCTGGTCTAATTTTTCCTGC |
| 18 | CAPRIN1 | TCTCGGGGTGATCGACAAGAA | CCCTTTGTTCATTCGTTCCTGG |
| 18 | SDHD | CATCTCTCCACTGGACTAGCG | TCCATCGCAGAGCAAGGATTC |
| 20 | CORO1C | ATGAGGCGAGTGGTACGACA | ATCCCAGGTCACACGAGAAAC |
| 21 | HSPH1 | ACAGCCATGTTGTTGACTAAGC | GCATCTAACACAGATCGCCTCT |
| 22 | TMOD3 | GTGACCTCGCAGCAATTCTTG | GTGGCTCATCAAATACCGGAA |
| 23 | CELSR3 | CCCTCCCTTCAGACTTTTTGATT | CTGTTGCCCATAATTCCCCAC |
| 24 | UBA6 | AGAGGTACGTTCTTGGAGACAC | TGGTTCCTAGATCCCATGCTT |
| 25 | METAP1 | AAGGGATGCGACTTGTATGTAGG | CTTCTTGTAAGGGCCTTCTGTC |
| 26 | FOXC1 | GGCGAGCAGAGCTACTACC | TGCGAGTACACGCTCATGG |
| 27 | β-actin | AGAGGGAAATCGTGCGTGAC | CAATAGTGATGACCTGGCCGT |
