# Supplementary material for: LncRNA LYPLAL1-DT screening from type 2 diabetes with macrovascular complication contributes protective effects on human umbilical vein endothelial cells via regulating the miR-204-5p/SIRT1 axis
Source: Cell Death Discov. 2022 May 4;8:245. doi: 10.1038/s41420-022-01019-z (PMC9068612; doi:10.1038/s41420-022-01019-z)
Supplement: Supplementary file 5 — supplementary table 3 [file 41420_2022_1019_MOESM5_ESM.docx]

Table S3 A total of 477 significantly differentially expressed lncRNAs (DMC-lncRNAs) obtained from adjusted data.

| **Gene** | **Biotype** | **Position** | **C normalize** | **MC normalize** | **FC** | **pval** | **padj** |
| --- | --- | --- | --- | --- | --- | --- | --- |
| ENSG00000273338 | antisense | chr1:78004346-78004554:- | 109.992284 | 10.19783764 | 0.092714118 | 9.58E-16 | 4.85E-12 |
| MSTRG.74858 | linc | chr17:83203319-83204570:+ | 82.05959434 | 5.997176225 | 0.073083181 | 6.71E-15 | 2.27E-11 |
| MSTRG.179846 | antisense | chr9:40059342-40106069:- | 1146.593605 | 289.9478211 | 0.252877584 | 5.98E-15 | 2.20E-11 |
| ENSG00000270069 | lincRNA | chrX:45745211-45770274:- | 441.7709818 | 78.99492204 | 0.178814194 | 1.12E-13 | 3.48E-10 |
| MSTRG.159146 | antisense | chr7:2677531-2679790:- | 41.00730088 | 1.860109247 | 0.045360441 | 2.01E-12 | 4.07E-09 |
| MSTRG.131944 | antisense | chr4:102007151-102018762:+ | 2338.924568 | 790.9080039 | 0.338150283 | 1.39E-10 | 2.01E-07 |
| MSTRG.103146 | antisense | chr20:17570371-17583003:+ | 630.4337965 | 189.1499066 | 0.300031356 | 3.05E-10 | 3.87E-07 |
| ENSG00000279098 | TEC | chr4:99942081-99942522:- | 91.30309797 | 16.31621502 | 0.178703849 | 7.07E-10 | 8.18E-07 |
| MSTRG.80841 | linc | chr19:10959257-10960669:- | 1.106885237 | 23.32966687 | 21.07686153 | 8.69E-10 | 9.48E-07 |
| MSTRG.182419 | linc | chr9:91193131-91198833:+ | 92.96624968 | 17.29148431 | 0.185997439 | 1.67E-09 | 1.65E-06 |
| MSTRG.159131 | linc | chr7:2675866-2677134:- | 27.58303785 | 1.692678294 | 0.061366638 | 3.68E-09 | 3.24E-06 |
| MSTRG.187859 | linc | chrX:20117156-20123616:- | 186.5645545 | 48.11364749 | 0.257892758 | 2.19E-08 | 1.53E-05 |
| MSTRG.106807 | antisense | chr21:15047465-15064494:- | 3095.658423 | 1060.73299 | 0.342651819 | 2.04E-08 | 1.45E-05 |
| ENSG00000251022 | antisense | chr4:82893009-82900960:- | 1004.098601 | 401.3544598 | 0.399716183 | 1.98E-08 | 1.43E-05 |
| MSTRG.111791 | linc | chr22:41419090-41428731:+ | 29.39530367 | 129.0863667 | 4.391394221 | 4.17E-08 | 2.71E-05 |
| ENSG00000270640 | Sense intronic | chr2:28396815-28397110:+ | 167.6875962 | 39.20189834 | 0.233779357 | 5.26E-08 | 3.14E-05 |
| MSTRG.95088 | linc | chr2:144662307-144663366:- | 12.15353927 | 0.166314414 | 0.013684443 | 7.88E-08 | 4.43E-05 |
| MSTRG.116382 | linc | chr3:49355335-49356922:- | 324.3570158 | 107.2770929 | 0.330737698 | 1.13E-07 | 6.04E-05 |
| ENSG00000272888 | Processed transcript | chr15:92882707-92899701:+ | 5073.848846 | 2353.336191 | 0.463816771 | 2.88E-07 | 0.000129867 |
| ENSG00000224307 | lincRNA | chr9:129282458-129285728:+ | 38.24345122 | 171.6087033 | 4.487270311 | 3.15E-07 | 0.000136 |
| ENSG00000271347 | sense intronic | chr15:24991486-24991753:+ | 227.1826196 | 79.29343294 | 0.349029486 | 3.45E-07 | 0.000141113 |
| MSTRG.30000 | linc | chr11:47452518-47462678:- | 75.03619863 | 242.8552294 | 3.23650763 | 4.14E-07 | 0.00015964 |
| ENSG00000269902 | lincRNA | chrX:45764772-45765299:- | 88.15192677 | 12.61321734 | 0.143084988 | 3.99E-07 | 0.000155369 |
| MSTRG.183281 | antisense | chr9:105707806-105713053:+ | 353.2896758 | 137.4637905 | 0.38909654 | 4.47E-07 | 0.00017111 |
| MSTRG.185495 | linc | chr9:128668273-128670326:- | 1.29032532 | 17.8105971 | 13.80318345 | 7.58E-07 | 0.000258338 |
| ENSG00000274767 | antisense | chr17:36183235-36196471:+ | 120.0985866 | 341.0418251 | 2.839682254 | 8.15E-07 | 0.000270632 |
| MSTRG.126704 | linc | chr4:6886155-6888188:+ | 9.656941263 | 57.7853465 | 5.98381464 | 8.77E-07 | 0.000288868 |
| MSTRG.159327 | antisense | chr7:6405349-6450822:+ | 103.8118475 | 364.177875 | 3.508056969 | 9.26E-07 | 0.000297619 |
| ENSG00000279463 | sense intronic | chr13:73844683-73845130:- | 125.9449669 | 38.86925899 | 0.308620979 | 1.01E-06 | 0.000315736 |
| MSTRG.72007 | antisense | chr17:49230163-49230827:+ | 16.65933282 | 72.56739819 | 4.355960647 | 1.28E-06 | 0.00037799 |
| MSTRG.60283 | antisense | chr15:64506449-64592774:+ | 0.637727305 | 11.8932794 | 18.64947496 | 1.25E-06 | 0.000371451 |
| MSTRG.165064 | antisense | chr7:103328437-103334335:- | 212.4254566 | 65.32388664 | 0.307514399 | 1.77E-06 | 0.000484447 |
| MSTRG.167609 | antisense | chr7:150450476-150451324:- | 4.770894955 | 30.22815074 | 6.335949759 | 1.95E-06 | 0.000526859 |
| MSTRG.80933 | antisense | chr19:11315420-11320164:- | 60.24348927 | 346.5193851 | 5.751980658 | 2.21E-06 | 0.000581711 |
| ENSG00000274422 | lincRNA | chr22:22283928-22287220:- | 147.3139036 | 46.63109327 | 0.316542377 | 2.43E-06 | 0.000620663 |
| MSTRG.80769 | linc | chr19:10347759-10348204:- | 0.783050484 | 12.13997328 | 15.50343628 | 2.83E-06 | 0.000695469 |
| ENSG00000237491 | lincRNA | chr1:778770-810060:+ | 208.7383097 | 74.45546059 | 0.35669284 | 2.81E-06 | 0.000695469 |
| MSTRG.38036 | linc | chr12:31995136-32037418:+ | 511.8672699 | 1613.771772 | 3.152715297 | 3.08E-06 | 0.000722536 |
| MSTRG.95087 | linc | chr2:144660953-144665439:+ | 98.88683746 | 16.2188283 | 0.164014026 | 3.34E-06 | 0.000760802 |
| ENSG00000267257 | antisense | chr18:58535415-58538552:+ | 20.44380376 | 0.48388371 | 0.023668967 | 3.84E-06 | 0.000850572 |
| ENSG00000267174 | 3prime overlapping ncRNA | chr19:11300777-11324441:- | 51.20143325 | 329.7040936 | 6.439352819 | 4.07E-06 | 0.000896496 |
| MSTRG.151472 | antisense | chr6:41146287-41178223:+ | 428.6435186 | 171.5138669 | 0.400131717 | 4.75E-06 | 0.001017983 |
| ENSG00000186594 | lincRNA | chr17:1711493-1717174:- | 810.3461893 | 348.6208558 | 0.430212248 | 4.94E-06 | 0.001049408 |
| MSTRG.147880 | linc | chr5:178650106-178668781:- | 0.196102039 | 16.78214887 | 85.57865546 | 5.43E-06 | 0.001121592 |
| ENSG00000276136 | lincRNA | chr12:32000375-32001222:+ | 65.48854156 | 207.9449251 | 3.175287159 | 5.37E-06 | 0.001116907 |
| MSTRG.46436 | antisense | chr13:31189540-31200026:- | 179.5336924 | 67.84561245 | 0.377899054 | 6.69E-06 | 0.001284883 |
| ENSG00000255240 | antisense | chr11:58933643-59058659:- | 259.7352023 | 95.35105429 | 0.367108707 | 8.15E-06 | 0.001507949 |
| ENSG00000229368 | sense overlapping | chr11:3854612-3855399:+ | 13.69282679 | 56.96263058 | 4.160034408 | 8.69E-06 | 0.001558269 |
| ENSG00000278900 | TEC | chr5:34105750-34106336:- | 67.09895749 | 16.68206607 | 0.248618856 | 9.16E-06 | 0.001599633 |
| MSTRG.556 | linc | chr1:7863299-7913280:- | 221.2641064 | 688.434856 | 3.111371597 | 9.10E-06 | 0.001596954 |
| MSTRG.24583 | linc | chr10:111147684-111150843:- | 46.37883406 | 7.648254943 | 0.164908306 | 9.76E-06 | 0.001689828 |
| ENSG00000223692 | sense intronic | chr21:46462471-46469306:+ | 166.7149301 | 60.59413188 | 0.36345954 | 1.05E-05 | 0.001788078 |
| MSTRG.135989 | linc | chr4:184858715-184861916:- | 37.36629713 | 4.862846982 | 0.130139922 | 1.47E-05 | 0.002383723 |
| MSTRG.157175 | linc | chr6:141831065-142030439:- | 39.47840691 | 7.524962997 | 0.190609591 | 1.46E-05 | 0.002376204 |
| ENSG00000270022 | lincRNA | chr22:42615244-42615907:+ | 87.92996657 | 26.20111321 | 0.297977063 | 1.45E-05 | 0.002363496 |
| ENSG00000269967 | lincRNA | chr1:31851913-31921841:- | 163.6980835 | 62.05728062 | 0.379095951 | 1.86E-05 | 0.00285195 |
| MSTRG.129614 | antisense | chr4:56412310-56415249:- | 54.64393639 | 12.88024201 | 0.235712192 | 1.85E-05 | 0.00285195 |
| ENSG00000275560 | sense intronic | chr12:12718973-12719521:+ | 593.2041306 | 279.5193753 | 0.471202679 | 1.82E-05 | 0.00285195 |
| MSTRG.182748 | antisense | chr9:96402030-96414736:- | 64.14384037 | 18.74952648 | 0.292304395 | 2.04E-05 | 0.003042995 |
| MSTRG.27302 | antisense | chr11:11848936-11861282:+ | 2008.040211 | 871.8773726 | 0.434193184 | 2.13E-05 | 0.003135061 |
| ENSG00000235499 | lincRNA | chr2:73985132-73986343:+ | 21.13358453 | 72.23975012 | 3.418244076 | 2.28E-05 | 0.003277327 |
| ENSG00000227165 | antisense | chr10:120761812-120851345:- | 446.8632539 | 174.6189233 | 0.390765904 | 2.47E-05 | 0.003474085 |
| MSTRG.60159 | linc | chr15:69719165-69720682:+ | 51.98879102 | 9.856065849 | 0.189580593 | 2.47E-05 | 0.003474085 |
| MSTRG.180334 | linc | chr9:61465105-61467945:+ | 0.626045558 | 10.24880532 | 16.3707021 | 2.56E-05 | 0.003532523 |
| MSTRG.96702 | antisense | chr2:171928087-171934889:+ | 284.3946351 | 109.533803 | 0.38514722 | 3.23E-05 | 0.004047233 |
| ENSG00000278330 | antisense | chr18:77112602-77115726:+ | 23.66776924 | 4.329970327 | 0.18294797 | 3.21E-05 | 0.00403478 |
| MSTRG.21467 | linc | chr10:69058405-69062276:- | 27.82443982 | 5.079486732 | 0.182554861 | 3.18E-05 | 0.00403478 |
| ENSG00000130600 | processed transcript | chr11:1995163-2001470:- | 13.51286343 | 1.654716909 | 0.122454942 | 3.31E-05 | 0.004113816 |
| MSTRG.101154 | linc | chr2:231595258-231604693:- | 86.84477013 | 25.07484871 | 0.288731822 | 3.14E-05 | 0.0040157 |
| ENSG00000277767 | lincRNA | chr13:110916004-110917827:+ | 57.93621496 | 16.53727746 | 0.285439383 | 2.94E-05 | 0.003873704 |
| ENSG00000279693 | TEC | chr16:68367325-68370262:- | 7.791189935 | 40.98044363 | 5.259843999 | 2.91E-05 | 0.00386615 |
| MSTRG.5412 | linc | chr1:65034964-65038215:- | 51.08989564 | 257.539942 | 5.040917363 | 3.13E-05 | 0.0040157 |
| MSTRG.175987 | linc | chr8:122338742-122379443:- | 7.388104288 | 0.317569296 | 0.042983868 | 3.06E-05 | 0.003955485 |
| MSTRG.139451 | linc | chr5:57169734-57173724:- | 124.5523237 | 44.92859748 | 0.360720668 | 3.11E-05 | 0.004001603 |
| ENSG00000259687 | lincRNA | chr14:75294404-75296638:+ | 132.4121656 | 44.59560438 | 0.33679386 | 2.91E-05 | 0.00386615 |
| MSTRG.67600 | linc | chr16:88539974-88556470:+ | 113.7516415 | 396.0217442 | 3.481459597 | 3.04E-05 | 0.003948368 |
| MSTRG.192676 | linc | chrX:107636256-107677157:- | 230.9218876 | 532.1949172 | 2.304653417 | 2.83E-05 | 0.00382099 |
| MSTRG.21851 | linc | chr10:72237497-72241833:+ | 34.54329019 | 105.8812725 | 3.065176245 | 2.87E-05 | 0.003858128 |
| ENSG00000235609 | lincRNA | chr21:14818843-15014430:- | 86.81695121 | 27.05473775 | 0.311629669 | 3.65E-05 | 0.00444299 |
| MSTRG.90046 | linc | chr2:57614497-57646095:- | 437.3273774 | 189.1727363 | 0.432565501 | 3.64E-05 | 0.00444299 |
| MSTRG.15821 | linc | chr1:228409811-228424799:+ | 52.34723603 | 144.0069327 | 2.750994008 | 4.24E-05 | 0.004944052 |
| ENSG00000237357 | lincRNA | chr9:42566679-42569353:- | 13.73307522 | 1.393050132 | 0.101437596 | 4.22E-05 | 0.00493006 |
| ENSG00000258820 | antisense | chr14:75259411-75271950:+ | 296.0300549 | 63.45636988 | 0.214357863 | 4.37E-05 | 0.005046124 |
| MSTRG.68487 | antisense | chr17:5240431-5241142:+ | 9.879738956 | 0.842568969 | 0.085282513 | 4.87E-05 | 0.005398123 |
| MSTRG.142970 | linc | chr5:109869957-109870453:+ | 3.804784557 | 21.09912412 | 5.545418882 | 4.82E-05 | 0.005398123 |
| MSTRG.148587 | linc | chr6:3455372-3469349:+ | 79.76484869 | 24.13268535 | 0.302547873 | 5.26E-05 | 0.005687527 |
| MSTRG.71291 | antisense | chr17:42026723-42027293:+ | 0.959113862 | 10.39965727 | 10.84298505 | 5.57E-05 | 0.00597198 |
| MSTRG.93076 | linc | chr2:111187202-111187998:+ | 0.345730262 | 6.695179204 | 19.36532592 | 5.93E-05 | 0.00624881 |
| MSTRG.363 | antisense | chr1:779512-781826:+ | 76.97840132 | 25.88277607 | 0.336234264 | 6.00E-05 | 0.006289145 |
| ENSG00000215533 | sense overlapping | chr21:29193480-29288205:+ | 6.541682705 | 33.42209789 | 5.10909798 | 5.91E-05 | 0.00624881 |
| ENSG00000254138 | antisense | chr5:31093977-31267610:- | 30.65414484 | 6.192121073 | 0.201999472 | 6.66E-05 | 0.006829328 |
| MSTRG.30136 | linc | chr11:62025111-62029411:+ | 280.0646495 | 74.12845585 | 0.264683372 | 7.32E-05 | 0.007253554 |
| MSTRG.125714 | antisense | chr3:196215032-196228373:- | 9.189308055 | 35.77546257 | 3.893161743 | 8.04E-05 | 0.007779989 |
| MSTRG.169545 | linc | chr8:21893316-21894352:- | 4.042603263 | 21.95721132 | 5.431453421 | 7.98E-05 | 0.007755496 |
| MSTRG.40287 | antisense | chr12:64699215-64741058:+ | 346.4254979 | 888.6435654 | 2.565179442 | 8.28E-05 | 0.00791734 |
| ENSG00000231528 | lincRNA | chr9:113113073-113119928:+ | 0.957257015 | 9.634278322 | 10.06446354 | 7.98E-05 | 0.007755496 |
| MSTRG.159431 | linc | chr7:6103517-6104660:- | 10.66319389 | 40.21014189 | 3.770928512 | 8.27E-05 | 0.00791734 |
| MSTRG.10587 | antisense | chr1:156211503-156218677:- | 4.893610335 | 22.65806234 | 4.630132109 | 8.66E-05 | 0.008167972 |
| MSTRG.81594 | antisense | chr19:18282200-18285604:+ | 115.8087339 | 43.19676179 | 0.373000898 | 8.84E-05 | 0.008257218 |
| MSTRG.182532 | linc | chr9:93044764-93049979:+ | 5.800205024 | 35.07347278 | 6.046936726 | 8.79E-05 | 0.008247096 |
| MSTRG.52421 | antisense | chr14:45116582-45119451:+ | 7.720930461 | 31.01050608 | 4.01642085 | 9.10E-05 | 0.008437021 |
| MSTRG.128496 | antisense | chr4:26947134-26992747:- | 11.74765661 | 1.350147925 | 0.114929128 | 9.43E-05 | 0.008671062 |
| ENSG00000248510 | lincRNA | chr4:96310701-96818864:+ | 11.31805015 | 1.160138266 | 0.102503369 | 0.000101115 | 0.009108221 |
| MSTRG.65832 | antisense | chr16:66471095-66492777:- | 10.23876545 | 44.62025589 | 4.357972267 | 0.000101006 | 0.009108221 |
| MSTRG.46819 | linc | chr13:37089912-37091146:- | 7.008028453 | 51.2933889 | 7.319232398 | 0.000107003 | 0.009470269 |
| MSTRG.66083 | antisense | chr16:67998699-68001317:+ | 154.5703275 | 378.1667172 | 2.446567354 | 0.000106349 | 0.009432988 |
| ENSG00000275413 | antisense | chr17:16023323-16023653:- | 129.4251344 | 52.16825047 | 0.403076657 | 0.00010568 | 0.009394188 |
| MSTRG.147524 | antisense | chr5:175478722-175489613:+ | 379.3674719 | 170.6346308 | 0.449787194 | 0.000105264 | 0.009377749 |
| MSTRG.148346 | linc | chr6:2798034-2799059:+ | 2.77578193 | 15.80047461 | 5.69226078 | 0.00010433 | 0.009335536 |
| MSTRG.54498 | linc | chr14:75298906-75303521:+ | 99.16548412 | 28.48964558 | 0.28729397 | 0.000111518 | 0.009826903 |
| MSTRG.65777 | linc | chr16:56984544-56989411:- | 80.65520231 | 195.8162531 | 2.427819253 | 0.000113149 | 0.009906074 |
| MSTRG.135825 | linc | chr4:183332270-183333587:- | 14.97080116 | 0.679335583 | 0.04537737 | 0.000120923 | 0.010406784 |
| ENSG00000280181 | TEC | chr12:64709458-64710513:+ | 15.8314956 | 59.32388499 | 3.747206612 | 0.00012805 | 0.010743438 |
| ENSG00000276853 | sense intronic | chr12:65171262-65171917:+ | 162.3140684 | 67.52475212 | 0.416012936 | 0.000123887 | 0.010594431 |
| MSTRG.172596 | linc | chr8:73878959-73882396:+ | 45.66406307 | 11.30582106 | 0.24758684 | 0.000128034 | 0.010743438 |
| MSTRG.43453 | antisense | chr12:107757251-107758229:- | 118.1090955 | 42.04999563 | 0.356026735 | 0.000128003 | 0.010743438 |
| MSTRG.45475 | linc | chr13:19788366-19790913:- | 0.167612353 | 5.377919718 | 32.0854617 | 0.000127332 | 0.010743438 |
| ENSG00000253394 | lincRNA | chr8:90221488-90569318:+ | 77.31998571 | 21.92812839 | 0.283602334 | 0.000132158 | 0.010955099 |
| MSTRG.72904 | linc | chr17:61935496-61937631:- | 1.000484677 | 9.232551341 | 9.228078701 | 0.000133521 | 0.011022924 |
| MSTRG.156704 | antisense | chr6:136288906-136293334:+ | 640.7150031 | 311.4453008 | 0.486090226 | 0.000137077 | 0.01124777 |
| MSTRG.44383 | antisense | chr12:121646167-121670989:+ | 9.338225248 | 44.13943978 | 4.726748243 | 0.00013937 | 0.011321366 |
| ENSG00000276216 | lincRNA | chr1:145281116-145281462:+ | 109.9776656 | 29.40239605 | 0.267348792 | 0.00013827 | 0.011299943 |
| ENSG00000273989 | sense intronic | chr12:28236227-28236828:+ | 64.09753149 | 21.14931152 | 0.329955164 | 0.000141971 | 0.011418231 |
| MSTRG.154388 | antisense | chr6:106297287-106304946:- | 610.6408198 | 291.6143775 | 0.477554674 | 0.000153141 | 0.01203016 |
| ENSG00000237667 | lincRNA | chr2:779840-868426:- | 11.26976165 | 1.186070975 | 0.105243661 | 0.000159077 | 0.012376554 |
| MSTRG.163109 | linc | chr7:66520026-66592388:- | 156.3128377 | 332.615486 | 2.127883358 | 0.00016329 | 0.012654626 |
| ENSG00000267316 | lincRNA | chr18:61571342-61579456:- | 21.80865532 | 4.464264841 | 0.204701518 | 0.000172215 | 0.013070371 |
| ENSG00000261367 | antisense | chr16:30107675-30110541:+ | 0.196102039 | 6.373714224 | 32.50202925 | 0.000191835 | 0.014215786 |
| MSTRG.179077 | linc | chr9:27589073-27591369:- | 93.40822756 | 19.58346249 | 0.209654578 | 0.000194367 | 0.014350928 |
| MSTRG.3528 | linc | chr1:43372242-43372779:+ | 2.078778359 | 12.94910165 | 6.229188214 | 0.000200966 | 0.014730837 |
| MSTRG.81088 | antisense | chr19:13172244-13218650:+ | 24.08739488 | 71.80851682 | 2.981165758 | 0.00020589 | 0.015026199 |
| ENSG00000233096 | lincRNA | chr3:40970541-40971578:- | 5.727915708 | 0.173531623 | 0.030295771 | 0.000208693 | 0.01508211 |
| MSTRG.94873 | linc | chr2:142831612-142871808:- | 270.2779267 | 124.193928 | 0.459504517 | 0.000214374 | 0.015368739 |
| MSTRG.111444 | linc | chr22:37895975-37897670:+ | 0.502735189 | 7.305269727 | 14.53104913 | 0.000219043 | 0.015631916 |
| MSTRG.154348 | linc | chr6:105676852-105892943:- | 3.905533425 | 24.10628416 | 6.172341018 | 0.000226287 | 0.016007963 |
| MSTRG.35821 | antisense | chr12:1796345-1797664:+ | 22.2404684 | 67.05000238 | 3.014774741 | 0.000232274 | 0.016261184 |
| MSTRG.138541 | linc | chr5:39074483-39077107:+ | 39.48430686 | 120.5320697 | 3.052657606 | 0.000236855 | 0.016521976 |
| MSTRG.154253 | linc | chr6:105453557-105480939:+ | 225.4079046 | 74.56611701 | 0.330805245 | 0.000246881 | 0.016961553 |
| ENSG00000271614 | lincRNA | chr12:89708959-89712590:+ | 710.8891014 | 301.9311942 | 0.424723341 | 0.00025664 | 0.017542864 |
| MSTRG.104418 | linc | chr20:35742519-35746546:+ | 345.7438566 | 172.8607423 | 0.499967647 | 0.000258981 | 0.017673044 |
| ENSG00000278462 | sense intronic | chr13:46717423-46717688:+ | 37.0082855 | 10.85501196 | 0.293313019 | 0.000264838 | 0.017837227 |
| MSTRG.163211 | antisense | chr7:66743826-66755201:+ | 2049.027178 | 834.8162989 | 0.407420803 | 0.000263617 | 0.017809541 |
| ENSG00000265206 | antisense | chr17:58330884-58332508:- | 289.9957042 | 608.4415121 | 2.098105259 | 0.000280743 | 0.01850393 |
| ENSG00000245149 | lincRNA | chr8:124462485-124474576:- | 175.3745172 | 79.43695432 | 0.452956083 | 0.000280284 | 0.01850393 |
| ENSG00000237513 | lincRNA | chr7:104941063-104962334:+ | 219.2656196 | 105.7817849 | 0.482436713 | 0.000279633 | 0.01850393 |
| MSTRG.108623 | linc | chr21:42784049-42792158:+ | 5.633671401 | 30.95911269 | 5.495370688 | 0.000290719 | 0.018976318 |
| ENSG00000261043 | lincRNA | chr15:75759501-75762405:- | 1.166161504 | 28.73112581 | 24.63734716 | 0.000292945 | 0.019015696 |
| MSTRG.71750 | antisense | chr17:45431635-45432932:+ | 0.353106966 | 5.584001489 | 15.81390916 | 0.000302289 | 0.019175691 |
| MSTRG.37943 | antisense | chr12:31305863-31323892:- | 1984.550466 | 935.8710942 | 0.47157838 | 0.000298332 | 0.019175691 |
| ENSG00000225938 | antisense | chr1:101235683-101236528:- | 20.85934052 | 62.42932768 | 2.992871593 | 0.000296568 | 0.019111895 |
| MSTRG.49388 | linc | chr13:88709424-88744650:+ | 15.63895917 | 2.462935905 | 0.157487201 | 0.000302118 | 0.019175691 |
| MSTRG.163209 | linc | chr7:67341881-67345699:+ | 4.86282062 | 23.07923381 | 4.74605905 | 0.000314875 | 0.019703565 |
| ENSG00000261455 | lincRNA | chr7:152463786-152465549:+ | 135.9916862 | 59.82815746 | 0.439939816 | 0.000324935 | 0.020164664 |
| ENSG00000261441 | antisense | chr15:89335053-89336161:+ | 2.641431848 | 13.73694714 | 5.200568453 | 0.000332739 | 0.020435701 |
| MSTRG.69585 | linc | chr17:19898607-19901969:- | 166.8588676 | 337.4206752 | 2.022192048 | 0.000331736 | 0.020405019 |
| MSTRG.64351 | linc | chr16:28331011-28334012:+ | 4.329275744 | 18.55132972 | 4.285088503 | 0.000344662 | 0.020945832 |
| ENSG00000225195 | sense intronic | chr12:64628344-64629976:+ | 24.94070489 | 70.45428887 | 2.824871598 | 0.000348432 | 0.021034672 |
| MSTRG.72756 | linc | chr17:59408838-59433609:+ | 29.28709239 | 81.12470361 | 2.769981483 | 0.000369459 | 0.022023563 |
| MSTRG.113416 | linc | chr3:9487946-9490058:+ | 1.842371383 | 12.30007651 | 6.676219909 | 0.000383728 | 0.022544071 |
| ENSG00000279416 | TEC | chr18:79085134-79086435:+ | 90.69590789 | 31.26740062 | 0.34474985 | 0.000381875 | 0.022499001 |
| MSTRG.117056 | linc | chr3:57937488-57946101:+ | 24.66237921 | 68.61888103 | 2.782330141 | 0.000391479 | 0.022898422 |
| MSTRG.38028 | linc | chr12:31865838-31870589:- | 18.70463575 | 56.5129988 | 3.021336505 | 0.000399659 | 0.023196895 |
| MSTRG.144110 | linc | chr5:123721820-124204494:+ | 240.0990615 | 54.62779819 | 0.227521915 | 0.000398318 | 0.023164727 |
| ENSG00000230606 | lincRNA | chr2:97416165-97433527:- | 286.941188 | 122.6202123 | 0.427335696 | 0.000406913 | 0.023296912 |
| MSTRG.147689 | antisense | chr5:176872188-176880564:- | 167.7397237 | 441.898731 | 2.634431017 | 0.000413505 | 0.023508329 |
| ENSG00000269906 | sense intronic | chr14:50662511-50663178:- | 24.62832238 | 6.578535217 | 0.2671126 | 0.000412655 | 0.02349291 |
| ENSG00000267765 | antisense | chr17:42683187-42699466:- | 4.968906962 | 20.89554493 | 4.205259846 | 0.000423587 | 0.023913805 |
| ENSG00000276334 | sense intronic | chr2:32521927-32523547:+ | 215.6938037 | 103.8639785 | 0.481534364 | 0.000423244 | 0.023913805 |
| MSTRG.157484 | linc | chr6:148617700-148624630:+ | 124.4245305 | 49.90913699 | 0.401119753 | 0.000429274 | 0.024201161 |
| MSTRG.135826 | linc | chr4:183334139-183334525:- | 8.131247033 | 0.502420966 | 0.061788919 | 0.000439277 | 0.024594053 |
| MSTRG.170575 | linc | chr8:38061085-38062605:+ | 3.912848398 | 27.65251305 | 7.067105657 | 0.000442475 | 0.024670894 |
| MSTRG.28819 | antisense | chr11:35246663-35258058:+ | 148.089612 | 28.95757149 | 0.19554087 | 0.000445777 | 0.024740274 |
| ENSG00000231826 | lincRNA | chr2:43027853-43039547:- | 5.044788863 | 21.03544677 | 4.169737791 | 0.000456644 | 0.025115421 |
| ENSG00000236772 | antisense | chr20:32449755-32453607:+ | 1.467251606 | 9.671428034 | 6.59152663 | 0.000460326 | 0.025283651 |
| MSTRG.31454 | linc | chr11:72793790-72810827:+ | 10.91596446 | 36.06377279 | 3.303764218 | 0.000468701 | 0.025501713 |
| MSTRG.59140 | antisense | chr15:55169618-55354124:- | 173.856187 | 348.501392 | 2.004538337 | 0.000494634 | 0.026524101 |
| ENSG00000236535 | sense intronic | chr1:174009267-174016206:- | 29.37337166 | 84.80725493 | 2.887215534 | 0.000492271 | 0.026524101 |
| MSTRG.94162 | antisense | chr2:127445820-127461574:- | 8.989195925 | 31.44992835 | 3.498636431 | 0.000489696 | 0.026501939 |
| ENSG00000248538 | lincRNA | chr8:9189011-9202854:+ | 8.152976064 | 0.491820762 | 0.060324078 | 0.00050254 | 0.026803261 |
| ENSG00000272053 | lincRNA | chr6:25014952-25042170:- | 848.1300726 | 399.5795702 | 0.471130058 | 0.000520237 | 0.027351229 |
| MSTRG.110441 | linc | chr22:29779652-29781311:- | 0.508255045 | 5.908637512 | 11.62533961 | 0.000540486 | 0.027838107 |
| MSTRG.122909 | antisense | chr3:151262011-151273554:- | 327.512831 | 775.1632976 | 2.366818104 | 0.00053529 | 0.027676012 |
| ENSG00000273565 | lincRNA | chr14:75176929-75177418:+ | 25.91455268 | 6.992816151 | 0.269841283 | 0.000530299 | 0.027593928 |
| MSTRG.179923 | linc | chr9:40855131-40874104:- | 124.032482 | 54.38631739 | 0.438484472 | 0.000540131 | 0.027838107 |
| MSTRG.158115 | antisense | chr6:156779721-156824457:+ | 1709.221326 | 770.9513316 | 0.451054126 | 0.000534503 | 0.027670577 |
| ENSG00000240893 | lincRNA | chr3:112736447-112749319:- | 10.78785283 | 1.865445483 | 0.172920924 | 0.000554845 | 0.028254568 |
| MSTRG.30578 | antisense | chr11:65768007-65772614:- | 25.32955111 | 105.8745009 | 4.179880662 | 0.000564518 | 0.028639204 |
| ENSG00000215417 | processed transcript | chr13:91347820-91354579:+ | 265.6172337 | 121.8368001 | 0.458693129 | 0.000570333 | 0.028898064 |
| ENSG00000248773 | sense intronic | chr3:140972744-140973255:+ | 50.29473749 | 17.72147087 | 0.352352388 | 0.000576439 | 0.029062157 |
| ENSG00000260401 | sense overlapping | chr11:73238975-73242335:+ | 27.72954519 | 124.1572985 | 4.477437247 | 0.000581529 | 0.029148611 |
| ENSG00000271840 | lincRNA | chr1:22100613-22101360:+ | 48.66973438 | 124.4617813 | 2.557272664 | 0.000621243 | 0.030336328 |
| MSTRG.152898 | antisense | chr6:73654143-73654484:+ | 1.119466813 | 8.412693929 | 7.514911415 | 0.000620241 | 0.030336328 |
| MSTRG.167539 | linc | chr7:148884475-148888236:+ | 45.57453179 | 15.7601021 | 0.345809413 | 0.000601836 | 0.029914325 |
| MSTRG.5497 | antisense | chr1:67754737-67829642:+ | 103.0979901 | 30.79160239 | 0.29866346 | 0.00061784 | 0.030336328 |
| ENSG00000272477 | sense intronic | chr3:18408680-18409635:- | 165.8223051 | 78.88452095 | 0.475717189 | 0.000600127 | 0.029884698 |
| ENSG00000233452 | antisense | chr6:146841901-147204614:- | 175.9474664 | 81.48568368 | 0.463125076 | 0.000606445 | 0.030014957 |
| MSTRG.71335 | linc | chr17:42195078-42198266:- | 23.89566697 | 72.24354605 | 3.02329063 | 0.00059416 | 0.029660457 |
| ENSG00000224184 | lincRNA | chr2:11848622-12578348:+ | 18.21105818 | 2.440072975 | 0.133988533 | 0.000616703 | 0.030336328 |
| MSTRG.134041 | antisense | chr4:143654875-143693497:+ | 59.19220703 | 19.95252667 | 0.337080296 | 0.000599273 | 0.029878893 |
| ENSG00000272341 | lincRNA | chr6:16764346-16766883:+ | 110.2633566 | 47.48949336 | 0.430691526 | 0.000615164 | 0.030335391 |
| MSTRG.86060 | linc | chr2:9573475-9576297:- | 2.325516398 | 13.77409052 | 5.923024464 | 0.000614097 | 0.030319644 |
| MSTRG.167222 | antisense | chr7:143485794-143496889:+ | 241.3184812 | 77.001402 | 0.31908622 | 0.000640329 | 0.030826281 |
| MSTRG.156726 | antisense | chr6:136938685-136980569:+ | 96.83952622 | 20.97046245 | 0.216548586 | 0.000640015 | 0.030826281 |
| MSTRG.65392 | antisense | chr16:58118438-58122283:- | 150.6306455 | 67.52092222 | 0.448254882 | 0.000655509 | 0.031085498 |
| MSTRG.116694 | linc | chr3:52251062-52260792:+ | 39.94026219 | 106.6772895 | 2.670921111 | 0.000654698 | 0.031085498 |
| ENSG00000279199 | TEC | chr17:40113215-40115442:- | 2.357236807 | 13.02838076 | 5.526971545 | 0.000652111 | 0.031025008 |
| MSTRG.133801 | antisense | chr4:139033397-139042812:- | 112.0967802 | 292.4743773 | 2.609123801 | 0.000664694 | 0.031293104 |
| ENSG00000274737 | sense intronic | chr12:47817451-47817966:- | 17.20285541 | 49.42357579 | 2.872986758 | 0.000683829 | 0.031883962 |
| ENSG00000255363 | lincRNA | chr11:76607853-76630427:- | 34.87126718 | 10.87358277 | 0.311820695 | 0.0006831 | 0.031883962 |
| ENSG00000263335 | antisense | chr16:15726674-15732993:+ | 134.4786069 | 310.673412 | 2.310206948 | 0.000680194 | 0.031853009 |
| MSTRG.63847 | antisense | chr16:11242743-11244679:- | 14.17400319 | 1.720430592 | 0.1213793 | 0.000705986 | 0.03248259 |
| MSTRG.110430 | antisense | chr22:29730163-29730684:- | 0.496573298 | 7.131206469 | 14.36083353 | 0.000711681 | 0.03260374 |
| ENSG00000273210 | antisense | chr21:37365477-37365932:- | 34.14890969 | 10.65595425 | 0.312043762 | 0.000740856 | 0.033666568 |
| MSTRG.21857 | linc | chr10:72328686-72366452:- | 34.40385288 | 85.10296643 | 2.473646389 | 0.000766062 | 0.034424176 |
| MSTRG.102630 | antisense | chr20:5608660-5613928:+ | 286.1128657 | 120.6495143 | 0.421685037 | 0.000787926 | 0.035020385 |
| MSTRG.50234 | linc | chr13:111037132-111049278:+ | 5.020310721 | 0.151254883 | 0.03012859 | 0.000793536 | 0.035115719 |
| MSTRG.11371 | linc | chr1:161634393-161637895:- | 3.248020881 | 41.32973495 | 12.72459028 | 0.000807454 | 0.035534528 |
| MSTRG.181523 | antisense | chr9:78695042-78853673:+ | 1.366055917 | 9.096667156 | 6.659073794 | 0.000812693 | 0.035613544 |
| ENSG00000276704 | sense intronic | chr13:97437268-97437630:+ | 57.3971468 | 21.18075996 | 0.36902113 | 0.000818362 | 0.035746022 |
| MSTRG.157406 | linc | chr6:144201668-144219922:- | 17.39643084 | 2.747305111 | 0.157923492 | 0.000827915 | 0.036008101 |
| ENSG00000277825 | lincRNA | chr19:16352462-16353182:- | 0.345730262 | 4.866631024 | 14.07638138 | 0.000824343 | 0.035891228 |
| ENSG00000261114 | sense intronic | chr16:56941028-56941726:+ | 49.38051329 | 17.47609479 | 0.353906706 | 0.000844527 | 0.036456784 |
| ENSG00000235257 | processed transcript | chr3:37745432-37861780:- | 73.85949033 | 27.46090574 | 0.371799286 | 0.000863488 | 0.036974831 |
| ENSG00000235527 | antisense | chr1:113924000-113929492:- | 75.03104895 | 29.97858718 | 0.399549088 | 0.000877331 | 0.037316473 |
| ENSG00000260244 | sense overlapping | chr4:155734448-155737062:+ | 295.677912 | 88.30143232 | 0.298640611 | 0.000894694 | 0.037738204 |
| MSTRG.180136 | linc | chr9:42428902-42437465:- | 82.45708354 | 12.2533011 | 0.148602165 | 0.00089174 | 0.037652797 |
| ENSG00000251661 | antisense | chr11:318640-325631:+ | 71.08314924 | 154.1325374 | 2.168341429 | 0.000908181 | 0.038079997 |
| ENSG00000237773 | antisense | chr7:17279834-17299357:- | 45.46273488 | 16.32672164 | 0.359123174 | 0.000925 | 0.038299171 |
| MSTRG.187021 | antisense | chrX:10018875-10037317:+ | 27.27077456 | 114.1703845 | 4.186547185 | 0.000930019 | 0.038334978 |
| ENSG00000273797 | sense intronic | chr14:69617122-69617648:+ | 236.3956288 | 116.9197653 | 0.494593601 | 0.000923184 | 0.038299171 |
| ENSG00000280402 | TEC | chr16:2578395-2579963:+ | 7.049130367 | 23.90982662 | 3.391883165 | 0.000965752 | 0.039422735 |
| MSTRG.65923 | antisense | chr16:67199589-67200633:- | 5.294476189 | 21.31962986 | 4.026768485 | 0.00099013 | 0.040094827 |
| MSTRG.166801 | antisense | chr7:140012852-140018111:- | 13.55510142 | 41.4356883 | 3.056833514 | 0.000986557 | 0.039990095 |
| MSTRG.23568 | antisense | chr10:97761089-97766900:+ | 61.58730841 | 136.0914746 | 2.209732462 | 0.00101101 | 0.04057552 |
| ENSG00000275496 | lincRNA | chr21:6228966-6267317:- | 7.545277182 | 0.844930155 | 0.111981327 | 0.001024781 | 0.040965983 |
| ENSG00000276255 | lincRNA | chr1:228073909-228076550:- | 15.62983977 | 45.05268583 | 2.882479058 | 0.001020572 | 0.040878348 |
| MSTRG.21858 | linc | chr10:72317843-72319731:- | 155.8093767 | 70.21260334 | 0.450631437 | 0.001047073 | 0.04148885 |
| MSTRG.145568 | linc | chr5:140578035-140629799:- | 234.1852201 | 627.9104115 | 2.681255509 | 0.001062021 | 0.041913825 |
| ENSG00000254810 | lincRNA | chr11:76654169-76656712:- | 140.0454896 | 48.10460222 | 0.343492692 | 0.001082008 | 0.042253562 |
| MSTRG.61364 | linc | chr15:84807049-84812566:+ | 75.98190705 | 182.4722975 | 2.401523001 | 0.001091103 | 0.042410058 |
| MSTRG.125588 | antisense | chr3:194468979-194472734:- | 157.5770625 | 73.58591415 | 0.466983665 | 0.001099477 | 0.042607379 |
| MSTRG.125600 | antisense | chr3:193644046-193683209:- | 4378.392572 | 1037.887846 | 0.23704769 | 0.001120998 | 0.043234696 |
| MSTRG.67234 | antisense | chr16:85759375-85774281:+ | 12.96830224 | 73.64333139 | 5.678718002 | 0.001118165 | 0.04320765 |
| ENSG00000273368 | sense intronic | chr18:8154558-8155070:+ | 14.36509686 | 3.3164871 | 0.230871197 | 0.001143387 | 0.043928186 |
| MSTRG.82703 | linc | chr19:35455075-35456852:+ | 2.387075724 | 20.16986129 | 8.449610996 | 0.001136505 | 0.043749492 |
| MSTRG.11326 | linc | chr1:161972044-161977537:+ | 19.03055451 | 51.47434748 | 2.704826465 | 0.001142937 | 0.043928186 |
| ENSG00000228063 | antisense | chr1:219086602-219173961:- | 102.720366 | 44.152475 | 0.429831753 | 0.001155808 | 0.044240484 |
| ENSG00000273176 | antisense | chr22:35298838-35299541:- | 23.75130791 | 6.734704998 | 0.283550911 | 0.001197707 | 0.045457913 |
| MSTRG.128662 | linc | chr4:38139377-38140379:+ | 21.89893551 | 57.87432027 | 2.642791484 | 0.001193205 | 0.045329496 |
| MSTRG.95207 | linc | chr2:146558329-146804977:- | 11.55973717 | 1.281691962 | 0.110875528 | 0.001209433 | 0.045658116 |
| MSTRG.168128 | linc | chr7:157424744-157426073:+ | 2.483212107 | 14.04863389 | 5.657444182 | 0.00120649 | 0.045620405 |
| MSTRG.43702 | linc | chr12:110712988-110714605:- | 6.18188091 | 24.68633843 | 3.993337754 | 0.001220493 | 0.045893022 |
| MSTRG.52953 | antisense | chr14:54681748-54729341:- | 1488.10352 | 473.1842937 | 0.317978076 | 0.001236303 | 0.046309723 |
| MSTRG.67209 | linc | chr16:85025712-85027615:- | 6.584856311 | 22.28752436 | 3.384663735 | 0.001248377 | 0.04651008 |
| MSTRG.81185 | linc | chr19:12191710-12192979:+ | 15.11116409 | 43.78430914 | 2.897480887 | 0.001256475 | 0.046725902 |
| MSTRG.23470 | linc | chr10:95110417-95113119:- | 15.27956529 | 3.720543873 | 0.243498019 | 0.001246978 | 0.046500688 |
| MSTRG.57603 | linc | chr15:35179487-35180820:- | 1.691976835 | 9.557164937 | 5.648519968 | 0.001278691 | 0.047464965 |
| ENSG00000267317 | antisense | chr19:1457670-1458580:- | 34.89885522 | 84.04361738 | 2.408205566 | 0.001289752 | 0.047744365 |
| MSTRG.185107 | antisense | chr9:120834046-120839950:- | 370.9553379 | 169.1275344 | 0.45592425 | 0.001301296 | 0.047996399 |
| MSTRG.39819 | antisense | chr12:57466810-57471270:- | 107.3580973 | 323.8518204 | 3.016557006 | 0.001297453 | 0.047941892 |
| MSTRG.112356 | linc | chr22:47449681-47451787:- | 2.273140853 | 11.4444579 | 5.034645295 | 0.001314288 | 0.048256033 |
| MSTRG.39289 | linc | chr12:53370289-53371245:- | 14.81242845 | 45.70998923 | 3.085921351 | 0.001313286 | 0.048256033 |
| ENSG00000269938 | sense intronic | chr12:123968023-123968579:- | 75.550322 | 32.05019281 | 0.424223113 | 0.001353307 | 0.049110401 |
| MSTRG.45555 | antisense | chr12:132762139-132770583:+ | 14.53306085 | 42.72778092 | 2.940040047 | 0.001345738 | 0.049011222 |
| MSTRG.172438 | antisense | chr8:73014128-73019595:+ | 242.4677254 | 99.8377879 | 0.411757019 | 0.001351837 | 0.049101005 |
| MSTRG.17861 | linc | chr10:3785565-3787696:+ | 988.7395685 | 258.4541214 | 0.261397571 | 0.001374198 | 0.049610835 |
| ENSG00000116652 | sense intronic | chr1:63547082-63550636:+ | 65.800999 | 26.63184864 | 0.404733196 | 0.001403032 | 0.050202071 |
| MSTRG.89332 | linc | chr2:49601869-49784790:+ | 103.7929784 | 36.98364422 | 0.356321254 | 0.001399077 | 0.050142865 |
| MSTRG.74632 | antisense | chr17:81552455-81555280:+ | 30.74176296 | 76.0864074 | 2.475017698 | 0.001519916 | 0.05230596 |
| MSTRG.19432 | linc | chr10:26332743-26380877:- | 191.0193731 | 63.26042161 | 0.331172805 | 0.001529084 | 0.052526643 |
| MSTRG.22781 | antisense | chr10:87864018-87864678:- | 3.260501355 | 13.26466741 | 4.068290722 | 0.001491748 | 0.051948455 |
| MSTRG.83025 | antisense | chr19:37781140-37782603:+ | 4.688192741 | 0.166314414 | 0.035475166 | 0.001519653 | 0.05230596 |
| MSTRG.162987 | linc | chr7:65081390-65082244:+ | 8.200444314 | 25.92852499 | 3.161843919 | 0.001499081 | 0.052138451 |
| MSTRG.12636 | linc | chr1:173926307-173930515:- | 67.54257019 | 159.6647181 | 2.363912384 | 0.001516939 | 0.05230596 |
| MSTRG.75812 | linc | chr18:11946335-11947764:- | 0.149628223 | 3.924999901 | 26.23168166 | 0.001491637 | 0.051948455 |
| MSTRG.111430 | antisense | chr22:37741031-37745984:- | 27.80060776 | 81.09007251 | 2.916845315 | 0.001486793 | 0.051865023 |
| MSTRG.67728 | linc | chr16:89968110-89969722:+ | 1.025626341 | 6.918012321 | 6.745158589 | 0.001516875 | 0.05230596 |
| ENSG00000179253 | antisense | chr20:61717506-61719748:- | 7.041213284 | 31.44547111 | 4.465916575 | 0.001515272 | 0.05230596 |
| MSTRG.151606 | antisense | chr6:43043514-43052909:+ | 2.398855991 | 11.22891607 | 4.680946296 | 0.00155558 | 0.052825678 |
| MSTRG.80249 | linc | chr19:5578684-5586448:- | 61.65880314 | 133.5407253 | 2.16580145 | 0.00157796 | 0.053435748 |
| ENSG00000272800 | lincRNA | chr2:183214319-183215400:+ | 21.7221597 | 6.564389374 | 0.302197823 | 0.001603286 | 0.053906807 |
| MSTRG.71753 | linc | chr17:45490885-45491658:+ | 7.909566035 | 29.69477494 | 3.754286241 | 0.001609286 | 0.053998648 |
| ENSG00000231563 | antisense | chr1:228407381-228409694:+ | 13.56478123 | 39.34640826 | 2.900629771 | 0.001600225 | 0.05387468 |
| ENSG00000205592 | processed transcript | chr12:40393395-40570832:+ | 9.463343141 | 1.673035714 | 0.176791192 | 0.001631366 | 0.054515585 |
| MSTRG.141046 | linc | chr5:70156735-70179224:- | 226.0867817 | 84.975654 | 0.375854145 | 0.001649371 | 0.054891007 |
| ENSG00000253819 | lincRNA | chr8:122670385-122694106:- | 31.3968557 | 9.491322256 | 0.302301681 | 0.001674473 | 0.055268543 |
| MSTRG.58962 | antisense | chr15:52187935-52194166:+ | 60.99162225 | 19.5156875 | 0.319973249 | 0.001681635 | 0.055373725 |
| MSTRG.37985 | antisense | chr12:31715566-31722338:+ | 1.50029714 | 11.0714058 | 7.379475373 | 0.001699427 | 0.055654113 |
| MSTRG.38119 | antisense | chr12:32691425-32696218:+ | 83.62706577 | 36.47997505 | 0.43622211 | 0.001711262 | 0.055936879 |
| ENSG00000283045 | sense intronic | chr17:44486153-44486815:- | 78.39100919 | 33.65908199 | 0.429374265 | 0.001745424 | 0.056600615 |
| MSTRG.164830 | antisense | chr7:100064921-100069223:- | 9.662692139 | 29.25010233 | 3.027117279 | 0.001755024 | 0.056800747 |
| ENSG00000251323 | lincRNA | chr11:78423982-78429836:- | 110.4787203 | 49.69027871 | 0.449772396 | 0.001769146 | 0.057050377 |
| MSTRG.47618 | linc | chr13:48567368-48570390:- | 3.649985679 | 17.88914756 | 4.901155548 | 0.001763306 | 0.056907324 |
| ENSG00000250334 | lincRNA | chr4:79492416-79576460:+ | 1539.89067 | 627.9818694 | 0.407809387 | 0.001787636 | 0.057463766 |
| MSTRG.4416 | linc | chr1:53363896-53366614:- | 14.5937506 | 41.81020315 | 2.864938856 | 0.00177814 | 0.057293727 |
| ENSG00000254165 | antisense | chr8:42537529-42538304:- | 91.72093148 | 42.02913231 | 0.458228363 | 0.001786413 | 0.057463766 |
| ENSG00000269974 | lincRNA | chr15:30648797-30649529:+ | 23.18221079 | 7.201094531 | 0.31063019 | 0.001812651 | 0.057900564 |
| MSTRG.94306 | antisense | chr2:131481076-131482024:- | 5.33342343 | 18.58242997 | 3.484146762 | 0.001801942 | 0.057649356 |
| ENSG00000249898 | antisense | chr8:6618475-6708209:- | 109.7656589 | 47.4469988 | 0.432257222 | 0.001808094 | 0.05780055 |
| ENSG00000273149 | antisense | chr13:45340039-45341183:+ | 28.09893125 | 69.70944895 | 2.480857664 | 0.001854144 | 0.058716982 |
| MSTRG.39530 | linc | chr12:56197580-56199781:- | 7.043407503 | 24.37687199 | 3.460948693 | 0.00185133 | 0.058673699 |
| ENSG00000255121 | lincRNA | chr11:118994824-118998004:- | 17.78484522 | 48.04425063 | 2.701415168 | 0.001865377 | 0.058934566 |
| MSTRG.146173 | linc | chr5:150993043-150993852:- | 6.141994979 | 0.677152643 | 0.110249625 | 0.001898619 | 0.059798389 |
| MSTRG.163416 | linc | chr7:76320039-76322513:+ | 2.618981427 | 14.15377995 | 5.40430711 | 0.001941628 | 0.060577007 |
| MSTRG.63757 | linc | chr16:16143637-16147933:+ | 27.25922765 | 68.39489322 | 2.509054698 | 0.001954372 | 0.060751909 |
| MSTRG.31440 | antisense | chr11:72759869-72814494:- | 663.5685075 | 1815.26439 | 2.735609617 | 0.001974786 | 0.061245559 |
| MSTRG.30565 | linc | chr11:65574460-65575811:+ | 35.3390897 | 82.242292 | 2.327232894 | 0.001994841 | 0.061529709 |
| MSTRG.91222 | linc | chr2:75239094-75416833:+ | 142.2015623 | 47.93808285 | 0.337113616 | 0.001989427 | 0.061515204 |
| ENSG00000157306 | processed transcript | chr14:23511760-23560778:+ | 15.77985588 | 43.1990495 | 2.737607353 | 0.002013352 | 0.061967513 |
| MSTRG.116307 | antisense | chr3:48458614-48467681:- | 121.795123 | 260.5154607 | 2.138964635 | 0.002085792 | 0.063493634 |
| MSTRG.136526 | linc | chr5:1561134-1564164:- | 64.69298497 | 24.21332289 | 0.374280502 | 0.002122742 | 0.064173566 |
| ENSG00000259278 | lincRNA | chr15:39019233-39024918:+ | 15.24585845 | 3.616507607 | 0.237212461 | 0.00213747 | 0.064352134 |
| MSTRG.166960 | antisense | chr7:140859529-140924283:- | 2503.36591 | 1248.854518 | 0.498870146 | 0.002134481 | 0.064328008 |
| ENSG00000280167 | TEC | chr11:94559018-94559374:+ | 12.52224488 | 2.941170165 | 0.234875631 | 0.002158258 | 0.064707842 |
| MSTRG.84405 | antisense | chr19:49551093-49565172:+ | 42.10746862 | 97.93373713 | 2.325804432 | 0.002175872 | 0.065139555 |
| MSTRG.53712 | antisense | chr14:64750701-64758924:+ | 250.8484002 | 645.3440775 | 2.572645777 | 0.002213397 | 0.065777168 |
| MSTRG.128652 | linc | chr4:37877914-37888331:+ | 36.3025812 | 119.7191532 | 3.297813798 | 0.002210286 | 0.06575088 |
| MSTRG.121315 | linc | chr3:129080251-129082986:- | 0.363714392 | 4.22617952 | 11.61950039 | 0.002209919 | 0.06575088 |
| MSTRG.73566 | linc | chr17:73281285-73282773:- | 6.142501573 | 27.32558203 | 4.448608063 | 0.002233584 | 0.066182985 |
| ENSG00000253364 | lincRNA | chr14:105644496-105649057:- | 13.03136673 | 40.94990707 | 3.142410762 | 0.002290554 | 0.067134915 |
| MSTRG.21886 | antisense | chr10:71803877-71806538:- | 50.14772468 | 133.9709184 | 2.671525364 | 0.002301028 | 0.067344516 |
| MSTRG.71436 | antisense | chr17:43388388-43391828:+ | 74.04637444 | 150.6423652 | 2.034432697 | 0.002288175 | 0.067127272 |
| MSTRG.98947 | antisense | chr2:199951979-199954510:- | 54.02071016 | 21.74604517 | 0.402550154 | 0.002356257 | 0.068076169 |
| MSTRG.16468 | linc | chr1:235012019-235013789:+ | 87.98507427 | 37.27813879 | 0.423687075 | 0.002350321 | 0.068050187 |
| MSTRG.56206 | linc | chr14:102732874-102743845:+ | 144.0359166 | 316.2341469 | 2.195522856 | 0.002288082 | 0.067127272 |
| ENSG00000281772 | TEC | chr2:148062154-148062577:+ | 18.68531536 | 3.516671276 | 0.18820508 | 0.002348919 | 0.068050187 |
| ENSG00000267226 | antisense | chr18:58670009-58671877:- | 11.71306416 | 2.68092628 | 0.228883428 | 0.002348797 | 0.068050187 |
| ENSG00000277245 | antisense | chr15:56447120-56447697:+ | 5.537765768 | 0.523977863 | 0.094619001 | 0.00234463 | 0.068031184 |
| MSTRG.3573 | linc | chr1:44200585-44206663:- | 34.81129979 | 11.09278521 | 0.318654726 | 0.00234104 | 0.068024399 |
| MSTRG.54756 | linc | chr14:77503115-77505323:- | 2.758344019 | 11.78404061 | 4.272143187 | 0.002335031 | 0.06789849 |
| ENSG00000280145 | lincRNA | chr21:6630182-6670695:- | 23.739383 | 5.50070308 | 0.231712133 | 0.00239358 | 0.068813895 |
| MSTRG.80516 | linc | chr19:7947506-7948477:- | 10.92216308 | 2.511501846 | 0.229945463 | 0.002410523 | 0.068955916 |
| MSTRG.154361 | linc | chr6:105751245-105758200:- | 0.902315972 | 16.98479853 | 18.82355966 | 0.002409239 | 0.068955916 |
| MSTRG.99092 | linc | chr2:202309238-202330014:+ | 48.77071906 | 115.7282572 | 2.372904468 | 0.002431529 | 0.069345521 |
| MSTRG.108602 | linc | chr21:42446035-42456563:- | 109.7237551 | 43.68989117 | 0.398180787 | 0.002445558 | 0.069419246 |
| MSTRG.122910 | antisense | chr3:151229627-151231683:- | 28.46124607 | 80.8036248 | 2.839075443 | 0.002504123 | 0.070590436 |
| ENSG00000245648 | antisense | chr12:10363769-10398506:+ | 14.47316636 | 3.26951791 | 0.225902047 | 0.002500782 | 0.070590436 |
| MSTRG.85048 | linc | chr19:56161863-56163526:+ | 4.151441872 | 15.01340736 | 3.616432031 | 0.00249698 | 0.070533154 |
| MSTRG.23644 | antisense | chr10:97346175-97435046:- | 11.55592728 | 37.76266996 | 3.26781824 | 0.002528634 | 0.070752177 |
| ENSG00000272983 | lincRNA | chr10:38137337-38144399:+ | 29.84056495 | 10.67505554 | 0.357736375 | 0.0025767 | 0.071538725 |
| MSTRG.187329 | antisense | chrX:13387468-13423459:- | 0.469157933 | 4.953187118 | 10.55761136 | 0.002574418 | 0.071538725 |
| MSTRG.98106 | antisense | chr2:190694763-190700937:+ | 31.29184758 | 93.77643204 | 2.996832699 | 0.002563849 | 0.071426556 |
| MSTRG.16534 | linc | chr1:235654423-235660031:- | 60.65758136 | 175.9653728 | 2.900962565 | 0.002572727 | 0.071538725 |
| MSTRG.63013 | linc | chr16:3037243-3046951:- | 46.58330491 | 154.0090311 | 3.306099285 | 0.00261855 | 0.072353736 |
| ENSG00000225963 | antisense | chr2:230121370-230174223:+ | 1.28835117 | 7.839624457 | 6.085005887 | 0.002627469 | 0.072501327 |
| MSTRG.165030 | antisense | chr7:100272407-100299834:- | 39.4828603 | 89.57655051 | 2.268745218 | 0.002613673 | 0.072268247 |
| MSTRG.21514 | linc | chr10:68793087-68794963:+ | 8.845593488 | 26.22862697 | 2.96516305 | 0.002643842 | 0.072853927 |
| MSTRG.81524 | antisense | chr19:17735058-17751646:- | 46.13786398 | 105.1483555 | 2.279003542 | 0.002651536 | 0.072920941 |
| MSTRG.108059 | antisense | chr21:34517762-34538256:+ | 15.3609713 | 46.65727079 | 3.037390662 | 0.002697947 | 0.073743277 |
| MSTRG.22946 | linc | chr10:89027822-89033111:+ | 62.01481834 | 149.6873399 | 2.413735038 | 0.002757466 | 0.07461542 |
| MSTRG.79330 | linc | chr18:77162446-77174076:+ | 54.51213486 | 21.83056698 | 0.400471694 | 0.002754106 | 0.074594777 |
| MSTRG.106640 | linc | chr21:8251249-8434344:+ | 46545.70124 | 9852.860342 | 0.211681424 | 0.002747811 | 0.074553219 |
| MSTRG.64433 | linc | chr16:28076685-28097390:- | 202.0066167 | 540.9081425 | 2.677675372 | 0.002746411 | 0.074553219 |
| MSTRG.8711 | antisense | chr1:110572094-110618959:+ | 34.9927033 | 8.465006942 | 0.241907773 | 0.002740127 | 0.074494324 |
| ENSG00000253174 | antisense | chr8:41540381-41545044:- | 4.613079555 | 0.350446241 | 0.075967959 | 0.002840622 | 0.075781712 |
| ENSG00000270419 | lincRNA | chr6:163413065-163413960:- | 50.67869723 | 20.459135 | 0.40370286 | 0.002838071 | 0.075781712 |
| MSTRG.55290 | linc | chr14:92750372-92753656:+ | 309.461424 | 153.2006562 | 0.495055746 | 0.002873654 | 0.076382654 |
| ENSG00000279845 | TEC | chr4:146180851-146181316:+ | 55.33715497 | 22.57566713 | 0.407965808 | 0.002900723 | 0.076871027 |
| MSTRG.107981 | antisense | chr21:33438989-33440651:+ | 4.138548952 | 22.84878946 | 5.520966341 | 0.002936957 | 0.077451651 |
| ENSG00000265791 | sense intronic | chr17:30781493-30782221:- | 8.642452884 | 25.92963179 | 3.00026302 | 0.002957803 | 0.077651896 |
| MSTRG.184314 | antisense | chr9:112483558-112484577:+ | 2.331646655 | 10.22765137 | 4.386449956 | 0.002977486 | 0.078017057 |
| ENSG00000273402 | antisense | chr8:237045-237669:+ | 32.29483239 | 11.73307199 | 0.363311128 | 0.00300351 | 0.078495983 |
| MSTRG.130693 | linc | chr4:78727269-78754495:+ | 110.1330852 | 422.032929 | 3.832026755 | 0.003024939 | 0.078903422 |
| ENSG00000267416 | lincRNA | chr17:60079309-60088695:+ | 4.21161942 | 18.1054044 | 4.298917495 | 0.003041071 | 0.079120548 |
| ENSG00000273855 | sense intronic | chr15:40285468-40285909:- | 8.969452745 | 27.03586112 | 3.014215236 | 0.003092822 | 0.079953779 |
| MSTRG.150535 | antisense | chr6:32473789-32531328:+ | 42.16398955 | 7.89552355 | 0.187257507 | 0.003090942 | 0.079953779 |
| MSTRG.171406 | linc | chr8:58396167-58396889:- | 24.09754542 | 4.731451902 | 0.196345803 | 0.003171611 | 0.081233974 |
| MSTRG.163338 | linc | chr7:74280626-74289295:- | 19.95366274 | 4.078982883 | 0.204422764 | 0.003168194 | 0.081228807 |
| MSTRG.153609 | linc | chr6:89357830-89362898:- | 10.33848858 | 31.18570748 | 3.016466792 | 0.003166766 | 0.081228807 |
| MSTRG.184532 | antisense | chr9:114902988-114904855:+ | 1.07935257 | 8.040721535 | 7.449578347 | 0.003263505 | 0.08288608 |
| MSTRG.60140 | linc | chr15:69971436-69975793:- | 1.936692891 | 9.848230191 | 5.085075821 | 0.003319561 | 0.083628597 |
| MSTRG.134161 | antisense | chr4:146277703-146337828:+ | 31.37964667 | 8.660879005 | 0.276003076 | 0.003315388 | 0.083617675 |
| MSTRG.139685 | linc | chr5:62762634-62764058:- | 9.62446926 | 1.114562975 | 0.115805136 | 0.003348317 | 0.083987654 |
| MSTRG.81999 | linc | chr19:25525711-25549142:+ | 3.608835488 | 0.162753174 | 0.04509853 | 0.003313496 | 0.083617675 |
| ENSG00000275426 | sense intronic | chr4:149738-150317:+ | 39.10757921 | 15.56608078 | 0.398032328 | 0.003345147 | 0.083960079 |
| MSTRG.96089 | linc | chr2:159307052-159312559:- | 14.40675465 | 42.30565286 | 2.93651512 | 0.003312835 | 0.083617675 |
| MSTRG.163413 | linc | chr7:76319438-76325820:- | 29.13599367 | 70.85552662 | 2.431889827 | 0.003295285 | 0.083431836 |
| MSTRG.39793 | antisense | chr12:57398836-57429306:- | 20.51094741 | 50.37404267 | 2.455958843 | 0.003304212 | 0.083501397 |
| MSTRG.31016 | antisense | chr11:70384357-70385607:- | 51.82593199 | 19.83246083 | 0.382674466 | 0.003432351 | 0.085303703 |
| MSTRG.83071 | antisense | chr19:38734269-38735428:+ | 0.441742567 | 4.628931185 | 10.47879813 | 0.003427123 | 0.085303703 |
| MSTRG.13472 | linc | chr1:194220525-194230636:- | 288.4623672 | 123.7034974 | 0.428837559 | 0.003465483 | 0.085691993 |
| ENSG00000279095 | TEC | chr19:44664131-44666158:+ | 13.71642777 | 35.86063945 | 2.614429941 | 0.003457667 | 0.085634221 |
| MSTRG.140177 | antisense | chr5:67163497-67166168:- | 60.96617805 | 141.9701442 | 2.328670564 | 0.00345074 | 0.08560326 |
| ENSG00000246448 | antisense | chr4:143700257-143865072:+ | 142.6422435 | 55.18391859 | 0.386869396 | 0.003508001 | 0.086494414 |
| ENSG00000248571 | antisense | chr4:152666368-152670107:+ | 8.252644619 | 1.391889001 | 0.168659753 | 0.003547324 | 0.086987762 |
| MSTRG.16365 | linc | chr1:234610266-234616823:+ | 179.3100136 | 81.18358027 | 0.452755419 | 0.003567103 | 0.087261619 |
| MSTRG.166966 | linc | chr7:140924883-140927109:+ | 93.91472517 | 16.40883375 | 0.174720564 | 0.003559312 | 0.087123616 |
| MSTRG.1963 | linc | chr1:21210733-21217072:- | 29.20838246 | 82.10328553 | 2.810949413 | 0.003611269 | 0.088235555 |
| MSTRG.80439 | antisense | chr19:7533363-7534565:- | 11.89369368 | 34.70087943 | 2.917586443 | 0.003606899 | 0.088181934 |
| MSTRG.185500 | linc | chr9:128665373-128680368:- | 25.73048616 | 61.56684569 | 2.392758742 | 0.003629532 | 0.088574993 |
| MSTRG.73936 | antisense | chr17:75677207-75682733:+ | 3.71054678 | 14.31255714 | 3.85726363 | 0.003651262 | 0.088891237 |
| MSTRG.31105 | linc | chr11:71657017-71659915:+ | 0.520719319 | 4.88341594 | 9.378211571 | 0.00367066 | 0.089155775 |
| MSTRG.105005 | antisense | chr20:45930016-45932903:- | 6.827821501 | 21.93994484 | 3.213315526 | 0.003746787 | 0.08992066 |
| ENSG00000247775 | antisense | chr4:89836408-89841978:+ | 6.610761448 | 1.008883383 | 0.152612281 | 0.003693893 | 0.089648868 |
| ENSG00000261101 | sense overlapping | chrX:101627868-101628523:+ | 69.30606658 | 31.30094172 | 0.451633504 | 0.003707165 | 0.089690499 |
| MSTRG.66237 | linc | chr16:69944860-69947735:+ | 35.53043578 | 77.10564854 | 2.170129548 | 0.003744826 | 0.08992066 |
| MSTRG.195300 | linc | chrY:8002712-8007593:- | 57.82626163 | 0.453764648 | 0.007847034 | 0.003763877 | 0.089999738 |
| MSTRG.84398 | linc | chr19:49549673-49554377:- | 9.305780633 | 32.26129091 | 3.46680114 | 0.003790386 | 0.090219206 |
| ENSG00000268729 | antisense | chr19:55312029-55312495:- | 0.337396538 | 3.884289864 | 11.51253621 | 0.003744743 | 0.08992066 |
| MSTRG.37600 | linc | chr12:25301294-25326488:- | 12.12747195 | 2.688044373 | 0.221649193 | 0.003784035 | 0.090180083 |
| MSTRG.189620 | antisense | chrX:49256161-49256862:+ | 1.339863808 | 13.36473988 | 9.974700266 | 0.003808856 | 0.090552484 |
| MSTRG.62902 | antisense | chr16:2785942-2791742:+ | 0.673695565 | 5.383419915 | 7.990879258 | 0.003744422 | 0.08992066 |
| ENSG00000258504 | lincRNA | chr14:100291117-100294656:+ | 5.640991345 | 18.0593281 | 3.20144581 | 0.003741762 | 0.08992066 |
| ENSG00000233214 | lincRNA | chr19:35424062-35424652:- | 4.142778666 | 16.07113468 | 3.879312889 | 0.003850708 | 0.091119924 |
| ENSG00000236226 | antisense | chr7:103030104-103031354:+ | 0.169784186 | 3.106843866 | 18.29878238 | 0.00389057 | 0.091353273 |
| ENSG00000279513 | TEC | chr1:117493515-117495006:+ | 58.79372532 | 26.23142048 | 0.446160204 | 0.004044068 | 0.093940562 |
| MSTRG.150962 | antisense | chr6:35732133-35735076:+ | 32.27952205 | 86.01293486 | 2.664628513 | 0.004059686 | 0.094249354 |
| MSTRG.122492 | linc | chr3:150478705-150482413:- | 6.428938367 | 0.511860039 | 0.079618128 | 0.004261475 | 0.096794137 |
| MSTRG.85257 | linc | chr19:57522826-57524670:+ | 16.45648235 | 3.997944238 | 0.24294039 | 0.004254888 | 0.096785562 |
| MSTRG.20446 | linc | chr10:12270186-12271320:+ | 2.958672423 | 12.48200761 | 4.218786614 | 0.004302722 | 0.097164812 |
| MSTRG.24156 | antisense | chr10:104036202-104039518:+ | 15.29156172 | 42.10296579 | 2.753346359 | 0.004359483 | 0.09789569 |
| ENSG00000274370 | lincRNA | chr17:83098377-83098987:+ | 28.45184383 | 80.24579209 | 2.820407443 | 0.004353976 | 0.097831722 |
| ENSG00000271761 | lincRNA | chr6:57902609-57903148:- | 6.814896723 | 1.155678938 | 0.169581284 | 0.004351035 | 0.097819851 |
| ENSG00000248455 | lincRNA | chr5:17404019-17441694:+ | 2.046251548 | 8.869798234 | 4.334656823 | 0.004347894 | 0.097819851 |
| MSTRG.102592 | linc | chr20:5504620-5506455:- | 2.382487329 | 10.50074952 | 4.407473396 | 0.004409741 | 0.098647586 |
| MSTRG.110552 | antisense | chr22:30655474-30656107:- | 28.47896728 | 79.64106081 | 2.796486966 | 0.00440188 | 0.098580219 |
| ENSG00000258418 | lincRNA | chr14:40386252-40386794:+ | 9.340942579 | 1.440644265 | 0.154229003 | 0.004434202 | 0.098882165 |
| MSTRG.72535 | antisense | chr17:57909239-57933850:- | 80.03001362 | 175.9344042 | 2.198355295 | 0.004479329 | 0.099381275 |
| ENSG00000277496 | antisense | chr20:62648961-62650767:- | 59.2843013 | 124.6969401 | 2.103372012 | 0.004468845 | 0.099311755 |
| MSTRG.84925 | linc | chr19:55224421-55224934:- | 6.620818267 | 20.15140821 | 3.043643156 | 0.004519997 | 0.100064492 |
| ENSG00000228719 | lincRNA | chr22:36445395-36454944:- | 9.193960632 | 32.92710259 | 3.581383901 | 0.004569868 | 0.100575408 |
| ENSG00000227355 | antisense | chr9:121369906-121463237:+ | 71.69498258 | 31.79176888 | 0.443430875 | 0.004588593 | 0.10074096 |
| MSTRG.12928 | linc | chr1:185386731-185389229:- | 0.363714392 | 3.778515542 | 10.38868856 | 0.004678557 | 0.101795647 |
| MSTRG.6410 | linc | chr1:77684677-77686388:- | 0.606006896 | 4.895008347 | 8.077479609 | 0.004784829 | 0.103111664 |
| MSTRG.147315 | antisense | chr5:172114691-172128814:+ | 1.921131398 | 8.725500561 | 4.541855164 | 0.004797997 | 0.103233768 |
| ENSG00000272701 | antisense | chr7:130486042-130491033:- | 11.34438379 | 2.597722683 | 0.228987553 | 0.0048273 | 0.103531541 |
| MSTRG.13303 | linc | chr1:192106198-192157884:+ | 1066.475306 | 442.4597424 | 0.414880438 | 0.004852413 | 0.103772956 |
| MSTRG.47717 | linc | chr13:49639058-49671747:+ | 190.6008364 | 474.4947045 | 2.489468113 | 0.004866123 | 0.103903708 |
| MSTRG.112464 | linc | chr22:49893250-49898379:- | 8.986043114 | 25.32429968 | 2.818181413 | 0.004898109 | 0.104223016 |
| MSTRG.76257 | linc | chr18:21637627-21648684:- | 50.50050539 | 118.8384147 | 2.353212384 | 0.004921593 | 0.104514901 |
| MSTRG.140214 | linc | chr5:71567980-71580129:+ | 12.18480803 | 31.32337886 | 2.570691207 | 0.004991771 | 0.105167073 |
| MSTRG.122263 | linc | chr3:143177787-143179103:+ | 23.62322095 | 6.226048515 | 0.263556292 | 0.004981403 | 0.105035147 |
| MSTRG.164389 | linc | chr7:76456657-76457473:- | 0.149628223 | 3.051126624 | 20.39138454 | 0.004966002 | 0.105006196 |
| ENSG00000269553 | sense intronic | chr19:35330843-35331920:+ | 2.372931802 | 10.96790492 | 4.622090239 | 0.004980867 | 0.105035147 |
| MSTRG.179970 | linc | chr9:41452326-41453266:+ | 47.43717501 | 20.37577269 | 0.429531748 | 0.00506379 | 0.105968375 |
| MSTRG.68116 | linc | chr17:1629558-1629982:- | 0.81931054 | 7.63090222 | 9.313809417 | 0.005019176 | 0.105574306 |
| MSTRG.80548 | antisense | chr19:8233377-8234022:+ | 0.14236882 | 3.020063687 | 21.21295718 | 0.005060658 | 0.105957531 |
| ENSG00000238057 | antisense | chr2:144518097-144521477:+ | 84.06125124 | 39.47776221 | 0.469630913 | 0.005049536 | 0.105888745 |
| MSTRG.180491 | linc | chr9:63814492-63817650:- | 31.94746836 | 72.96017555 | 2.283754528 | 0.005097791 | 0.106405235 |
| MSTRG.181983 | linc | chr9:85533984-85540704:- | 27.05464819 | 60.54391512 | 2.237837827 | 0.00509172 | 0.106356785 |
| MSTRG.38283 | antisense | chr12:39849086-39863560:- | 143.7641982 | 67.97172626 | 0.472800093 | 0.005048975 | 0.105888745 |
| ENSG00000273951 | sense intronic | chr20:41485571-41486225:- | 66.26309888 | 30.64797142 | 0.46251944 | 0.005171745 | 0.107112077 |
| MSTRG.150009 | linc | chr6:26670829-26686130:+ | 111.194439 | 48.37519083 | 0.435050451 | 0.005164264 | 0.10710705 |
| ENSG00000264007 | sense intronic | chr17:29621617-29622254:- | 2.270559922 | 9.499947953 | 4.183967074 | 0.005219215 | 0.10777426 |
| MSTRG.149315 | linc | chr6:14241862-14253442:+ | 9.693246549 | 2.389384422 | 0.246499912 | 0.005248219 | 0.10806652 |
| MSTRG.137019 | antisense | chr5:10492129-10498784:+ | 18.69001282 | 86.07128358 | 4.605201955 | 0.005244937 | 0.10806652 |
| MSTRG.163167 | linc | chr7:66964205-66970222:+ | 1.899169273 | 9.699663224 | 5.107318953 | 0.005291527 | 0.108823968 |
| MSTRG.55150 | antisense | chr14:81064675-81161621:- | 113.8448082 | 47.19457821 | 0.414551871 | 0.005338252 | 0.109298335 |
| MSTRG.80004 | antisense | chr19:3159496-3169946:- | 13.62931486 | 51.75560152 | 3.79737368 | 0.005365248 | 0.109599442 |
| MSTRG.54308 | linc | chr14:73224732-73232041:+ | 57.0229218 | 120.1427457 | 2.106920198 | 0.005404965 | 0.110040313 |
| MSTRG.77770 | antisense | chr18:49816683-49821978:+ | 30.66560851 | 68.38232492 | 2.229935366 | 0.005465627 | 0.110931442 |
| MSTRG.82539 | linc | chr19:34232208-34239188:+ | 90.96941408 | 197.1384261 | 2.167084707 | 0.00550928 | 0.111430565 |
| MSTRG.43191 | linc | chr12:105559455-105562021:+ | 4.492710502 | 0.468824179 | 0.10435219 | 0.005526593 | 0.111563976 |
| MSTRG.148547 | antisense | chr6:4130950-4133519:- | 55.80537661 | 21.53459888 | 0.385887529 | 0.005506661 | 0.111430565 |
| MSTRG.103212 | linc | chr20:19207395-19212208:- | 45.46180988 | 7.605014001 | 0.167283573 | 0.005500928 | 0.111378689 |
| MSTRG.55857 | linc | chr14:99267637-99276524:+ | 0.479765359 | 4.270333903 | 8.900880039 | 0.005564193 | 0.111988358 |
| ENSG00000235058 | antisense | chr3:50341106-50345697:+ | 1.853395663 | 7.986993423 | 4.309383896 | 0.005556027 | 0.111967473 |
| ENSG00000274827 | lincRNA | chr14:19344578-19384587:- | 4.359609966 | 0.510838229 | 0.117175214 | 0.005605266 | 0.112503534 |
| MSTRG.45583 | antisense | chr12:133125355-133125711:- | 4.627151325 | 0.50981642 | 0.110179327 | 0.005671201 | 0.113410037 |
| MSTRG.15800 | linc | chr1:228109936-228116161:+ | 72.05918374 | 32.20871125 | 0.446975799 | 0.005669476 | 0.113410037 |
| MSTRG.161008 | linc | chr7:26024322-26029736:- | 3.244807128 | 0.176914618 | 0.054522383 | 0.005748165 | 0.114497225 |
| MSTRG.64596 | linc | chr16:30804550-30815395:+ | 3.755169057 | 12.93391733 | 3.444296949 | 0.005768114 | 0.114781787 |
| MSTRG.179556 | linc | chr9:36164910-36167780:+ | 47.39102806 | 128.380051 | 2.70895265 | 0.005912039 | 0.116615324 |
| ENSG00000273424 | lincRNA | chr22:41430934-41431375:+ | 2.699587412 | 10.05083481 | 3.723100338 | 0.005936565 | 0.116985257 |
| MSTRG.38009 | antisense | chr12:31895068-31959370:- | 208.4537867 | 499.9610861 | 2.3984265 | 0.005966513 | 0.11707482 |
| MSTRG.159155 | linc | chr7:2852317-2902240:- | 14.48793964 | 38.00435399 | 2.623171751 | 0.005957356 | 0.11707482 |
| MSTRG.11551 | linc | chr1:163931955-164053424:+ | 34.96973523 | 11.97309959 | 0.342384622 | 0.006056382 | 0.118083426 |
| MSTRG.64641 | antisense | chr16:31086753-31090031:+ | 1.65964694 | 13.67255178 | 8.238229138 | 0.006048052 | 0.118029671 |
| MSTRG.2072 | linc | chr1:25218420-25222026:- | 175.0336573 | 87.0385078 | 0.497267264 | 0.006117352 | 0.118474371 |
| MSTRG.141026 | linc | chr5:81818225-81845739:+ | 4.152007912 | 18.14405865 | 4.369947994 | 0.006176236 | 0.119173492 |
| ENSG00000166770 | lincRNA | chr19:56477250-56500666:+ | 41.2206627 | 17.40790773 | 0.422310234 | 0.006260521 | 0.120270247 |
| MSTRG.10585 | linc | chr1:156180278-156191023:+ | 187.8804582 | 553.8890856 | 2.948093116 | 0.006310234 | 0.120938699 |
| ENSG00000274213 | lincRNA | chr17:56914186-56914533:+ | 25.78859448 | 73.35660485 | 2.844536755 | 0.006307172 | 0.120938699 |
| MSTRG.34782 | antisense | chr11:123131666-123136482:+ | 0.583994086 | 5.551365226 | 9.505858645 | 0.006344941 | 0.121322635 |
| ENSG00000214407 | lincRNA | chr3:101940859-101997926:+ | 15.10117317 | 4.675483557 | 0.309610618 | 0.006399268 | 0.122010509 |
| MSTRG.43517 | antisense | chr12:108636067-108641313:- | 4.673964003 | 15.49099541 | 3.314316371 | 0.006450044 | 0.122535492 |
| ENSG00000228277 | lincRNA | chr4:73710302-73714527:+ | 3.362704795 | 0.176914618 | 0.052610808 | 0.006484413 | 0.122910607 |
