# Supplementary material for: LncRNA LYPLAL1-DT screening from type 2 diabetes with macrovascular complication contributes protective effects on human umbilical vein endothelial cells via regulating the miR-204-5p/SIRT1 axis
Source: Cell Death Discov. 2022 May 4;8:245. doi: 10.1038/s41420-022-01019-z (PMC9068612; doi:10.1038/s41420-022-01019-z)
Supplement: Supplementary file 6 — supplementary table 4 [file 41420_2022_1019_MOESM6_ESM.docx]

Table S4. The differential expression of 798 mRNAs (DMC-mRNAs) identified between DMC patients and healthy control.

| **Gene** | **MC_normalize** | **C_normalize** | **FC** | **pval** | **padj** |
| --- | --- | --- | --- | --- | --- |
| ENSG00000125900 | 80.46468745 | 34.46199855 | 2.334881633 | 0.011518504 | 0.165980291 |
| ENSG00000167261 | 2110.556083 | 1004.48698 | 2.101128363 | 0.003397251 | 0.084847548 |
| ENSG00000119280 | 360.0565042 | 921.7288255 | 0.390631707 | 0.000511702 | 0.027113509 |
| ENSG00000187699 | 1705.68829 | 6001.458119 | 0.284212312 | 0.000168303 | 0.012836577 |
| ENSG00000138798 | 369.3250614 | 1050.17601 | 0.351679202 | 0.002399857 | 0.06884516 |
| ENSG00000165702 | 462.5341422 | 1082.751927 | 0.427183855 | 0.000549321 | 0.028120156 |
| ENSG00000156265 | 3286.210888 | 7128.402693 | 0.461002419 | 0.008193349 | 0.140074815 |
| ENSG00000143995 | 240.1530837 | 552.522602 | 0.434648434 | 0.007951772 | 0.138040711 |
| ENSG00000065534 | 672.9389717 | 1780.575218 | 0.377933471 | 0.003208642 | 0.081800192 |
| ENSG00000169860 | 174.2764048 | 429.5242954 | 0.405742834 | 0.008881358 | 0.14604368 |
| ENSG00000107438 | 765.270606 | 1665.820741 | 0.459395532 | 0.001351078 | 0.049101005 |
| ENSG00000101856 | 1167.678946 | 3749.770006 | 0.311400151 | 0.000327271 | 0.020222425 |
| ENSG00000168497 | 6118.226769 | 21735.2308 | 0.281488926 | 0.000151729 | 0.011988992 |
| ENSG00000088826 | 304.1232002 | 747.1960636 | 0.407019275 | 0.005816009 | 0.115338517 |
| ENSG00000113140 | 3211.294273 | 9247.315049 | 0.347267748 | 0.005414578 | 0.110180687 |
| ENSG00000003436 | 213.8526274 | 632.0733658 | 0.338335135 | 0.001017997 | 0.040815549 |
| ENSG00000101162 | 9797.530353 | 30459.94437 | 0.32165293 | 0.000881416 | 0.037372609 |
| ENSG00000182117 | 880.6976275 | 285.0718081 | 3.089388718 | 2.68256E-07 | 0.000123565 |
| ENSG00000175768 | 51.17173835 | 105.6972098 | 0.484135186 | 0.002530199 | 0.070752177 |
| ENSG00000177606 | 255.6786711 | 3268.508498 | 0.078224876 | 2.7072E-29 | 5.48682E-25 |
| ENSG00000123358 | 159.2204116 | 930.1322606 | 0.171180399 | 8.95901E-23 | 1.21051E-18 |
| ENSG00000153234 | 303.9781335 | 1480.435382 | 0.205330227 | 1.09945E-21 | 8.91324E-18 |
| ENSG00000168298 | 2434.435939 | 11196.65063 | 0.217425373 | 9.45694E-22 | 8.91324E-18 |
| ENSG00000113070 | 41.87679431 | 407.348072 | 0.102803468 | 1.54537E-20 | 1.04403E-16 |
| ENSG00000165633 | 1.007242041 | 49.97556123 | 0.020154692 | 5.71019E-17 | 3.30661E-13 |
| ENSG00000172179 | 0.343229032 | 34.26755492 | 0.010016152 | 2.2187E-15 | 8.99351E-12 |
| ENSG00000154640 | 130.2682358 | 484.6918577 | 0.268765059 | 4.55633E-13 | 1.23127E-09 |
| ENSG00000196154 | 6296.177506 | 1767.533144 | 3.562126983 | 4.46742E-13 | 1.23127E-09 |
| ENSG00000162772 | 56.4576449 | 289.6157633 | 0.19493982 | 7.61176E-13 | 1.81496E-09 |
| ENSG00000198918 | 5710.980057 | 1366.492065 | 4.17929983 | 1.72613E-12 | 3.68256E-09 |
| ENSG00000110848 | 704.0347605 | 3990.612114 | 0.176422749 | 3.72495E-12 | 7.19005E-09 |
| ENSG00000184357 | 342.6812405 | 1441.337207 | 0.237752303 | 5.12213E-11 | 9.0272E-08 |
| ENSG00000119508 | 44.53456636 | 332.9275191 | 0.133766552 | 7.13521E-11 | 1.11241E-07 |
| ENSG00000205220 | 417.9029417 | 96.9888142 | 4.308774627 | 8.38378E-11 | 1.25865E-07 |
| ENSG00000034510 | 10401.83445 | 3260.630083 | 3.190130186 | 1.46988E-10 | 2.05454E-07 |
| ENSG00000117984 | 1702.504928 | 561.2405066 | 3.033467664 | 2.98066E-10 | 3.86505E-07 |
| ENSG00000276368 | 127.1564756 | 541.0908395 | 0.235000237 | 4.52001E-10 | 5.55208E-07 |
| ENSG00000205189 | 528.0391619 | 1434.010033 | 0.36822557 | 5.8705E-10 | 6.99885E-07 |
| ENSG00000177700 | 325.6178208 | 85.71134071 | 3.799005104 | 8.90731E-10 | 9.48369E-07 |
| ENSG00000112245 | 1773.378937 | 4364.418945 | 0.406326468 | 1.23005E-09 | 1.2465E-06 |
| ENSG00000128791 | 236.1691707 | 705.1606007 | 0.334915437 | 1.7157E-09 | 1.65586E-06 |
| ENSG00000213741 | 3258.441399 | 1029.630959 | 3.164669216 | 1.81253E-09 | 1.70862E-06 |
| ENSG00000282608 | 77.38122119 | 8.152219824 | 9.492043009 | 2.01943E-09 | 1.81905E-06 |
| ENSG00000138778 | 116.3287522 | 368.2129603 | 0.315927913 | 4.06886E-09 | 3.50918E-06 |
| ENSG00000163660 | 8023.242556 | 20831.51498 | 0.385149259 | 5.68214E-09 | 4.60651E-06 |
| ENSG00000121621 | 35.00087851 | 138.0034403 | 0.253623232 | 1.18829E-08 | 9.26295E-06 |
| ENSG00000169442 | 3759.589336 | 1363.656076 | 2.756992325 | 1.53409E-08 | 1.15156E-05 |
| ENSG00000125812 | 288.2182226 | 774.1621359 | 0.372296977 | 1.71075E-08 | 1.26082E-05 |
| ENSG00000205927 | 48.58964541 | 1.587614605 | 30.60544117 | 3.33066E-08 | 2.25014E-05 |
| ENSG00000138182 | 497.2508839 | 1167.654105 | 0.42585461 | 4.2733E-08 | 2.70654E-05 |
| ENSG00000108846 | 550.0598476 | 1557.4474 | 0.353180369 | 4.21036E-08 | 2.70654E-05 |
| ENSG00000274736 | 12.95364625 | 0.14236882 | 90.98653927 | 4.18476E-08 | 2.70654E-05 |
| ENSG00000116678 | 279.3381685 | 738.3923998 | 0.378305855 | 4.8442E-08 | 2.93074E-05 |
| ENSG00000151575 | 66.53660233 | 208.355244 | 0.319342106 | 5.91443E-08 | 3.42488E-05 |
| ENSG00000197956 | 5905.755531 | 2074.530953 | 2.846790751 | 6.37231E-08 | 3.63805E-05 |
| ENSG00000176887 | 13.20116939 | 0.306633149 | 43.05199689 | 8.49065E-08 | 4.71464E-05 |
| ENSG00000161835 | 79.09312214 | 327.7632198 | 0.241311768 | 9.99109E-08 | 5.39985E-05 |
| ENSG00000196914 | 1315.457045 | 3218.309011 | 0.408741684 | 9.84455E-08 | 5.39255E-05 |
| ENSG00000133773 | 662.0524622 | 1520.730879 | 0.435351495 | 1.16394E-07 | 6.12729E-05 |
| ENSG00000068796 | 4077.298637 | 11525.83034 | 0.353753137 | 1.25098E-07 | 6.50108E-05 |
| ENSG00000137812 | 103.9543104 | 290.7923234 | 0.357486433 | 1.4054E-07 | 7.21115E-05 |
| ENSG00000196787 | 568.8663928 | 1357.789768 | 0.418965002 | 1.53419E-07 | 7.77353E-05 |
| ENSG00000123689 | 424.6858177 | 2424.020553 | 0.175198934 | 1.82562E-07 | 9.13598E-05 |
| ENSG00000232810 | 442.1210519 | 157.9255365 | 2.799553902 | 1.88362E-07 | 9.3113E-05 |
| ENSG00000170906 | 347.3383394 | 118.551654 | 2.929848111 | 2.0257E-07 | 9.89299E-05 |
| ENSG00000206560 | 2181.383012 | 4752.812607 | 0.458966762 | 2.46142E-07 | 0.000117381 |
| ENSG00000155090 | 1016.006657 | 3496.79183 | 0.290553944 | 2.55509E-07 | 0.000120431 |
| ENSG00000188186 | 948.1688516 | 378.3784699 | 2.505874216 | 2.65792E-07 | 0.000123565 |
| ENSG00000213654 | 5165.518426 | 2210.366671 | 2.336950921 | 2.7669E-07 | 0.000126018 |
| ENSG00000138166 | 199.6321721 | 493.5703832 | 0.40446546 | 3.09716E-07 | 0.000134993 |
| ENSG00000103056 | 548.9934546 | 105.1194071 | 5.22256993 | 3.09704E-07 | 0.000134993 |
| ENSG00000151748 | 344.6948124 | 851.0664197 | 0.405015172 | 3.08915E-07 | 0.000134993 |
| ENSG00000066279 | 83.95859378 | 260.5143 | 0.322280173 | 3.41592E-07 | 0.000141113 |
| ENSG00000180628 | 3142.832137 | 6979.506594 | 0.450294314 | 3.32062E-07 | 0.00014021 |
| ENSG00000164120 | 249.2262598 | 595.2225983 | 0.418711018 | 3.52959E-07 | 0.000143072 |
| ENSG00000124575 | 1725.132157 | 4946.608499 | 0.348750494 | 3.59545E-07 | 0.000144298 |
| ENSG00000067167 | 3075.47715 | 6607.956676 | 0.465420296 | 3.41468E-07 | 0.000141113 |
| ENSG00000047597 | 191.661561 | 510.3528052 | 0.375547188 | 3.78932E-07 | 0.000150588 |
| ENSG00000113810 | 1236.155442 | 2627.225138 | 0.470517514 | 3.92736E-07 | 0.000154559 |
| ENSG00000183134 | 296.3306772 | 74.32431547 | 3.986995041 | 5.16526E-07 | 0.000192086 |
| ENSG00000182557 | 206.541306 | 48.95975554 | 4.21859349 | 5.44859E-07 | 0.000197195 |
| ENSG00000235961 | 25.32747594 | 2.162959981 | 11.70963687 | 5.04202E-07 | 0.000191008 |
| ENSG00000128609 | 710.2445491 | 1534.630192 | 0.462811531 | 5.12448E-07 | 0.000192086 |
| ENSG00000061676 | 276.206566 | 822.0331828 | 0.336004156 | 5.44624E-07 | 0.000197195 |
| ENSG00000165929 | 3582.470481 | 7648.96111 | 0.468360399 | 5.40787E-07 | 0.000197195 |
| ENSG00000104979 | 423.8891838 | 155.1562843 | 2.732014276 | 5.95114E-07 | 0.000213477 |
| ENSG00000214212 | 936.4993259 | 379.2824483 | 2.469134362 | 6.08106E-07 | 0.000216224 |
| ENSG00000273703 | 69.22775325 | 217.0185182 | 0.318994682 | 6.44179E-07 | 0.000225102 |
| ENSG00000073792 | 221.4641765 | 562.5201939 | 0.393699958 | 6.3784E-07 | 0.000224825 |
| ENSG00000254893 | 208.1606015 | 809.9403844 | 0.257007312 | 7.52905E-07 | 0.000258338 |
| ENSG00000125356 | 430.5574478 | 161.4300465 | 2.667145659 | 7.95428E-07 | 0.000266469 |
| ENSG00000184792 | 55.93278915 | 248.9328012 | 0.224690314 | 9.32469E-07 | 0.000297619 |
| ENSG00000182899 | 7137.931708 | 3012.817751 | 2.369188015 | 9.23221E-07 | 0.000297619 |
| ENSG00000185697 | 1604.345411 | 3527.097529 | 0.454862787 | 1.04451E-06 | 0.0003232 |
| ENSG00000229117 | 15323.51503 | 6073.676765 | 2.522938843 | 1.11571E-06 | 0.000342616 |
| ENSG00000132970 | 91.54657727 | 327.3067002 | 0.279696618 | 1.17241E-06 | 0.000357319 |
| ENSG00000166200 | 1236.776792 | 2542.487615 | 0.486443586 | 1.22855E-06 | 0.000368883 |
| ENSG00000126524 | 621.0829322 | 1319.132255 | 0.470826886 | 1.21819E-06 | 0.000368502 |
| ENSG00000092067 | 99.18449741 | 19.21587586 | 5.161591286 | 1.4666E-06 | 0.000424632 |
| ENSG00000132465 | 1207.821056 | 5789.027211 | 0.208639727 | 1.52374E-06 | 0.000438047 |
| ENSG00000180354 | 1572.348303 | 3945.647259 | 0.398501995 | 1.54575E-06 | 0.000441247 |
| ENSG00000077458 | 756.4310788 | 1576.000218 | 0.479968892 | 1.69422E-06 | 0.000467178 |
| ENSG00000125910 | 2128.410801 | 871.7648017 | 2.441496601 | 1.68564E-06 | 0.000467178 |
| ENSG00000090104 | 177.7364999 | 836.9046228 | 0.212373662 | 1.63232E-06 | 0.000462701 |
| ENSG00000234545 | 362.1865626 | 792.8066552 | 0.456840972 | 1.66009E-06 | 0.000467178 |
| ENSG00000110852 | 1666.078033 | 3400.085872 | 0.490010575 | 1.68302E-06 | 0.000467178 |
| ENSG00000183248 | 1.447464778 | 19.05928197 | 0.075945399 | 1.99941E-06 | 0.00053673 |
| ENSG00000082438 | 658.954982 | 1360.22763 | 0.484444638 | 2.12591E-06 | 0.000566934 |
| ENSG00000275993 | 51.71031245 | 165.8276408 | 0.311831684 | 2.15791E-06 | 0.000571706 |
| ENSG00000197147 | 674.9174822 | 1407.384453 | 0.479554454 | 2.36436E-06 | 0.000610443 |
| ENSG00000103363 | 892.9911464 | 381.7279561 | 2.339339135 | 2.34076E-06 | 0.000608223 |
| ENSG00000177324 | 522.8192087 | 2758.205795 | 0.189550471 | 2.3211E-06 | 0.000607005 |
| ENSG00000114120 | 1333.010277 | 2682.36623 | 0.496953124 | 2.42569E-06 | 0.000620663 |
| ENSG00000113387 | 2378.920539 | 4794.250048 | 0.49620285 | 2.46324E-06 | 0.000624048 |
| ENSG00000117155 | 670.6729179 | 1751.630024 | 0.382885032 | 2.72905E-06 | 0.000682852 |
| ENSG00000151239 | 808.1636896 | 1645.459271 | 0.491147793 | 2.99346E-06 | 0.000709591 |
| ENSG00000060138 | 970.6922483 | 1964.652857 | 0.494078251 | 3.04134E-06 | 0.000716748 |
| ENSG00000204482 | 2677.747809 | 1032.745182 | 2.59284464 | 2.99038E-06 | 0.000709591 |
| ENSG00000247596 | 576.2282282 | 237.5032031 | 2.426191397 | 2.91596E-06 | 0.000707775 |
| ENSG00000173674 | 995.3980258 | 2279.097292 | 0.436751002 | 2.97724E-06 | 0.000709591 |
| ENSG00000142856 | 221.8679628 | 500.770346 | 0.443053317 | 2.94838E-06 | 0.000709591 |
| ENSG00000136108 | 411.8627717 | 867.9833046 | 0.474505408 | 3.13521E-06 | 0.000730377 |
| ENSG00000002919 | 726.0278342 | 313.1507476 | 2.318461124 | 3.24548E-06 | 0.000747474 |
| ENSG00000165195 | 353.1445508 | 763.5134072 | 0.462525671 | 3.23581E-06 | 0.000747474 |
| ENSG00000148773 | 259.6027364 | 632.040666 | 0.410737395 | 3.31623E-06 | 0.000759455 |
| ENSG00000163519 | 1006.49073 | 2089.187081 | 0.481761896 | 3.38779E-06 | 0.000765999 |
| ENSG00000128590 | 437.59125 | 925.7677338 | 0.472679306 | 4.28262E-06 | 0.000938356 |
| ENSG00000151693 | 332.0025347 | 1192.110602 | 0.278499775 | 4.40853E-06 | 0.000960751 |
| ENSG00000137818 | 12167.22261 | 5341.358957 | 2.277926406 | 4.66479E-06 | 0.001005783 |
| ENSG00000155330 | 187.6950565 | 428.0742006 | 0.438463837 | 4.79079E-06 | 0.001022076 |
| ENSG00000048462 | 20.53093149 | 82.25924853 | 0.249588124 | 5.01626E-06 | 0.001059031 |
| ENSG00000136824 | 378.1718597 | 789.52166 | 0.478988581 | 5.17744E-06 | 0.001087397 |
| ENSG00000163291 | 249.716136 | 545.7435058 | 0.457570513 | 5.63444E-06 | 0.001130654 |
| ENSG00000168116 | 282.5388274 | 608.7800273 | 0.464106598 | 5.60643E-06 | 0.001130631 |
| ENSG00000182752 | 1.63921609 | 15.4310083 | 0.106228709 | 5.51735E-06 | 0.001123848 |
| ENSG00000023909 | 401.7571962 | 862.8289591 | 0.465627854 | 5.5661E-06 | 0.001128109 |
| ENSG00000006634 | 310.5527555 | 666.6682664 | 0.465828016 | 5.71351E-06 | 0.001140872 |
| ENSG00000198502 | 1605.887495 | 17.38797684 | 92.35620161 | 5.47861E-06 | 0.001121592 |
| ENSG00000183508 | 1332.042625 | 2826.694105 | 0.47123692 | 5.93862E-06 | 0.00118001 |
| ENSG00000105967 | 1048.710742 | 2128.781462 | 0.492634289 | 6.06329E-06 | 0.001198904 |
| ENSG00000130255 | 4561.665813 | 2149.158402 | 2.122535876 | 6.23197E-06 | 0.001208674 |
| ENSG00000171848 | 75.55982376 | 217.9063727 | 0.346753621 | 6.30053E-06 | 0.001216152 |
| ENSG00000147145 | 6.052053339 | 36.11996733 | 0.167554231 | 6.2134E-06 | 0.001208674 |
| ENSG00000131747 | 106.9832378 | 285.4571086 | 0.374778678 | 6.20375E-06 | 0.001208674 |
| ENSG00000006327 | 102.0232914 | 17.25764646 | 5.911773171 | 6.78599E-06 | 0.001297501 |
| ENSG00000182253 | 201.6478895 | 581.931432 | 0.346514861 | 7.18474E-06 | 0.001367294 |
| ENSG00000180914 | 25.95893743 | 85.77637733 | 0.302635041 | 7.44024E-06 | 0.001409299 |
| ENSG00000277224 | 455.323218 | 940.5641663 | 0.484095859 | 7.86893E-06 | 0.001483568 |
| ENSG00000143507 | 207.8293148 | 471.6673634 | 0.440626872 | 8.12341E-06 | 0.001507949 |
| ENSG00000124762 | 529.2257765 | 1071.708665 | 0.493814965 | 8.09029E-06 | 0.001507949 |
| ENSG00000183484 | 994.0562083 | 455.904755 | 2.180403247 | 8.29229E-06 | 0.001520941 |
| ENSG00000169504 | 1151.194688 | 2636.112697 | 0.436701621 | 8.36896E-06 | 0.00152809 |
| ENSG00000113638 | 332.909574 | 745.8331003 | 0.446359345 | 8.44169E-06 | 0.001534456 |
| ENSG00000146757 | 667.286919 | 1354.654664 | 0.492588212 | 8.2716E-06 | 0.001520941 |
| ENSG00000255529 | 287.7970866 | 612.3956547 | 0.469952855 | 9.63292E-06 | 0.001675839 |
| ENSG00000180694 | 381.4179021 | 822.6218111 | 0.463661305 | 9.98018E-06 | 0.001721474 |
| ENSG00000147036 | 120.0806357 | 305.4171652 | 0.393169243 | 1.02644E-05 | 0.001762991 |
| ENSG00000197614 | 9.153706616 | 0.306633149 | 29.85230605 | 1.07516E-05 | 0.00183116 |
| ENSG00000124172 | 2110.431081 | 1015.148287 | 2.07893872 | 1.10851E-05 | 0.001880057 |
| ENSG00000151838 | 29.45914285 | 97.36738341 | 0.302556583 | 1.12424E-05 | 0.001890915 |
| ENSG00000189057 | 73.26795704 | 210.8958551 | 0.347412978 | 1.28835E-05 | 0.002131563 |
| ENSG00000109771 | 47.76351986 | 132.5075175 | 0.360458944 | 1.39172E-05 | 0.002283937 |
| ENSG00000249992 | 16.29278747 | 65.2871045 | 0.249555982 | 1.52084E-05 | 0.002456072 |
| ENSG00000148488 | 77.86542877 | 193.5524057 | 0.402296363 | 1.59585E-05 | 0.002546759 |
| ENSG00000241553 | 2148.300335 | 1057.963614 | 2.030599452 | 1.59345E-05 | 0.002546759 |
| ENSG00000106003 | 1812.552449 | 820.2436326 | 2.209773263 | 1.63186E-05 | 0.00257383 |
| ENSG00000127252 | 11.77591396 | 51.59791035 | 0.228224629 | 1.85624E-05 | 0.00285195 |
| ENSG00000198258 | 864.1501453 | 385.559261 | 2.241290076 | 1.90595E-05 | 0.002915383 |
| ENSG00000117400 | 114.1657652 | 377.2642201 | 0.302614876 | 1.97589E-05 | 0.002966389 |
| ENSG00000131203 | 72.28744244 | 15.16130837 | 4.767889464 | 1.97544E-05 | 0.002966389 |
| ENSG00000173153 | 558.4522784 | 252.7267449 | 2.209707875 | 1.96811E-05 | 0.002966389 |
| ENSG00000139618 | 246.0589603 | 512.7180583 | 0.479910852 | 2.03849E-05 | 0.003042995 |
| ENSG00000131469 | 5533.894055 | 2502.512034 | 2.211335642 | 2.11802E-05 | 0.003121966 |
| ENSG00000173542 | 1815.913512 | 4300.129521 | 0.422292748 | 2.14673E-05 | 0.003141427 |
| ENSG00000145431 | 252.2683882 | 570.6881141 | 0.442042478 | 2.16922E-05 | 0.003162932 |
| ENSG00000050628 | 23.38922612 | 76.55690897 | 0.305514243 | 2.21833E-05 | 0.00321143 |
| ENSG00000137267 | 15.51024828 | 60.8685453 | 0.254815491 | 2.21018E-05 | 0.003211101 |
| ENSG00000250510 | 224.314487 | 83.16339946 | 2.697274143 | 2.2387E-05 | 0.003229385 |
| ENSG00000069869 | 209.1267618 | 442.4824575 | 0.472621588 | 2.40973E-05 | 0.003427316 |
| ENSG00000165169 | 409.2390492 | 895.5550991 | 0.456966913 | 2.46219E-05 | 0.003474085 |
| ENSG00000136783 | 236.1168098 | 496.9928372 | 0.475090971 | 2.49421E-05 | 0.003474322 |
| ENSG00000130165 | 315.7284491 | 133.4342488 | 2.366172493 | 2.5157E-05 | 0.003492263 |
| ENSG00000112759 | 302.0769519 | 92.38185028 | 3.269873368 | 2.48547E-05 | 0.003474085 |
| ENSG00000126267 | 1299.436661 | 628.714797 | 2.066814185 | 2.54234E-05 | 0.003517187 |
| ENSG00000187010 | 24.6636747 | 110.6540569 | 0.222889927 | 2.59533E-05 | 0.003566161 |
| ENSG00000197837 | 763.8221682 | 353.6124135 | 2.160054735 | 2.61945E-05 | 0.003587139 |
| ENSG00000122644 | 260.8426932 | 553.0673528 | 0.471629164 | 2.72795E-05 | 0.003710659 |
| ENSG00000160789 | 346.660884 | 742.6430934 | 0.466793386 | 2.76714E-05 | 0.003751373 |
| ENSG00000091181 | 701.2222007 | 170.6399022 | 4.109368277 | 2.88957E-05 | 0.00386563 |
| ENSG00000187109 | 8351.949386 | 18200.14922 | 0.458894556 | 2.92147E-05 | 0.003869987 |
| ENSG00000126264 | 1090.468348 | 526.7643723 | 2.070125478 | 2.96832E-05 | 0.003881316 |
| ENSG00000157856 | 9.778627894 | 0.849680264 | 11.50859718 | 2.96736E-05 | 0.003881316 |
| ENSG00000123739 | 573.4414295 | 1487.469802 | 0.38551467 | 3.00861E-05 | 0.003921352 |
| ENSG00000105971 | 10.7626604 | 45.61464408 | 0.235947482 | 3.23499E-05 | 0.004047233 |
| ENSG00000085117 | 1237.295824 | 613.536859 | 2.016660949 | 3.26169E-05 | 0.004068086 |
| ENSG00000187837 | 2542.979022 | 6749.549281 | 0.376762791 | 3.36027E-05 | 0.004140076 |
| ENSG00000149218 | 953.0992888 | 2403.944827 | 0.39647303 | 3.35268E-05 | 0.004140076 |
| ENSG00000175938 | 132.3817894 | 46.55966689 | 2.843271833 | 3.52219E-05 | 0.004313353 |
| ENSG00000146376 | 1077.939742 | 3128.74835 | 0.344527466 | 3.72572E-05 | 0.004521624 |
| ENSG00000109805 | 26.90636692 | 89.11529958 | 0.301927582 | 3.81095E-05 | 0.00461125 |
| ENSG00000105404 | 706.5530496 | 320.2910318 | 2.205972005 | 3.90882E-05 | 0.004673866 |
| ENSG00000106244 | 824.948892 | 395.9202192 | 2.083624054 | 3.90195E-05 | 0.004673866 |
| ENSG00000169692 | 331.8591673 | 143.9012861 | 2.306158453 | 3.86053E-05 | 0.004657343 |
| ENSG00000152583 | 0.166314414 | 6.638932779 | 0.025051378 | 3.88587E-05 | 0.004673866 |
| ENSG00000138160 | 78.09260324 | 196.7857698 | 0.396840703 | 3.99166E-05 | 0.004758877 |
| ENSG00000171860 | 804.5460641 | 300.3292696 | 2.678879968 | 4.01686E-05 | 0.004760914 |
| ENSG00000232112 | 1047.313222 | 512.4331345 | 2.043804649 | 4.17437E-05 | 0.004890404 |
| ENSG00000076604 | 135.8021985 | 295.2697786 | 0.459925832 | 4.3564E-05 | 0.005045338 |
| ENSG00000112306 | 9360.707982 | 4036.231059 | 2.319170495 | 4.34024E-05 | 0.005041024 |
| ENSG00000105825 | 1.335713507 | 11.62174664 | 0.114932251 | 4.60642E-05 | 0.005289557 |
| ENSG00000100473 | 52.43440109 | 145.0830469 | 0.361409567 | 4.67037E-05 | 0.005332777 |
| ENSG00000136527 | 5169.945628 | 10929.11574 | 0.47304336 | 4.74454E-05 | 0.0053571 |
| ENSG00000233822 | 269.1326576 | 546.9422172 | 0.492067807 | 4.74213E-05 | 0.0053571 |
| ENSG00000126787 | 14.90119614 | 58.06955897 | 0.256609425 | 4.72643E-05 | 0.0053571 |
| ENSG00000274618 | 674.3308372 | 263.8274523 | 2.555954019 | 4.84396E-05 | 0.005398123 |
| ENSG00000276203 | 2.802692758 | 25.03458228 | 0.111952847 | 4.84371E-05 | 0.005398123 |
| ENSG00000169224 | 121.8611933 | 319.9701483 | 0.380851757 | 4.94038E-05 | 0.005441802 |
| ENSG00000112742 | 20.15122033 | 65.25346413 | 0.308814568 | 4.92624E-05 | 0.005441016 |
| ENSG00000151468 | 7.674942121 | 37.45827204 | 0.204893117 | 5.04115E-05 | 0.005493093 |
| ENSG00000146350 | 274.6316769 | 549.71237 | 0.49959159 | 5.03213E-05 | 0.005493093 |
| ENSG00000133328 | 4.688217846 | 34.77385114 | 0.134820208 | 5.13289E-05 | 0.005578061 |
| ENSG00000114487 | 48.50903308 | 151.8642048 | 0.319423745 | 5.17326E-05 | 0.005606896 |
| ENSG00000196747 | 223.5266416 | 541.0354329 | 0.413146031 | 5.31799E-05 | 0.005733108 |
| ENSG00000081377 | 557.3705712 | 1418.212749 | 0.393009139 | 5.665E-05 | 0.006058854 |
| ENSG00000175701 | 121.9908217 | 43.16470157 | 2.826170859 | 5.83813E-05 | 0.006211253 |
| ENSG00000197061 | 836.7901936 | 371.8750769 | 2.250191652 | 5.93116E-05 | 0.00624881 |
| ENSG00000109321 | 35.02453354 | 149.0407226 | 0.234999757 | 6.12262E-05 | 0.006396399 |
| ENSG00000259332 | 43.94610402 | 11.82602618 | 3.716049953 | 6.30277E-05 | 0.006534086 |
| ENSG00000072571 | 16.33552137 | 60.56916823 | 0.269700276 | 6.44908E-05 | 0.006668709 |
| ENSG00000151332 | 261.6314798 | 561.905121 | 0.465615048 | 6.50273E-05 | 0.006707079 |
| ENSG00000177119 | 3242.397471 | 6693.000366 | 0.484446032 | 6.7055E-05 | 0.006829328 |
| ENSG00000035403 | 7366.434749 | 15916.5455 | 0.462816178 | 6.65882E-05 | 0.006829328 |
| ENSG00000123500 | 5.84086026 | 32.38115962 | 0.180378354 | 6.69499E-05 | 0.006829328 |
| ENSG00000150787 | 260.3702161 | 522.8800795 | 0.497953979 | 6.79389E-05 | 0.006884762 |
| ENSG00000174917 | 217.605832 | 88.7443309 | 2.452053328 | 7.044E-05 | 0.007050088 |
| ENSG00000118193 | 37.4556234 | 107.9930864 | 0.34683353 | 7.01717E-05 | 0.007040617 |
| ENSG00000118276 | 99.42772967 | 223.7906931 | 0.444288939 | 6.99835E-05 | 0.00703916 |
| ENSG00000177425 | 158.0643702 | 332.9971657 | 0.474671818 | 6.99603E-05 | 0.00703916 |
| ENSG00000006747 | 6.514568329 | 28.59960299 | 0.227785271 | 7.48699E-05 | 0.007384063 |
| ENSG00000171502 | 65.71579298 | 185.6719047 | 0.35393504 | 7.61305E-05 | 0.007472034 |
| ENSG00000174151 | 808.8923671 | 399.5425043 | 2.024546471 | 7.97573E-05 | 0.007755496 |
| ENSG00000113083 | 6.156401367 | 50.71191881 | 0.121399496 | 8.15878E-05 | 0.00786383 |
| ENSG00000126432 | 757.9604235 | 371.1924352 | 2.041960858 | 8.41396E-05 | 0.00798735 |
| ENSG00000162614 | 309.3405244 | 1239.412791 | 0.249586358 | 8.99702E-05 | 0.008364548 |
| ENSG00000277157 | 304.7101961 | 127.5682149 | 2.388605941 | 9.27805E-05 | 0.008566871 |
| ENSG00000184730 | 3610.860492 | 1215.422648 | 2.970868197 | 9.41808E-05 | 0.008671062 |
| ENSG00000180549 | 151.3962496 | 45.33740169 | 3.339323471 | 9.55271E-05 | 0.008701552 |
| ENSG00000119326 | 73.5227804 | 195.367223 | 0.376331195 | 9.47145E-05 | 0.00868609 |
| ENSG00000180573 | 4819.385646 | 9781.812735 | 0.492688398 | 9.53152E-05 | 0.008701552 |
| ENSG00000100373 | 26.88015899 | 6.222146005 | 4.32007847 | 0.000116496 | 0.010133407 |
| ENSG00000177359 | 13.8076613 | 162.0989506 | 0.085180448 | 0.000117544 | 0.010202648 |
| ENSG00000169756 | 3660.417302 | 10877.50672 | 0.33651253 | 0.000119794 | 0.010331609 |
| ENSG00000123091 | 1998.946162 | 5305.117222 | 0.376795852 | 0.000119698 | 0.010331609 |
| ENSG00000112715 | 217.9478642 | 479.7068186 | 0.454335556 | 0.000119533 | 0.010331609 |
| ENSG00000132639 | 1.138552376 | 27.44230513 | 0.041488948 | 0.000124767 | 0.010624862 |
| ENSG00000156535 | 80.545692 | 195.2784864 | 0.412465774 | 0.000125301 | 0.010647984 |
| ENSG00000147650 | 152.9519977 | 328.8569406 | 0.46510193 | 0.000126331 | 0.010713031 |
| ENSG00000011426 | 30.92155689 | 89.44601782 | 0.345700766 | 0.00012734 | 0.010743438 |
| ENSG00000169398 | 454.3925128 | 989.6246044 | 0.459156443 | 0.000131807 | 0.01094833 |
| ENSG00000133739 | 105.6355035 | 229.3213156 | 0.460644067 | 0.000131419 | 0.01094833 |
| ENSG00000225663 | 364.2497037 | 169.2157845 | 2.152575215 | 0.000133284 | 0.011022924 |
| ENSG00000189184 | 5.433836472 | 0.169784186 | 32.00437337 | 0.000135399 | 0.011132656 |
| ENSG00000130176 | 3.822344659 | 20.55260251 | 0.18597862 | 0.00013502 | 0.011124079 |
| ENSG00000121552 | 1000.650751 | 478.7353147 | 2.090196232 | 0.000139231 | 0.011321366 |
| ENSG00000107863 | 1345.721271 | 3231.739418 | 0.416407729 | 0.000154038 | 0.012077214 |
| ENSG00000159217 | 7.7591127 | 0.196102039 | 39.56671088 | 0.000155843 | 0.012195146 |
| ENSG00000172967 | 12.58008204 | 1.661283681 | 7.572506841 | 0.0001573 | 0.012281873 |
| ENSG00000275126 | 86.74760626 | 29.83683972 | 2.907399278 | 0.000163588 | 0.012654626 |
| ENSG00000173626 | 96.28654623 | 267.328994 | 0.36017996 | 0.000165492 | 0.012693959 |
| ENSG00000164989 | 74.00634824 | 165.8410865 | 0.446248573 | 0.000167382 | 0.012801587 |
| ENSG00000158050 | 397.2175674 | 1059.63742 | 0.374861778 | 0.000172916 | 0.013076792 |
| ENSG00000162069 | 17.76656378 | 3.070920869 | 5.785418946 | 0.000175998 | 0.013235788 |
| ENSG00000150076 | 92.98537195 | 201.1454785 | 0.462279205 | 0.000176782 | 0.013249897 |
| ENSG00000130332 | 328.8080823 | 153.6112219 | 2.140521234 | 0.000187718 | 0.013942995 |
| ENSG00000105205 | 2143.786184 | 464.8171491 | 4.612106476 | 0.000192614 | 0.014247473 |
| ENSG00000233608 | 7.526401347 | 0.48083968 | 15.65262116 | 0.000198784 | 0.014597291 |
| ENSG00000197756 | 6710.209183 | 3191.761761 | 2.102352771 | 0.000206478 | 0.015026199 |
| ENSG00000125257 | 632.3340104 | 1467.337763 | 0.430939642 | 0.000205494 | 0.015026199 |
| ENSG00000134825 | 359.3934719 | 171.2560014 | 2.098574468 | 0.000206471 | 0.015026199 |
| ENSG00000153048 | 386.0488512 | 187.3407763 | 2.060677119 | 0.000209464 | 0.015089687 |
| ENSG00000015568 | 39.28779471 | 99.92799489 | 0.393161043 | 0.000208735 | 0.01508211 |
| ENSG00000169855 | 10.06533926 | 35.37341447 | 0.284545312 | 0.000210772 | 0.015148302 |
| ENSG00000144868 | 1.1319391 | 9.65650806 | 0.117220334 | 0.000216364 | 0.015467959 |
| ENSG00000185803 | 213.3763631 | 93.93283504 | 2.271584404 | 0.000225895 | 0.016007963 |
| ENSG00000154917 | 101.0089021 | 236.1002152 | 0.427822152 | 0.000230662 | 0.016232463 |
| ENSG00000115758 | 1214.247484 | 2941.066921 | 0.412859522 | 0.000230289 | 0.016232463 |
| ENSG00000280329 | 7.865033991 | 29.74139747 | 0.264447358 | 0.000240452 | 0.016689568 |
| ENSG00000167034 | 76.77877816 | 21.28478751 | 3.607213749 | 0.000242269 | 0.016758956 |
| ENSG00000135338 | 16.05441473 | 51.824491 | 0.30978432 | 0.000245371 | 0.016886465 |
| ENSG00000108691 | 30.57798058 | 5.710098315 | 5.35507077 | 0.000256336 | 0.017542864 |
| ENSG00000164116 | 311.659238 | 1168.399491 | 0.266740306 | 0.000259874 | 0.017674501 |
| ENSG00000182732 | 110.9131911 | 341.2018999 | 0.325066159 | 0.000261407 | 0.017748966 |
| ENSG00000164114 | 153.2426763 | 306.6999028 | 0.499650228 | 0.000278396 | 0.01850393 |
| ENSG00000184371 | 488.2031131 | 145.1441141 | 3.363574997 | 0.000288489 | 0.018861137 |
| ENSG00000197959 | 878.0094132 | 3007.91024 | 0.291900138 | 0.00029248 | 0.019015696 |
| ENSG00000138180 | 12.4402644 | 44.68794949 | 0.278380739 | 0.000293668 | 0.019015696 |
| ENSG00000235453 | 92.01753924 | 34.98645316 | 2.630090533 | 0.00029532 | 0.019092202 |
| ENSG00000198829 | 63.55418651 | 179.7546342 | 0.353560768 | 0.000296405 | 0.019111895 |
| ENSG00000047648 | 310.0217182 | 948.2911483 | 0.326926724 | 0.00029948 | 0.019175691 |
| ENSG00000108839 | 217.758332 | 684.4783629 | 0.318137641 | 0.000306071 | 0.019324893 |
| ENSG00000117724 | 131.7938919 | 306.1876909 | 0.430434978 | 0.000315957 | 0.019703565 |
| ENSG00000169020 | 205.0263918 | 91.54602937 | 2.239598956 | 0.000325341 | 0.020164664 |
| ENSG00000124882 | 47.82311538 | 212.5940404 | 0.224950404 | 0.000337967 | 0.020631778 |
| ENSG00000197415 | 124.9096833 | 363.2713278 | 0.343846799 | 0.000343753 | 0.020921982 |
| ENSG00000004468 | 390.7693663 | 854.2539276 | 0.457439356 | 0.000346782 | 0.021011702 |
| ENSG00000189114 | 311.3388892 | 151.0019289 | 2.06182061 | 0.000354572 | 0.021292719 |
| ENSG00000105583 | 305.1865211 | 147.9392664 | 2.062917633 | 0.00036091 | 0.021609311 |
| ENSG00000106366 | 34.48249741 | 100.5850721 | 0.342819234 | 0.000374166 | 0.02220618 |
| ENSG00000166963 | 350.8595877 | 945.2981303 | 0.371162892 | 0.000389678 | 0.022826014 |
| ENSG00000139734 | 16.32009519 | 48.54975714 | 0.336151943 | 0.00039323 | 0.022967663 |
| ENSG00000205038 | 473.6528281 | 1657.671992 | 0.285733746 | 0.000403974 | 0.023227092 |
| ENSG00000163535 | 115.6808224 | 232.5587729 | 0.497426181 | 0.000401842 | 0.023224387 |
| ENSG00000162722 | 506.8212209 | 1586.331805 | 0.319492567 | 0.000403354 | 0.023224387 |
| ENSG00000163507 | 107.3278075 | 220.7712197 | 0.486149452 | 0.000410456 | 0.023440083 |
| ENSG00000125652 | 235.0592352 | 109.4529537 | 2.147582383 | 0.000419382 | 0.023809029 |
| ENSG00000180304 | 2493.132035 | 1103.022802 | 2.260272436 | 0.000421487 | 0.02389506 |
| ENSG00000136492 | 96.70308663 | 200.158744 | 0.483131962 | 0.000432794 | 0.024298181 |
| ENSG00000142731 | 55.18919228 | 124.9953239 | 0.441530055 | 0.000432072 | 0.024291325 |
| ENSG00000273173 | 8.674972312 | 36.47975747 | 0.237802357 | 0.000441889 | 0.024670894 |
| ENSG00000242372 | 218.5443619 | 101.1082437 | 2.161489053 | 0.000451215 | 0.024884359 |
| ENSG00000156127 | 207.0748386 | 95.03301387 | 2.178977916 | 0.000449758 | 0.024837784 |
| ENSG00000163806 | 120.1460428 | 254.6729026 | 0.471766103 | 0.000456632 | 0.025115421 |
| ENSG00000276231 | 379.2106393 | 155.9684506 | 2.431329143 | 0.000462043 | 0.025343597 |
| ENSG00000256977 | 0.677276365 | 6.988360804 | 0.096914911 | 0.00046724 | 0.025492539 |
| ENSG00000144724 | 14.85559548 | 44.29882652 | 0.335349639 | 0.000466139 | 0.025492539 |
| ENSG00000144677 | 454.6703011 | 1506.307039 | 0.301844371 | 0.000472064 | 0.025650296 |
| ENSG00000165244 | 167.7721348 | 384.4666747 | 0.436376273 | 0.000491861 | 0.026524101 |
| ENSG00000118181 | 7709.857244 | 3832.364491 | 2.011775566 | 0.000493959 | 0.026524101 |
| ENSG00000197114 | 80.63224819 | 30.77050986 | 2.620439134 | 0.000497016 | 0.026613685 |
| ENSG00000140479 | 244.3172076 | 773.6843022 | 0.315784108 | 0.000498997 | 0.026684494 |
| ENSG00000176834 | 90.96478662 | 31.66397035 | 2.87281682 | 0.000505641 | 0.026933209 |
| ENSG00000029534 | 217.9915254 | 487.5916406 | 0.447078061 | 0.000519265 | 0.027335579 |
| ENSG00000165895 | 47.88400454 | 118.6152276 | 0.403691883 | 0.0005226 | 0.027368955 |
| ENSG00000172159 | 464.5909153 | 1290.802659 | 0.359924046 | 0.000537719 | 0.027766177 |
| ENSG00000186350 | 3951.188853 | 1864.326266 | 2.119365546 | 0.000542486 | 0.027905659 |
| ENSG00000157680 | 11.68304079 | 57.77223513 | 0.202225875 | 0.000551512 | 0.028120156 |
| ENSG00000122643 | 1631.367711 | 3346.583856 | 0.487472533 | 0.000550141 | 0.028120156 |
| ENSG00000150594 | 16.98620805 | 70.66879062 | 0.240363644 | 0.000548366 | 0.028120156 |
| ENSG00000177432 | 31.10872555 | 78.39271638 | 0.396831836 | 0.000560009 | 0.028446049 |
| ENSG00000184702 | 273.6032729 | 768.321786 | 0.356105056 | 0.000559661 | 0.028446049 |
| ENSG00000104312 | 611.1703513 | 1231.812236 | 0.496155447 | 0.000586345 | 0.029342594 |
| ENSG00000166816 | 27.12595359 | 7.55503815 | 3.590445614 | 0.000592677 | 0.029622909 |
| ENSG00000135116 | 72.92073995 | 20.57205528 | 3.544650204 | 0.000603315 | 0.02993316 |
| ENSG00000175482 | 91.2909757 | 36.60952069 | 2.493640287 | 0.000605227 | 0.029991297 |
| ENSG00000179988 | 58.63318042 | 131.8433353 | 0.444718577 | 0.000638235 | 0.030810027 |
| ENSG00000103184 | 117.6517449 | 333.1528702 | 0.353146424 | 0.000644503 | 0.030917092 |
| ENSG00000128266 | 500.3277378 | 1594.514004 | 0.313780711 | 0.000642648 | 0.030889048 |
| ENSG00000153071 | 813.643341 | 1812.619974 | 0.448876959 | 0.000658651 | 0.031148299 |
| ENSG00000187624 | 12.87425451 | 2.238489275 | 5.751313912 | 0.000661402 | 0.031210634 |
| ENSG00000143546 | 29016.84858 | 10282.88787 | 2.82185792 | 0.000663533 | 0.031274763 |
| ENSG00000164109 | 83.28229127 | 173.2091364 | 0.480819274 | 0.000675048 | 0.031706911 |
| ENSG00000267796 | 28.41542266 | 8.195794105 | 3.467073757 | 0.000690546 | 0.032063312 |
| ENSG00000228253 | 7.878958192 | 31.83041506 | 0.247529232 | 0.000688974 | 0.032027009 |
| ENSG00000159335 | 141.6632448 | 61.88076566 | 2.289293664 | 0.000698242 | 0.032253694 |
| ENSG00000100234 | 2.374145364 | 16.03239622 | 0.14808425 | 0.000711837 | 0.03260374 |
| ENSG00000179869 | 205.9959415 | 565.7265306 | 0.364126359 | 0.00071461 | 0.032656968 |
| ENSG00000135637 | 266.0384198 | 130.6832705 | 2.035749632 | 0.000735869 | 0.033477507 |
| ENSG00000276289 | 20.1716585 | 5.313298333 | 3.796447562 | 0.000758778 | 0.034212546 |
| ENSG00000116761 | 11.13848232 | 33.96347189 | 0.327954761 | 0.000756845 | 0.034163382 |
| ENSG00000172586 | 161.670383 | 73.18979646 | 2.208919697 | 0.000761352 | 0.034252371 |
| ENSG00000164308 | 1285.540221 | 2991.55488 | 0.429723095 | 0.000756168 | 0.034163382 |
| ENSG00000183092 | 56.49117449 | 14.28247848 | 3.95527811 | 0.000768656 | 0.034466209 |
| ENSG00000162390 | 62.47767226 | 22.31597052 | 2.799684298 | 0.000780949 | 0.034824841 |
| ENSG00000155366 | 308.9151276 | 153.6259732 | 2.010826173 | 0.000789731 | 0.03506216 |
| ENSG00000147180 | 43.72195683 | 98.95238916 | 0.44184842 | 0.000787661 | 0.035020385 |
| ENSG00000125753 | 7414.975608 | 3084.88656 | 2.403646119 | 0.000792824 | 0.035115719 |
| ENSG00000161677 | 103.8558824 | 42.42549212 | 2.447959405 | 0.000802284 | 0.035377505 |
| ENSG00000177191 | 335.779922 | 97.83671341 | 3.432044171 | 0.000810402 | 0.035551554 |
| ENSG00000140416 | 361.6852838 | 785.2690019 | 0.460587751 | 0.00080826 | 0.035534528 |
| ENSG00000120885 | 1971.475434 | 6224.30174 | 0.31673841 | 0.000823744 | 0.035891228 |
| ENSG00000136929 | 389.8910464 | 1228.433497 | 0.31738881 | 0.000840214 | 0.036312685 |
| ENSG00000169435 | 29.46434655 | 72.44658241 | 0.406704437 | 0.000838163 | 0.036298784 |
| ENSG00000068001 | 81.8188033 | 31.73018767 | 2.578579243 | 0.000846779 | 0.036476297 |
| ENSG00000177398 | 18.12486587 | 4.640544355 | 3.905762877 | 0.000849365 | 0.036496052 |
| ENSG00000106484 | 97.43064361 | 210.8365929 | 0.462114485 | 0.000845456 | 0.036458026 |
| ENSG00000174944 | 510.4298961 | 216.1229063 | 2.361757505 | 0.000859629 | 0.036873066 |
| ENSG00000121101 | 8.555760962 | 27.99627938 | 0.3056035 | 0.000864738 | 0.036974831 |
| ENSG00000162881 | 191.7348692 | 49.45597815 | 3.876879528 | 0.000868766 | 0.037068862 |
| ENSG00000102053 | 49.13492601 | 114.69528 | 0.428395362 | 0.00087403 | 0.037215119 |
| ENSG00000072422 | 270.4768884 | 744.4353185 | 0.363331618 | 0.000904376 | 0.038067381 |
| ENSG00000024526 | 5.409949414 | 21.0872286 | 0.256550992 | 0.000909373 | 0.038079997 |
| ENSG00000255819 | 190.5367344 | 402.71046 | 0.473135797 | 0.000908397 | 0.038079997 |
| ENSG00000158825 | 1043.185872 | 367.792457 | 2.836343846 | 0.00091538 | 0.038176159 |
| ENSG00000091262 | 99.59480511 | 39.23302198 | 2.538545339 | 0.000919786 | 0.038243577 |
| ENSG00000203812 | 294.3525795 | 715.1124564 | 0.41161719 | 0.000927022 | 0.038312062 |
| ENSG00000162367 | 456.8233005 | 1080.015002 | 0.422978662 | 0.00093886 | 0.038636244 |
| ENSG00000137941 | 72.46342125 | 149.8086058 | 0.483706666 | 0.000955672 | 0.039228808 |
| ENSG00000127578 | 25.33959295 | 7.286846268 | 3.477443056 | 0.000964741 | 0.039421158 |
| ENSG00000092758 | 112.352456 | 42.91099965 | 2.618267039 | 0.000963192 | 0.039397547 |
| ENSG00000137804 | 75.35435849 | 163.2260209 | 0.461656531 | 0.000961581 | 0.039371409 |
| ENSG00000235568 | 5894.265511 | 2324.705189 | 2.535489463 | 0.001008886 | 0.040570647 |
| ENSG00000101333 | 10.86247204 | 33.78563299 | 0.321511574 | 0.001024123 | 0.040965983 |
| ENSG00000140682 | 32.01991017 | 101.0149603 | 0.316981862 | 0.00103669 | 0.041249621 |
| ENSG00000111291 | 4.299195209 | 21.29530577 | 0.201884643 | 0.001058231 | 0.041808376 |
| ENSG00000166825 | 6697.288253 | 2782.328287 | 2.407080532 | 0.001064002 | 0.041913825 |
| ENSG00000103024 | 214.0073157 | 103.6052295 | 2.065603413 | 0.001070106 | 0.042031746 |
| ENSG00000169313 | 453.2866844 | 1303.256644 | 0.34781076 | 0.001068687 | 0.042016728 |
| ENSG00000061918 | 448.2724522 | 1390.22573 | 0.322445803 | 0.001063761 | 0.041913825 |
| ENSG00000113356 | 21.85100486 | 59.02697541 | 0.370186761 | 0.00109672 | 0.042541207 |
| ENSG00000169679 | 55.55567928 | 141.1786934 | 0.393513199 | 0.001106297 | 0.042830709 |
| ENSG00000123485 | 12.0913059 | 39.05686164 | 0.309582117 | 0.001151168 | 0.044146262 |
| ENSG00000241878 | 1719.033183 | 853.9492835 | 2.013038966 | 0.001161819 | 0.044386742 |
| ENSG00000167244 | 1.776656087 | 10.11053749 | 0.175723209 | 0.0011888 | 0.045204519 |
| ENSG00000274180 | 988.4905199 | 405.7720107 | 2.436073691 | 0.001205465 | 0.045620405 |
| ENSG00000165646 | 81.0959093 | 170.9503629 | 0.474382785 | 0.001202422 | 0.045551556 |
| ENSG00000088325 | 43.95746794 | 105.6376048 | 0.41611572 | 0.001217532 | 0.045824194 |
| ENSG00000185272 | 21.47094037 | 54.90058361 | 0.391087653 | 0.001246899 | 0.046500688 |
| ENSG00000101335 | 562.8760001 | 1791.491352 | 0.314194093 | 0.001259481 | 0.046794739 |
| ENSG00000204420 | 1417.73282 | 4529.915019 | 0.312971174 | 0.001296589 | 0.047941892 |
| ENSG00000148426 | 62.24959991 | 150.6872484 | 0.413104629 | 0.001303757 | 0.048043438 |
| ENSG00000276070 | 1125.904225 | 142.6742701 | 7.891431469 | 0.001338929 | 0.048895021 |
| ENSG00000164509 | 19.47648153 | 57.79356725 | 0.337000854 | 0.001345596 | 0.049011222 |
| ENSG00000088053 | 116.9865073 | 339.5256026 | 0.344558721 | 0.001377286 | 0.049625154 |
| ENSG00000165879 | 1212.239295 | 524.7966761 | 2.309921823 | 0.001374441 | 0.049610835 |
| ENSG00000158457 | 497.0927948 | 1024.272631 | 0.485312972 | 0.001388087 | 0.04988129 |
| ENSG00000165757 | 1.617558882 | 10.76642395 | 0.150241054 | 0.001403206 | 0.050202071 |
| ENSG00000145632 | 44.38612924 | 100.5563146 | 0.441405688 | 0.001411171 | 0.050361685 |
| ENSG00000185842 | 31.2184492 | 92.42610961 | 0.337766561 | 0.001419296 | 0.050510222 |
| ENSG00000154146 | 3715.892371 | 11116.27608 | 0.334274927 | 0.001460758 | 0.051399142 |
| ENSG00000159147 | 74.74250144 | 150.8905209 | 0.49534259 | 0.001458853 | 0.051376736 |
| ENSG00000095303 | 2155.436765 | 5180.366318 | 0.41607806 | 0.001469121 | 0.051510431 |
| ENSG00000149403 | 4.400887786 | 20.69873635 | 0.212616254 | 0.001482363 | 0.051755022 |
| ENSG00000122877 | 487.0433644 | 151.1562475 | 3.222118653 | 0.001481537 | 0.051755022 |
| ENSG00000165730 | 2.022489137 | 10.46373903 | 0.19328551 | 0.001481456 | 0.051755022 |
| ENSG00000276966 | 1964.03066 | 977.6929682 | 2.008841961 | 0.001520079 | 0.05230596 |
| ENSG00000041353 | 2618.573395 | 7463.936035 | 0.350830096 | 0.001517864 | 0.05230596 |
| ENSG00000174640 | 2.712597501 | 13.27422474 | 0.204350729 | 0.001516007 | 0.05230596 |
| ENSG00000143476 | 24.67148287 | 65.08181494 | 0.379084125 | 0.001545803 | 0.052825678 |
| ENSG00000070182 | 170.0380874 | 441.9733298 | 0.38472477 | 0.001556035 | 0.052825678 |
| ENSG00000143882 | 0.162753174 | 4.624377251 | 0.035194614 | 0.001552718 | 0.052825678 |
| ENSG00000280789 | 23.15305092 | 4.810151092 | 4.813372902 | 0.00159029 | 0.053640885 |
| ENSG00000129596 | 14.26782522 | 3.058566908 | 4.664872683 | 0.001586837 | 0.053640885 |
| ENSG00000188659 | 13.41057046 | 41.74003578 | 0.321287948 | 0.001610568 | 0.053998648 |
| ENSG00000157851 | 3.952865286 | 0.157004927 | 25.17669584 | 0.001644434 | 0.05478504 |
| ENSG00000127325 | 2.560227362 | 11.36390084 | 0.225294764 | 0.001652427 | 0.054902548 |
| ENSG00000140995 | 1799.168508 | 832.5405899 | 2.161058007 | 0.001657924 | 0.054950084 |
| ENSG00000061656 | 6.384258793 | 21.36857596 | 0.298768566 | 0.001652057 | 0.054902548 |
| ENSG00000186354 | 102.7791178 | 209.9384955 | 0.489567754 | 0.001675526 | 0.055268543 |
| ENSG00000150681 | 7807.583104 | 18886.81109 | 0.413388108 | 0.001697549 | 0.055654113 |
| ENSG00000141505 | 166.7975709 | 72.83816356 | 2.289974963 | 0.001695318 | 0.055643489 |
| ENSG00000116774 | 16.37112793 | 4.303007324 | 3.804578216 | 0.001706259 | 0.055821811 |
| ENSG00000204710 | 8.59055549 | 30.75998075 | 0.279277011 | 0.001714198 | 0.05594607 |
| ENSG00000188343 | 3.155570308 | 12.95047492 | 0.243664447 | 0.001727901 | 0.056257408 |
| ENSG00000157368 | 13.95349074 | 2.978662336 | 4.684482215 | 0.00173261 | 0.056320241 |
| ENSG00000081181 | 38.82310384 | 106.7528651 | 0.363672711 | 0.001761236 | 0.056907324 |
| ENSG00000123219 | 277.3513115 | 647.4767675 | 0.428357163 | 0.001789375 | 0.057474109 |
| ENSG00000087303 | 20.50601743 | 57.75982916 | 0.355022127 | 0.00179668 | 0.057571886 |
| ENSG00000172572 | 132.7754994 | 481.8677329 | 0.275543454 | 0.001843683 | 0.058568738 |
| ENSG00000137807 | 28.09202805 | 72.04006706 | 0.389950054 | 0.001841232 | 0.058536721 |
| ENSG00000185905 | 4374.99948 | 2148.364221 | 2.036432853 | 0.001859729 | 0.058801955 |
| ENSG00000119636 | 44.94838271 | 98.07122347 | 0.45832387 | 0.001920287 | 0.060246791 |
| ENSG00000105767 | 39.32572254 | 11.52029716 | 3.413603138 | 0.001918256 | 0.060229683 |
| ENSG00000117114 | 8.52485663 | 0.559816432 | 15.22795 | 0.001944494 | 0.060584223 |
| ENSG00000164181 | 809.4005335 | 2820.221037 | 0.28699897 | 0.0019361 | 0.060555405 |
| ENSG00000150637 | 4006.790627 | 8656.673966 | 0.46285567 | 0.001933369 | 0.060523489 |
| ENSG00000161955 | 72.72606369 | 30.43052619 | 2.389904901 | 0.001942768 | 0.060577007 |
| ENSG00000140022 | 760.6483159 | 1874.500957 | 0.405787105 | 0.002002646 | 0.061684834 |
| ENSG00000126460 | 19.59957588 | 5.766136138 | 3.399083096 | 0.002036475 | 0.062442153 |
| ENSG00000198108 | 5.697877716 | 24.39200711 | 0.233596099 | 0.002033868 | 0.062409414 |
| ENSG00000169607 | 12.64424509 | 37.42793854 | 0.337829055 | 0.002032102 | 0.062402471 |
| ENSG00000172014 | 10.22158345 | 46.4183196 | 0.220205805 | 0.002070543 | 0.063261002 |
| ENSG00000154188 | 236.0682702 | 503.4294763 | 0.468920239 | 0.002086432 | 0.063493634 |
| ENSG00000125735 | 644.8462241 | 226.5408345 | 2.846490018 | 0.002107888 | 0.06395451 |
| ENSG00000137801 | 3223.040484 | 8411.661857 | 0.383163344 | 0.002141954 | 0.064409586 |
| ENSG00000071909 | 41.13419053 | 93.75142861 | 0.438758013 | 0.002138456 | 0.064352134 |
| ENSG00000176340 | 58.75526907 | 24.14384473 | 2.433550651 | 0.002192101 | 0.065492964 |
| ENSG00000184221 | 208.8838375 | 64.6358059 | 3.231704696 | 0.002210891 | 0.06575088 |
| ENSG00000196683 | 1753.921543 | 870.0305755 | 2.015930926 | 0.002216795 | 0.065781678 |
| ENSG00000198478 | 2249.352403 | 7838.652344 | 0.286956521 | 0.002210544 | 0.06575088 |
| ENSG00000169228 | 60.11348907 | 20.3842105 | 2.949022189 | 0.002288637 | 0.067127272 |
| ENSG00000119862 | 867.9078783 | 1934.781506 | 0.448581856 | 0.002286283 | 0.067127272 |
| ENSG00000174837 | 1439.538205 | 663.4017656 | 2.169934238 | 0.002297825 | 0.067299366 |
| ENSG00000183307 | 261.1404557 | 108.1308546 | 2.415041078 | 0.002285264 | 0.067127272 |
| ENSG00000169247 | 131.8548358 | 377.4205259 | 0.349357883 | 0.00227776 | 0.067127272 |
| ENSG00000123096 | 60.17931433 | 120.9582601 | 0.497521329 | 0.002283133 | 0.067127272 |
| ENSG00000182747 | 5.771730995 | 28.97640194 | 0.199187291 | 0.002274177 | 0.067127272 |
| ENSG00000017427 | 3.297924449 | 24.85766313 | 0.132672345 | 0.002365286 | 0.06828836 |
| ENSG00000140932 | 848.866043 | 152.9347879 | 5.550509825 | 0.002418657 | 0.069091104 |
| ENSG00000051341 | 41.04248149 | 92.15931221 | 0.445342749 | 0.002434407 | 0.069345521 |
| ENSG00000213145 | 47.96718926 | 18.54831368 | 2.586067397 | 0.00242619 | 0.069257484 |
| ENSG00000185156 | 26.68094001 | 8.086555757 | 3.299419532 | 0.002441801 | 0.069403171 |
| ENSG00000136244 | 7.499163805 | 35.39017353 | 0.211899605 | 0.002417408 | 0.069091104 |
| ENSG00000152207 | 663.501645 | 310.3917936 | 2.137626248 | 0.002434013 | 0.069345521 |
| ENSG00000002726 | 31.36443073 | 3.829872399 | 8.189419244 | 0.002519692 | 0.070752177 |
| ENSG00000160712 | 7104.844983 | 3375.7503 | 2.104671362 | 0.002532661 | 0.070752177 |
| ENSG00000153162 | 317.8493076 | 741.634994 | 0.428579167 | 0.002585241 | 0.07167767 |
| ENSG00000125968 | 7.133386067 | 67.71070421 | 0.105350936 | 0.002592386 | 0.071826638 |
| ENSG00000079482 | 114.3065184 | 238.4619896 | 0.479349009 | 0.00260758 | 0.072148971 |
| ENSG00000231852 | 4.905213049 | 0.520719319 | 9.420071182 | 0.002651671 | 0.072920941 |
| ENSG00000105048 | 36.1719711 | 12.23494221 | 2.956448054 | 0.00265091 | 0.072920941 |
| ENSG00000005249 | 2933.301286 | 11053.11978 | 0.265382204 | 0.002685017 | 0.073538627 |
| ENSG00000150630 | 12.55389798 | 36.39837096 | 0.344902743 | 0.002703048 | 0.073832929 |
| ENSG00000092850 | 12.38202764 | 1.554796685 | 7.963759997 | 0.002752098 | 0.074594777 |
| ENSG00000187135 | 11.59047632 | 2.42746298 | 4.774728355 | 0.002800635 | 0.075380958 |
| ENSG00000276085 | 408.2097684 | 176.7919807 | 2.308983511 | 0.002829667 | 0.075745417 |
| ENSG00000117586 | 513.3870361 | 1188.586956 | 0.431930566 | 0.002828534 | 0.075745417 |
| ENSG00000244617 | 131.320627 | 44.66314393 | 2.940245926 | 0.002842556 | 0.075781712 |
| ENSG00000108960 | 1807.825493 | 5241.304401 | 0.344919004 | 0.002889124 | 0.076733012 |
| ENSG00000156869 | 159.3659139 | 63.11599468 | 2.524968746 | 0.002924785 | 0.077356461 |
| ENSG00000099998 | 18.06277528 | 3.009094538 | 6.002727747 | 0.002938711 | 0.077451651 |
| ENSG00000124491 | 8442.617426 | 21422.38606 | 0.394102571 | 0.002950832 | 0.077541467 |
| ENSG00000114268 | 1281.801664 | 515.9550535 | 2.484328151 | 0.002944959 | 0.077465279 |
| ENSG00000049323 | 901.2645603 | 2783.206155 | 0.323822423 | 0.003014137 | 0.078722964 |
| ENSG00000085733 | 716.5959856 | 1899.006504 | 0.377353097 | 0.003040852 | 0.079120548 |
| ENSG00000187808 | 91.4545815 | 41.12829653 | 2.223641367 | 0.003037961 | 0.079120548 |
| ENSG00000005961 | 1764.905399 | 5251.40534 | 0.336082493 | 0.003034352 | 0.079098052 |
| ENSG00000111537 | 58.66986471 | 160.8375974 | 0.364777053 | 0.003085682 | 0.079876062 |
| ENSG00000152413 | 51.54033443 | 105.40113 | 0.488992238 | 0.003100895 | 0.080009409 |
| ENSG00000185630 | 301.8491288 | 782.5600075 | 0.385720106 | 0.003082695 | 0.079876062 |
| ENSG00000091129 | 47.89081452 | 123.7432367 | 0.387017632 | 0.003099843 | 0.080009409 |
| ENSG00000197324 | 7790.412959 | 3605.327626 | 2.160805832 | 0.003156754 | 0.081092944 |
| ENSG00000131459 | 7.644680311 | 23.99772902 | 0.31855849 | 0.003174989 | 0.08124885 |
| ENSG00000225873 | 120.0282612 | 33.60314502 | 3.571935339 | 0.003183316 | 0.08135922 |
| ENSG00000239264 | 21.18780026 | 53.93173019 | 0.392863351 | 0.003232436 | 0.082200008 |
| ENSG00000182685 | 82.78461101 | 36.83151562 | 2.247656921 | 0.003321914 | 0.08363589 |
| ENSG00000147862 | 38.60123154 | 143.5956221 | 0.268819 | 0.003334019 | 0.083784546 |
| ENSG00000154099 | 0.516582409 | 5.260905672 | 0.098192677 | 0.003364544 | 0.084290345 |
| ENSG00000142046 | 150.2934528 | 74.94833918 | 2.005293973 | 0.003409715 | 0.085001726 |
| ENSG00000184786 | 53.93551929 | 109.2538476 | 0.493671578 | 0.003404331 | 0.084919724 |
| ENSG00000160180 | 12.06914303 | 0.901556636 | 13.38700481 | 0.003400216 | 0.084869315 |
| ENSG00000107147 | 11.20935499 | 2.822232557 | 3.971804155 | 0.003386817 | 0.084743595 |
| ENSG00000173110 | 1680.592641 | 530.1338674 | 3.170128801 | 0.003396623 | 0.084847548 |
| ENSG00000142408 | 52.78025842 | 12.49539301 | 4.223977459 | 0.003488163 | 0.08611005 |
| ENSG00000196878 | 15.83969994 | 43.40431313 | 0.364933777 | 0.003517409 | 0.086589848 |
| ENSG00000148180 | 224.3112852 | 107.9843612 | 2.077257139 | 0.003670926 | 0.089155775 |
| ENSG00000165028 | 32.1402312 | 68.18808401 | 0.471346741 | 0.003707479 | 0.089690499 |
| ENSG00000145423 | 5.923861059 | 0.779139105 | 7.603085274 | 0.00371649 | 0.089746558 |
| ENSG00000108576 | 78.11899594 | 200.9016706 | 0.388841943 | 0.003734927 | 0.08992066 |
| ENSG00000172927 | 33.09599965 | 107.0183842 | 0.309255273 | 0.003707254 | 0.089690499 |
| ENSG00000178343 | 0.336284797 | 4.82743914 | 0.069661116 | 0.003732439 | 0.08992066 |
| ENSG00000243772 | 26.93415074 | 64.79145283 | 0.415705306 | 0.003763393 | 0.089999738 |
| ENSG00000096006 | 54.52282241 | 132.2072835 | 0.412404075 | 0.003758397 | 0.089999738 |
| ENSG00000279560 | 6.028392801 | 18.93881662 | 0.318308843 | 0.003751989 | 0.089992241 |
| ENSG00000144893 | 162.8619774 | 346.6521513 | 0.469813837 | 0.00378019 | 0.090180083 |
| ENSG00000167779 | 9.014203559 | 1.873362386 | 4.811777811 | 0.003868804 | 0.091353273 |
| ENSG00000152253 | 3.128902155 | 12.90322219 | 0.242489985 | 0.003891006 | 0.091353273 |
| ENSG00000166831 | 26.39902744 | 94.54972146 | 0.279207882 | 0.00389085 | 0.091353273 |
| ENSG00000103355 | 195.5360108 | 8.310530219 | 23.52870463 | 0.003907374 | 0.091605211 |
| ENSG00000163220 | 51400.52618 | 21534.22063 | 2.386922985 | 0.003931644 | 0.092067703 |
| ENSG00000141198 | 12.99382447 | 34.91744388 | 0.372129888 | 0.004024709 | 0.093705672 |
| ENSG00000115507 | 77.16063409 | 16.12770277 | 4.78435368 | 0.004022457 | 0.093705672 |
| ENSG00000180190 | 108.818821 | 260.6688837 | 0.417459957 | 0.004012074 | 0.093680543 |
| ENSG00000158560 | 10.57233875 | 32.065264 | 0.329713136 | 0.004021275 | 0.093705672 |
| ENSG00000163092 | 22.12066232 | 67.3248515 | 0.328566077 | 0.004093886 | 0.09465544 |
| ENSG00000163191 | 5548.289179 | 2414.0859 | 2.298298159 | 0.004114784 | 0.094822495 |
| ENSG00000105974 | 5.535127967 | 19.19698898 | 0.288333133 | 0.004123199 | 0.094879939 |
| ENSG00000090554 | 21.91454188 | 7.232564227 | 3.02998234 | 0.004178216 | 0.09566764 |
| ENSG00000105514 | 3876.53715 | 1739.777564 | 2.228179757 | 0.004171067 | 0.095630215 |
| ENSG00000007372 | 3.120291135 | 0.149628223 | 20.85362695 | 0.004163423 | 0.09553498 |
| ENSG00000180316 | 81.09172612 | 33.69624782 | 2.406550621 | 0.004222597 | 0.096213031 |
| ENSG00000244187 | 42.62401037 | 17.26569163 | 2.468711436 | 0.004220713 | 0.096213031 |
| ENSG00000166035 | 23.5778509 | 55.35759028 | 0.425919025 | 0.00424894 | 0.096704537 |
| ENSG00000022267 | 431.5518997 | 1186.036369 | 0.363860596 | 0.004289011 | 0.097019589 |
| ENSG00000164920 | 0.162753174 | 3.53898129 | 0.045988707 | 0.00432285 | 0.097510703 |
| ENSG00000152952 | 55.65676708 | 214.0208991 | 0.260052954 | 0.004333189 | 0.097689559 |
| ENSG00000155970 | 74.43027206 | 186.3942774 | 0.399316294 | 0.004345964 | 0.097819851 |
| ENSG00000138735 | 1584.098212 | 4779.978556 | 0.331402786 | 0.004424906 | 0.098822902 |
| ENSG00000170271 | 472.1951481 | 1144.98089 | 0.412404392 | 0.004444669 | 0.098972017 |
| ENSG00000184500 | 344.647532 | 1287.851121 | 0.267614421 | 0.004454662 | 0.099105232 |
| ENSG00000239732 | 8.699365101 | 1.989383422 | 4.372895141 | 0.004501087 | 0.099754816 |
| ENSG00000254470 | 2839.024302 | 1365.121695 | 2.079685872 | 0.004526662 | 0.10015735 |
| ENSG00000163808 | 38.80972792 | 83.97333821 | 0.462167263 | 0.004542914 | 0.100407315 |
| ENSG00000100034 | 3800.906755 | 1890.034906 | 2.011024634 | 0.004572759 | 0.100575408 |
| ENSG00000137198 | 157.6708731 | 412.5619933 | 0.382174984 | 0.004556905 | 0.100573595 |
| ENSG00000261408 | 4.271502845 | 0.495358485 | 8.623053765 | 0.004566265 | 0.100575408 |
| ENSG00000173210 | 290.6268597 | 931.322055 | 0.312058389 | 0.00463486 | 0.101169096 |
| ENSG00000186314 | 33.80919385 | 72.12175681 | 0.4687794 | 0.004629592 | 0.101164703 |
| ENSG00000185442 | 80.23793026 | 33.32047618 | 2.408066734 | 0.004628753 | 0.101164703 |
| ENSG00000071539 | 6.58273159 | 20.9114503 | 0.314790772 | 0.004655766 | 0.101463164 |
| ENSG00000179750 | 6.939961475 | 29.19270876 | 0.237729275 | 0.004616476 | 0.101150725 |
| ENSG00000187608 | 451.7785736 | 222.0795049 | 2.034310072 | 0.004624901 | 0.101164703 |
| ENSG00000154479 | 2.653451308 | 11.33917084 | 0.234007525 | 0.00460955 | 0.101096697 |
| ENSG00000166148 | 126.4134072 | 324.5812735 | 0.389466114 | 0.004683478 | 0.101848057 |
| ENSG00000115112 | 68.31130219 | 28.0340737 | 2.43672407 | 0.004707525 | 0.102206489 |
| ENSG00000184361 | 14.29957611 | 4.171144061 | 3.428214395 | 0.004727307 | 0.102315617 |
| ENSG00000122786 | 179.8347496 | 660.0017507 | 0.272476171 | 0.004787605 | 0.103116671 |
| ENSG00000103196 | 1819.440455 | 777.1575416 | 2.341147524 | 0.004821668 | 0.103465498 |
| ENSG00000138755 | 8.039450333 | 22.03773794 | 0.36480379 | 0.004840467 | 0.103680531 |
| ENSG00000170485 | 16.1580445 | 40.65807173 | 0.397412957 | 0.004838552 | 0.103680531 |
| ENSG00000105697 | 20.58984957 | 6.980446055 | 2.949646686 | 0.00492562 | 0.104514901 |
| ENSG00000102362 | 103.4058063 | 320.3199993 | 0.322820325 | 0.004982918 | 0.105035147 |
| ENSG00000124216 | 5.178912975 | 26.99972735 | 0.191813529 | 0.005118287 | 0.106699491 |
| ENSG00000136630 | 778.058405 | 378.8186382 | 2.053907402 | 0.005130145 | 0.106699491 |
| ENSG00000183914 | 6.715706456 | 23.10377427 | 0.29067573 | 0.005167563 | 0.10710705 |
| ENSG00000143603 | 8.413264321 | 23.34558665 | 0.360379221 | 0.005249364 | 0.10806652 |
| ENSG00000213937 | 35.09965272 | 9.540998387 | 3.678823881 | 0.005301198 | 0.10896758 |
| ENSG00000186352 | 30.93819671 | 66.01523626 | 0.468652367 | 0.005390925 | 0.109895687 |
| ENSG00000170647 | 6.241396924 | 18.34930053 | 0.340143588 | 0.00540412 | 0.110040313 |
| ENSG00000168078 | 2.844850516 | 12.14039378 | 0.234329344 | 0.005470629 | 0.110931442 |
| ENSG00000179593 | 23.6755252 | 7.856497294 | 3.013496259 | 0.005470057 | 0.110931442 |
| ENSG00000114626 | 5008.604598 | 2229.085082 | 2.246932896 | 0.00553028 | 0.111582832 |
| ENSG00000087237 | 9.37581914 | 26.53835394 | 0.353293168 | 0.005511738 | 0.111430565 |
| ENSG00000124588 | 822.9906526 | 392.86329 | 2.094852519 | 0.005524371 | 0.111563976 |
| ENSG00000248712 | 32.54340744 | 12.871627 | 2.528305664 | 0.005609218 | 0.112503534 |
| ENSG00000173391 | 24.22554538 | 75.58956256 | 0.320487969 | 0.005696133 | 0.113746571 |
| ENSG00000105427 | 8.831023224 | 2.099678225 | 4.205893608 | 0.005715796 | 0.113964477 |
| ENSG00000110400 | 381.0980604 | 190.4268339 | 2.001283394 | 0.005781598 | 0.114892311 |
| ENSG00000089505 | 158.6033652 | 72.15797595 | 2.198001857 | 0.005870181 | 0.116015507 |
| ENSG00000158806 | 3.642853807 | 0.319412408 | 11.40486003 | 0.005908559 | 0.116615324 |
| ENSG00000074416 | 360.043159 | 886.8168033 | 0.405994967 | 0.005905093 | 0.116615324 |
| ENSG00000111247 | 26.8490513 | 58.64497008 | 0.4578236 | 0.005946093 | 0.11703978 |
| ENSG00000198053 | 4764.989539 | 2338.651862 | 2.037494172 | 0.006021406 | 0.117854985 |
| ENSG00000166451 | 42.29588935 | 85.3449877 | 0.495587269 | 0.006014145 | 0.117769748 |
| ENSG00000178226 | 24.13706912 | 8.930003624 | 2.702918178 | 0.006048939 | 0.118029671 |
| ENSG00000196411 | 153.6829775 | 66.17001618 | 2.322547074 | 0.006048619 | 0.118029671 |
| ENSG00000171657 | 39.37414069 | 13.52533966 | 2.911138772 | 0.006129501 | 0.118507815 |
| ENSG00000100116 | 4.01903551 | 16.42353143 | 0.244712017 | 0.0061273 | 0.118507815 |
| ENSG00000148484 | 2679.429054 | 5411.066639 | 0.495175763 | 0.006110273 | 0.118450467 |
| ENSG00000108387 | 36.12816174 | 78.78134139 | 0.458587796 | 0.00612712 | 0.118507815 |
| ENSG00000174371 | 13.67325562 | 36.35198368 | 0.376135062 | 0.006107656 | 0.118450467 |
| ENSG00000196611 | 6.263445111 | 22.48516087 | 0.278559053 | 0.006191617 | 0.119342452 |
| ENSG00000205155 | 43.83690635 | 18.47421478 | 2.3728698 | 0.006315108 | 0.120974904 |
| ENSG00000111325 | 18.20306906 | 6.052097035 | 3.007729215 | 0.006345232 | 0.121322635 |
| ENSG00000187510 | 20.53356886 | 45.54945428 | 0.450797253 | 0.006344699 | 0.121322635 |
| ENSG00000162999 | 55.03435374 | 118.7910284 | 0.463287123 | 0.006409842 | 0.122154649 |
| ENSG00000182795 | 33.91746939 | 69.85388011 | 0.485548825 | 0.006424815 | 0.122340531 |
| ENSG00000168062 | 74.09623063 | 28.26507898 | 2.621476157 | 0.006485896 | 0.122910607 |
| ENSG00000158352 | 36.75948831 | 81.49686429 | 0.451054021 | 0.00658643 | 0.124279851 |
| ENSG00000178852 | 419.6443774 | 902.9756926 | 0.464734966 | 0.006647105 | 0.124747544 |
| ENSG00000148200 | 202.5876052 | 94.10918106 | 2.152686942 | 0.00669785 | 0.125250913 |
| ENSG00000186891 | 49.88702189 | 19.73453125 | 2.527905085 | 0.006752954 | 0.125969154 |
| ENSG00000141449 | 2.847900432 | 0.157004927 | 18.13892399 | 0.006744951 | 0.125877808 |
| ENSG00000260286 | 71.67029736 | 33.51913215 | 2.138190722 | 0.006739577 | 0.125835451 |
| ENSG00000255587 | 363.4424116 | 159.3169478 | 2.281253919 | 0.006806431 | 0.12667525 |
| ENSG00000138722 | 668.8336186 | 2211.140421 | 0.302483557 | 0.007066566 | 0.129729734 |
| ENSG00000165309 | 95.93484938 | 264.2101614 | 0.363100529 | 0.007011816 | 0.129192708 |
| ENSG00000047617 | 11.90077015 | 37.35326264 | 0.318600553 | 0.007001387 | 0.129192708 |
| ENSG00000172780 | 86.44850168 | 35.58416116 | 2.429409571 | 0.007082932 | 0.129853756 |
| ENSG00000114270 | 35.17030098 | 13.20603511 | 2.663199112 | 0.007065931 | 0.129729734 |
| ENSG00000141441 | 1.019359865 | 5.967272159 | 0.1708251 | 0.007043803 | 0.129546534 |
| ENSG00000214756 | 36.83426166 | 15.54642612 | 2.369307349 | 0.007033479 | 0.129474151 |
| ENSG00000156011 | 140.0303962 | 331.2469328 | 0.422737186 | 0.007056125 | 0.129655496 |
| ENSG00000242616 | 4.579687728 | 0.718993191 | 6.369584278 | 0.007113418 | 0.130059725 |
| ENSG00000105352 | 641.1869723 | 270.6936177 | 2.368681529 | 0.007144626 | 0.130571421 |
| ENSG00000198948 | 1522.537973 | 3872.616209 | 0.393154883 | 0.007228598 | 0.131675241 |
| ENSG00000091651 | 20.73774844 | 46.11194806 | 0.449726141 | 0.007376631 | 0.132504559 |
| ENSG00000165323 | 3.941034583 | 0.469040631 | 8.402330895 | 0.007403321 | 0.132653858 |
| ENSG00000163751 | 63.47224187 | 172.4590653 | 0.368042363 | 0.007393317 | 0.132605365 |
| ENSG00000137869 | 0.990680488 | 11.66746112 | 0.084909688 | 0.007432149 | 0.132806491 |
| ENSG00000281446 | 10.67290252 | 27.31477261 | 0.390737374 | 0.007470378 | 0.132945696 |
| ENSG00000099377 | 51.78695028 | 20.1598269 | 2.568819193 | 0.007463116 | 0.132945696 |
| ENSG00000170122 | 5.353739212 | 0.945457966 | 5.662588297 | 0.00763391 | 0.134594659 |
| ENSG00000196502 | 983.248251 | 443.998541 | 2.214530365 | 0.007692377 | 0.135334415 |
| ENSG00000126822 | 4135.324528 | 2059.158175 | 2.008259772 | 0.007770416 | 0.1361166 |
| ENSG00000136231 | 360.7270718 | 849.6235039 | 0.424572849 | 0.007803155 | 0.13643398 |
| ENSG00000143228 | 29.06535993 | 59.98141832 | 0.484572735 | 0.007992924 | 0.138458624 |
| ENSG00000067798 | 60.54189301 | 17.93541053 | 3.375551004 | 0.008006497 | 0.138623728 |
| ENSG00000148498 | 176.6855409 | 476.8644736 | 0.370515211 | 0.008066638 | 0.138986944 |
| ENSG00000281741 | 17.02699998 | 5.106432943 | 3.334421537 | 0.008088484 | 0.13922153 |
| ENSG00000131389 | 9185.505954 | 4567.872584 | 2.010893646 | 0.008174353 | 0.139868051 |
| ENSG00000163221 | 1283.375559 | 489.9647751 | 2.619322091 | 0.00824626 | 0.140623544 |
| ENSG00000140534 | 8.427511265 | 25.01593222 | 0.336885757 | 0.008237711 | 0.140596047 |
| ENSG00000134317 | 96.25664581 | 261.8541058 | 0.367596473 | 0.00854077 | 0.143257827 |
| ENSG00000082781 | 374.8030553 | 942.0990411 | 0.397838273 | 0.008602601 | 0.143737186 |
| ENSG00000115290 | 42.81090967 | 94.66809033 | 0.452221118 | 0.008618289 | 0.143939981 |
| ENSG00000259207 | 1703.231171 | 5613.463782 | 0.303418929 | 0.008646265 | 0.144139639 |
| ENSG00000163735 | 541.4700274 | 1426.026447 | 0.379705459 | 0.008664759 | 0.144240652 |
| ENSG00000170956 | 1401.210871 | 496.7814259 | 2.820578223 | 0.008681853 | 0.144406617 |
| ENSG00000134138 | 14.07866732 | 3.847969209 | 3.658726604 | 0.008706559 | 0.144521029 |
| ENSG00000212128 | 27.30916128 | 56.40197285 | 0.48418805 | 0.008724268 | 0.144755714 |
| ENSG00000106537 | 148.172184 | 306.4934008 | 0.483443309 | 0.008763172 | 0.144808473 |
| ENSG00000074660 | 523.9494038 | 215.9226311 | 2.426560852 | 0.008849247 | 0.145712972 |
| ENSG00000197461 | 170.3480838 | 396.0369504 | 0.430131794 | 0.008936312 | 0.14617975 |
| ENSG00000141570 | 60.77435554 | 28.72047964 | 2.116063391 | 0.009067998 | 0.147441353 |
| ENSG00000129749 | 47.31190823 | 17.75801118 | 2.664257148 | 0.009153815 | 0.148064597 |
| ENSG00000125650 | 41.72822277 | 18.53027713 | 2.251894156 | 0.009197992 | 0.14829447 |
| ENSG00000160307 | 30.93294548 | 64.18672612 | 0.481921222 | 0.009217525 | 0.148325681 |
| ENSG00000074276 | 41.83388117 | 19.00318299 | 2.201414426 | 0.009264808 | 0.148614557 |
| ENSG00000156970 | 23.15859862 | 52.96315964 | 0.43725863 | 0.009258404 | 0.148614557 |
| ENSG00000174482 | 18.4480256 | 43.93520306 | 0.419891666 | 0.009248595 | 0.148564883 |
| ENSG00000137261 | 134.2292278 | 63.89995185 | 2.100615476 | 0.009322461 | 0.149067431 |
| ENSG00000049247 | 321.5653212 | 160.766769 | 2.000197697 | 0.009336907 | 0.149180729 |
| ENSG00000186204 | 124.0371207 | 30.7966964 | 4.027611244 | 0.009317458 | 0.149046233 |
| ENSG00000029993 | 33.0974849 | 67.40741352 | 0.4910066 | 0.009364692 | 0.1493848 |
| ENSG00000206052 | 30.4784447 | 106.7558626 | 0.285496683 | 0.009441315 | 0.150138758 |
| ENSG00000138623 | 290.5948342 | 142.0889357 | 2.045161594 | 0.009526577 | 0.151079734 |
| ENSG00000060566 | 5.149317926 | 0.80948564 | 6.361222081 | 0.00957054 | 0.151318161 |
| ENSG00000181029 | 40.88043359 | 17.88508338 | 2.285727873 | 0.009618142 | 0.151699035 |
| ENSG00000176890 | 30.54394401 | 68.41860602 | 0.446427453 | 0.009672167 | 0.151844025 |
| ENSG00000118804 | 5.776863847 | 1.213394656 | 4.760910901 | 0.009659074 | 0.151772478 |
| ENSG00000185052 | 177.8242042 | 397.8135746 | 0.447003862 | 0.009714349 | 0.152129463 |
| ENSG00000106976 | 32.49342779 | 68.08649099 | 0.477237515 | 0.009760906 | 0.152645957 |
| ENSG00000102230 | 154.5928672 | 350.9849357 | 0.440454423 | 0.009822417 | 0.153371219 |
| ENSG00000105088 | 75.38985019 | 31.86947155 | 2.365582061 | 0.009851594 | 0.153493743 |
| ENSG00000072657 | 17.65468811 | 67.22993294 | 0.262601602 | 0.009886167 | 0.153833307 |
| ENSG00000064547 | 906.3155907 | 451.5258687 | 2.007228497 | 0.009903155 | 0.153841626 |
| ENSG00000160588 | 1610.527437 | 659.6932633 | 2.441327699 | 0.009975513 | 0.154393824 |
| ENSG00000127955 | 19.39791636 | 42.81670617 | 0.453045507 | 0.010045339 | 0.15512668 |
| ENSG00000139714 | 93.95247177 | 46.47554938 | 2.021546233 | 0.01007542 | 0.155258657 |
| ENSG00000112984 | 6.626748883 | 20.37457684 | 0.325245964 | 0.010143893 | 0.15586911 |
| ENSG00000054179 | 11.48840169 | 1.997802814 | 5.750518321 | 0.010231284 | 0.156795873 |
| ENSG00000203995 | 3.886581852 | 12.08165271 | 0.321692896 | 0.010272507 | 0.156984659 |
| ENSG00000171777 | 2588.425191 | 1211.803353 | 2.136010917 | 0.010263822 | 0.156938528 |
| ENSG00000233493 | 4.460807015 | 0.677724245 | 6.58203841 | 0.010470172 | 0.158954469 |
| ENSG00000092969 | 30.11856285 | 74.714532 | 0.403115191 | 0.01066475 | 0.160344531 |
| ENSG00000168004 | 27.82359434 | 8.206778264 | 3.390318764 | 0.010871523 | 0.161770492 |
| ENSG00000204160 | 5122.856739 | 2290.032307 | 2.237023785 | 0.010939101 | 0.162245314 |
| ENSG00000159761 | 47.44587719 | 21.36077374 | 2.221168473 | 0.01113621 | 0.163316303 |
| ENSG00000105976 | 0.96532272 | 6.241857354 | 0.154653121 | 0.011106974 | 0.16312362 |
| ENSG00000149516 | 161.1959088 | 323.9345705 | 0.497618727 | 0.011130982 | 0.163298714 |
| ENSG00000121570 | 9.708940354 | 27.47541401 | 0.353368301 | 0.011239122 | 0.164053947 |
| ENSG00000177300 | 0.527360858 | 4.527161691 | 0.116488187 | 0.011306568 | 0.16461552 |
| ENSG00000100526 | 10.23986269 | 27.84138945 | 0.367792804 | 0.011298379 | 0.164563344 |
| ENSG00000109099 | 36.92646337 | 13.46566542 | 2.742267998 | 0.011403024 | 0.165003765 |
| ENSG00000126246 | 44.52235479 | 19.93576473 | 2.23329054 | 0.011467878 | 0.165486096 |
| ENSG00000279386 | 2.625418225 | 0.167612353 | 15.66363209 | 0.011496416 | 0.165838869 |
| ENSG00000196420 | 9.482144909 | 2.46144089 | 3.852274067 | 0.011657186 | 0.167087711 |
| ENSG00000100678 | 54.92003379 | 144.7695632 | 0.379361743 | 0.011896786 | 0.168673043 |
| ENSG00000187537 | 0.337306606 | 3.41043587 | 0.098904251 | 0.012008654 | 0.169273207 |
| ENSG00000188305 | 307.7618654 | 121.5597697 | 2.531774008 | 0.012203836 | 0.170874782 |
| ENSG00000157388 | 102.9991769 | 48.20042737 | 2.136893436 | 0.012191533 | 0.170761506 |
| ENSG00000165131 | 7.536081546 | 1.904972482 | 3.956005462 | 0.012179382 | 0.170709279 |
| ENSG00000114805 | 51.451652 | 140.9231899 | 0.365104225 | 0.012242608 | 0.171181135 |
| ENSG00000218336 | 2.489611869 | 0.14236882 | 17.48705837 | 0.012405129 | 0.172619942 |
| ENSG00000152760 | 9.087679144 | 24.53146322 | 0.370449943 | 0.012393131 | 0.172592269 |
| ENSG00000182175 | 12.10235282 | 3.531268113 | 3.427197377 | 0.012450887 | 0.173019096 |
| ENSG00000175305 | 28.28201269 | 58.45707301 | 0.483808224 | 0.012522634 | 0.173611617 |
| ENSG00000053747 | 0.87058989 | 5.208287847 | 0.167154719 | 0.012599311 | 0.174126513 |
| ENSG00000161905 | 446.9362748 | 46.13295242 | 9.688005024 | 0.012653381 | 0.174695096 |
| ENSG00000169495 | 29.13067414 | 12.09585889 | 2.408317954 | 0.012767707 | 0.175377503 |
| ENSG00000122679 | 2.367678272 | 9.367730843 | 0.252748324 | 0.012761308 | 0.175349027 |
| ENSG00000196126 | 3062.991253 | 544.8086379 | 5.622141501 | 0.012815214 | 0.175751901 |
| ENSG00000257594 | 9.028186587 | 2.528547421 | 3.570503171 | 0.01307957 | 0.177614869 |
| ENSG00000178053 | 14.19950795 | 31.9852641 | 0.443939056 | 0.013103726 | 0.177704764 |
| ENSG00000170312 | 19.88038116 | 56.50784442 | 0.351816307 | 0.013023392 | 0.177208183 |
| ENSG00000100228 | 67.18848483 | 30.71232911 | 2.187671426 | 0.013060561 | 0.177511501 |
| ENSG00000173638 | 1817.942201 | 755.128385 | 2.407461084 | 0.013079016 | 0.177614869 |
| ENSG00000099994 | 43.30414695 | 20.04538655 | 2.160304908 | 0.013060068 | 0.177511501 |
| ENSG00000181274 | 3383.317666 | 1423.229137 | 2.377212199 | 0.013163665 | 0.17824258 |
| ENSG00000173809 | 9.288792801 | 25.01831902 | 0.371279653 | 0.01326946 | 0.179232776 |
| ENSG00000271321 | 6.65363859 | 17.50530278 | 0.380092745 | 0.013268736 | 0.179232776 |
| ENSG00000205710 | 126.0690883 | 340.5740314 | 0.370166474 | 0.013501248 | 0.180499045 |
| ENSG00000105507 | 57.51823732 | 159.8787477 | 0.35976162 | 0.013491092 | 0.180482309 |
| ENSG00000145386 | 38.85963817 | 79.34435229 | 0.489759347 | 0.013680154 | 0.182050241 |
| ENSG00000139053 | 5.216341822 | 14.78148947 | 0.352896901 | 0.013853214 | 0.183077746 |
| ENSG00000169403 | 6187.673092 | 2425.192879 | 2.551414836 | 0.013816351 | 0.183077746 |
| ENSG00000181631 | 5043.912418 | 2407.141618 | 2.095394961 | 0.013844247 | 0.183077746 |
| ENSG00000073282 | 24.56990786 | 49.51624412 | 0.49619894 | 0.013973891 | 0.183966111 |
| ENSG00000152078 | 16.3463832 | 35.75095797 | 0.457229236 | 0.014006968 | 0.184341709 |
| ENSG00000185201 | 11972.8646 | 4601.390833 | 2.602009921 | 0.014112515 | 0.185489884 |
| ENSG00000165304 | 15.44063098 | 35.93307844 | 0.429705209 | 0.014138425 | 0.1857702 |
| ENSG00000169181 | 3.585604382 | 0.513342615 | 6.984817313 | 0.014182932 | 0.186234254 |
| ENSG00000164100 | 4.897959147 | 13.94423943 | 0.351253231 | 0.014367519 | 0.187474044 |
| ENSG00000164879 | 3.330415775 | 10.60430728 | 0.314062549 | 0.014353442 | 0.187440973 |
| ENSG00000214215 | 5.082033025 | 13.80088074 | 0.368239761 | 0.014336448 | 0.187400164 |
| ENSG00000142178 | 61.96258225 | 148.2102465 | 0.418072189 | 0.014473156 | 0.18839736 |
| ENSG00000183762 | 522.3553911 | 155.3749751 | 3.361901687 | 0.014648152 | 0.18951894 |
| ENSG00000100365 | 2628.076069 | 1224.398979 | 2.146421318 | 0.014720254 | 0.190087761 |
| ENSG00000125319 | 4.093907567 | 12.19933888 | 0.335584379 | 0.014837805 | 0.191039309 |
| ENSG00000156381 | 31.62759896 | 63.39103288 | 0.498928595 | 0.015059936 | 0.192964071 |
| ENSG00000166073 | 3.729965202 | 0.531326745 | 7.020096842 | 0.015209988 | 0.1943685 |
| ENSG00000142405 | 1766.133458 | 819.061555 | 2.156289044 | 0.015237032 | 0.194652722 |
| ENSG00000267179 | 6.546852782 | 1.625354044 | 4.027954897 | 0.015312036 | 0.195340729 |
| ENSG00000127831 | 107.8384375 | 281.114389 | 0.383610522 | 0.015467524 | 0.196298087 |
| ENSG00000163485 | 5.027385084 | 0.976221355 | 5.149841333 | 0.015604024 | 0.197659095 |
| ENSG00000158856 | 606.0427918 | 1224.83304 | 0.494796247 | 0.015690394 | 0.198397374 |
| ENSG00000125657 | 10.97960332 | 25.0006536 | 0.439172651 | 0.015780265 | 0.198687958 |
| ENSG00000118898 | 11.17014238 | 2.691409613 | 4.15029445 | 0.015773784 | 0.198687958 |
| ENSG00000185245 | 392.683943 | 807.3800216 | 0.486368169 | 0.015834001 | 0.199202738 |
| ENSG00000175984 | 149.405014 | 326.8231338 | 0.457143325 | 0.016114773 | 0.200999506 |
| ENSG00000165682 | 249.0899706 | 591.5172475 | 0.421103479 | 0.016097892 | 0.200999506 |
| ENSG00000186827 | 60.5981081 | 29.6469484 | 2.043991418 | 0.016077982 | 0.200999506 |
| ENSG00000184389 | 47.77346843 | 15.25266312 | 3.132139486 | 0.016073781 | 0.200999506 |
| ENSG00000117650 | 5.095302716 | 15.86655878 | 0.321134708 | 0.01627257 | 0.202085974 |
| ENSG00000138395 | 10.91451024 | 3.155300861 | 3.459102861 | 0.016434417 | 0.203100339 |
| ENSG00000166086 | 547.3031082 | 1330.261874 | 0.411425088 | 0.016533409 | 0.203813681 |
| ENSG00000123700 | 1536.657677 | 525.9636503 | 2.921604327 | 0.016558744 | 0.2040148 |
| ENSG00000077935 | 0.176914618 | 2.655621901 | 0.066618903 | 0.016525292 | 0.203809514 |
| ENSG00000135407 | 320.9284905 | 121.0066301 | 2.652156252 | 0.016790163 | 0.205497048 |
| ENSG00000153347 | 19.60905273 | 50.68857689 | 0.386853487 | 0.016907979 | 0.206559649 |
| ENSG00000174175 | 635.8405493 | 1547.909057 | 0.410773841 | 0.017129017 | 0.208130902 |
| ENSG00000065328 | 10.15052827 | 26.29858635 | 0.385972392 | 0.017110348 | 0.2080912 |
| ENSG00000143942 | 16.04551703 | 34.38997238 | 0.466575456 | 0.017105178 | 0.208090756 |
| ENSG00000112053 | 134.5165608 | 64.22829101 | 2.094350615 | 0.017380918 | 0.209433865 |
| ENSG00000171241 | 32.94460551 | 67.66505057 | 0.486877719 | 0.017413528 | 0.209702122 |
| ENSG00000124615 | 21.37984995 | 7.778183579 | 2.748694439 | 0.017617281 | 0.211214579 |
| ENSG00000187800 | 197.8921229 | 519.6745596 | 0.380800097 | 0.017598068 | 0.211109116 |
| ENSG00000079393 | 8.81397934 | 2.792675538 | 3.156105756 | 0.01768005 | 0.211839925 |
| ENSG00000108821 | 0.658135176 | 4.163945536 | 0.158055664 | 0.017778426 | 0.212561762 |
| ENSG00000113594 | 0.96786215 | 5.224179759 | 0.185265859 | 0.01781941 | 0.212756934 |
| ENSG00000173171 | 357.6480445 | 175.069686 | 2.042889621 | 0.017777548 | 0.212561762 |
| ENSG00000119888 | 3.819368326 | 0.702185252 | 5.439260248 | 0.017877982 | 0.213034084 |
| ENSG00000136237 | 18.51725049 | 39.01877367 | 0.474572847 | 0.017979524 | 0.213419807 |
| ENSG00000187566 | 10.00481814 | 3.195022207 | 3.131376714 | 0.018079541 | 0.214222214 |
| ENSG00000282936 | 19.66514891 | 8.105324847 | 2.426201204 | 0.018134046 | 0.214663341 |
| ENSG00000180089 | 39.462685 | 18.56310713 | 2.125866361 | 0.018278232 | 0.215141781 |
| ENSG00000087495 | 7.381484665 | 19.50505517 | 0.378439569 | 0.018387426 | 0.215726287 |
| ENSG00000236320 | 176.2851271 | 426.1711799 | 0.413648636 | 0.018432576 | 0.215943486 |
| ENSG00000078018 | 34.83956358 | 72.22115087 | 0.482401113 | 0.018386803 | 0.215726287 |
| ENSG00000185532 | 64.28070214 | 207.3148572 | 0.310063172 | 0.018463394 | 0.216117143 |
| ENSG00000121236 | 14.96384555 | 4.65514107 | 3.214477354 | 0.018381831 | 0.215726287 |
| ENSG00000163464 | 7935.727256 | 2956.748703 | 2.683937004 | 0.018725497 | 0.217738959 |
| ENSG00000103740 | 30.29360291 | 74.41089675 | 0.407112456 | 0.018709243 | 0.217644213 |
| ENSG00000138685 | 21.74433415 | 88.27281137 | 0.246331048 | 0.01870853 | 0.217644213 |
| ENSG00000184661 | 12.89435153 | 30.34167285 | 0.424971675 | 0.018697534 | 0.217644213 |
| ENSG00000103534 | 0.173531623 | 2.920118824 | 0.05942622 | 0.01868222 | 0.21761028 |
| ENSG00000049130 | 24.1474436 | 77.41585556 | 0.311918578 | 0.018842056 | 0.218762931 |
| ENSG00000165626 | 71.85879652 | 25.01077882 | 2.87311311 | 0.019175046 | 0.220436889 |
