# Supplementary material for: LncRNA LYPLAL1-DT screening from type 2 diabetes with macrovascular complication contributes protective effects on human umbilical vein endothelial cells via regulating the miR-204-5p/SIRT1 axis
Source: Cell Death Discov. 2022 May 4;8:245. doi: 10.1038/s41420-022-01019-z (PMC9068612; doi:10.1038/s41420-022-01019-z)
Supplement: Supplementary file 7 — supplementary table 5 [file 41420_2022_1019_MOESM7_ESM.docx]

Table S5 The lncRNAs with predicted genes in the 8 validation positive lncRNAs and their predicted genes.

| **lncRNA** | **Gene** | **up/down** | **Gene name** |
| --- | --- | --- | --- |
| ENSG00000269902 | ENSG00000207870 | down | MIR221 |
| ENSG00000269902 | ENSG00000207725 | down | MIR222 |
| ENSG00000228063 | ENSG00000187699 | down | C2orf88 |
| ENSG00000228063 | ENSG00000169860 | down | P2RY1 |
| ENSG00000228063 | ENSG00000138798 | down | EGF |
| ENSG00000228063 | ENSG00000003436 | down | TFPI |
| ENSG00000228063 | ENSG00000143995 | down | MEIS1 |
| ENSG00000228063 | ENSG00000101856 | down | PGRMC1 |
| ENSG00000228063 | ENSG00000119280 | down | C1orf198 |
| ENSG00000228063 | ENSG00000107438 | down | PDLIM1 |
| ENSG00000228063 | ENSG00000102804 | down | TSC22D1 |
| ENSG00000228063 | ENSG00000168497 | down | CAVIN2 |
| ENSG00000228063 | ENSG00000113140 | down | SPARC |
| ENSG00000228063 | ENSG00000088826 | down | SMOX |
| ENSG00000228063 | ENSG00000204323 | down | SMIM5 |
| ENSG00000228063 | ENSG00000101162 | down | TUBB1 |
| ENSG00000228063 | ENSG00000156265 | down | MAP3K7CL |
| ENSG00000228063 | ENSG00000165702 | down | GFI1B |
| ENSG00000228063 | ENSG00000065534 | down | MYLK |
| MSTRG.74858 | ENSG00000232938 | down | RPL23AP87 |
| MSTRG.159327 | ENSG00000215045 | up | GRID2IP |
| MSTRG.159327 | ENSG00000231359 | up | RPL31P34 |
| MSTRG.159327 | ENSG00000136238 | up | RAC1 |
| MSTRG.30000 | ENSG00000102030 | up | NAA10 |
| MSTRG.30000 | ENSG00000149187 | up | CELF1 |
| MSTRG.30000 | ENSG00000165917 | up | RAPSN |
| MSTRG.30000 | ENSG00000165916 | up | PSMC3 |
| MSTRG.30000 | ENSG00000165915 | up | SLC39A13 |
| MSTRG.131944 | ENSG00000251149 | down | MTND5P5 |
| MSTRG.131944 | ENSG00000082438 | down | COBLL1 |
| MSTRG.131944 | ENSG00000174485 | down | DENND4A |
| MSTRG.152898 | ENSG00000156535 | up | CD109 |
| MSTRG.3528 | ENSG00000117400 | up | MPL |
| MSTRG.3528 | ENSG00000159479 | up | MED8 |
| MSTRG.3528 | ENSG00000117399 | up | CDC20 |
| MSTRG.3528 | ENSG00000066322 | up | ELOVL1 |
| MSTRG.3528 | ENSG00000066056 | up | TIE1 |
| MSTRG.3528 | ENSG00000198198 | up | SZT2 |
