# Supplementary figures and images for: LncRNA LYPLAL1-DT screening from type 2 diabetes with macrovascular complication contributes protective effects on human umbilical vein endothelial cells via regulating the miR-204-5p/SIRT1 axis
Source: Cell Death Discov. 2022 May 4;8:245. doi: 10.1038/s41420-022-01019-z (PMC9068612; doi:10.1038/s41420-022-01019-z)

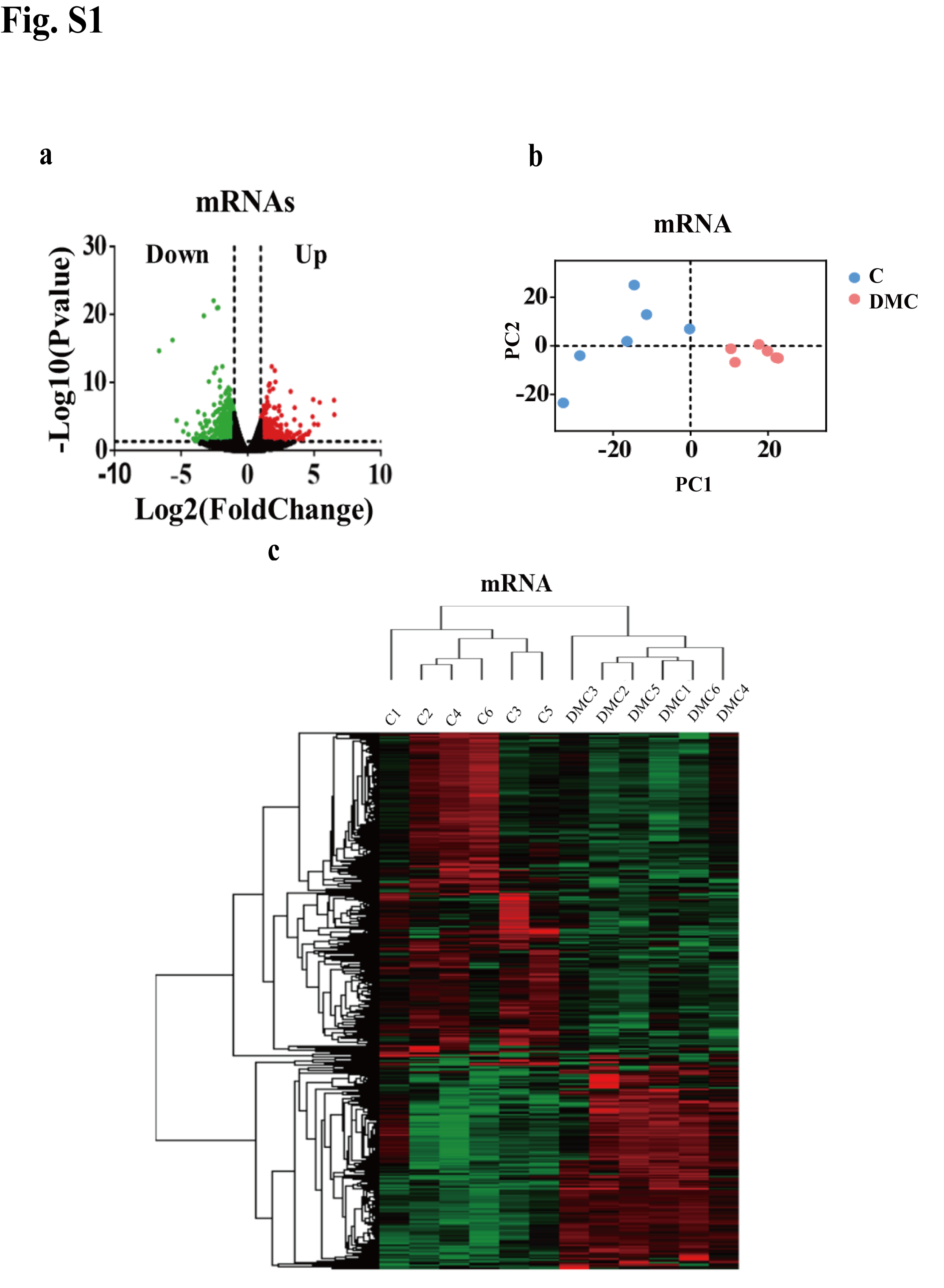

Supplement: Supplementary file 8 — Figure S1 [file 41420_2022_1019_MOESM8_ESM.png]

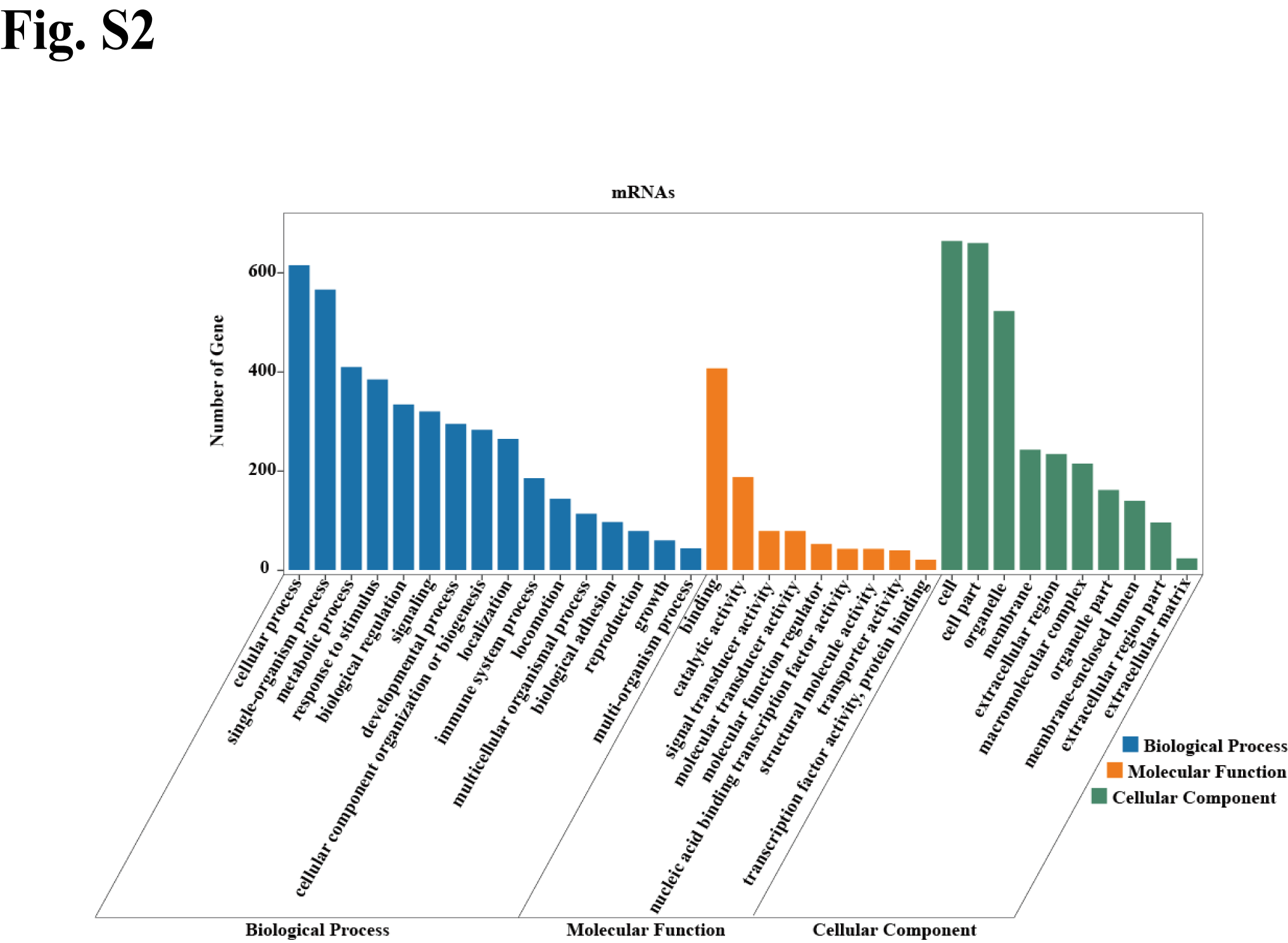

Supplement: Supplementary file 9 — Figure S2 [file 41420_2022_1019_MOESM9_ESM.png]

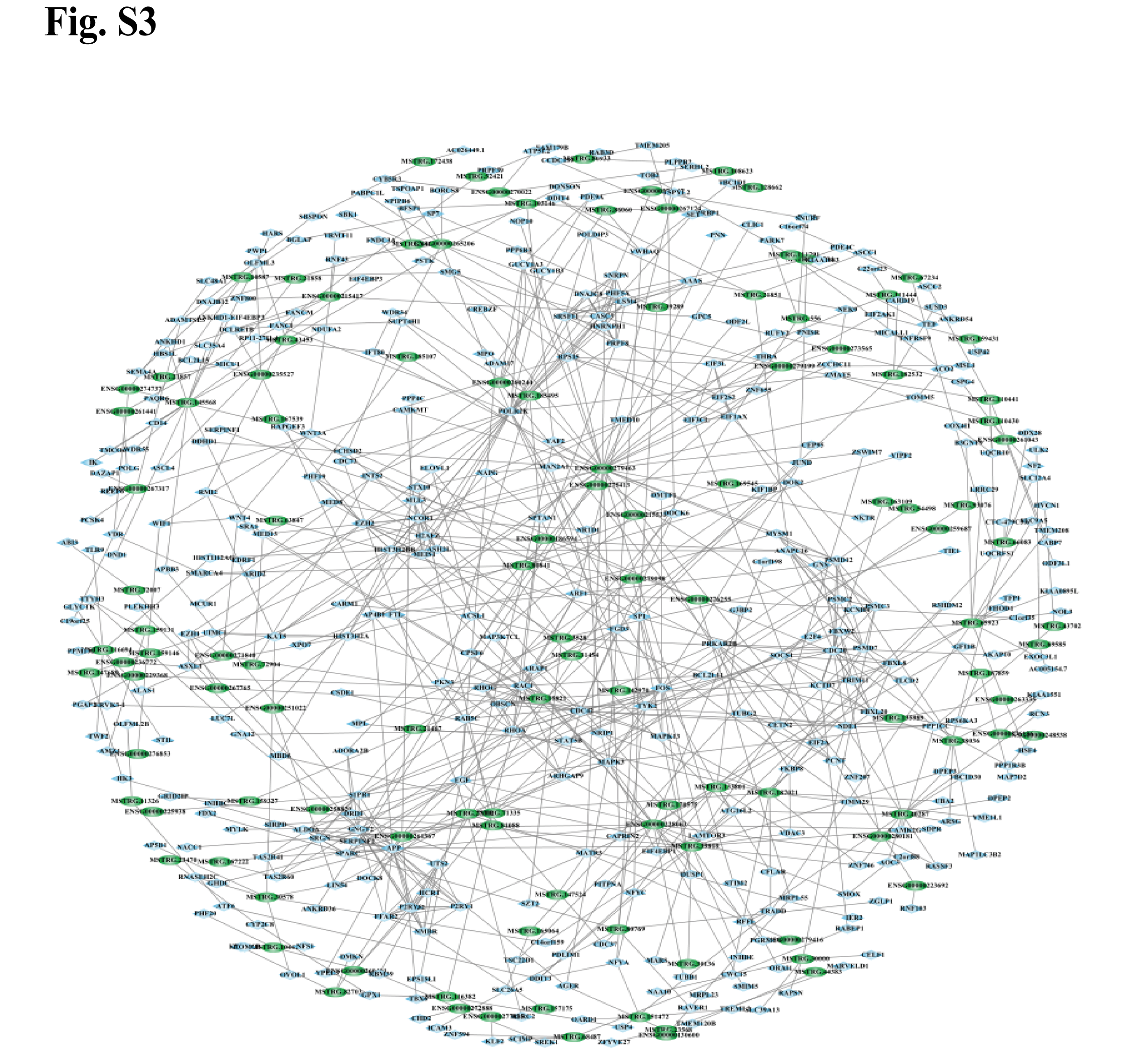

Supplement: Supplementary file 10 — Figure S3 [file 41420_2022_1019_MOESM10_ESM.png]

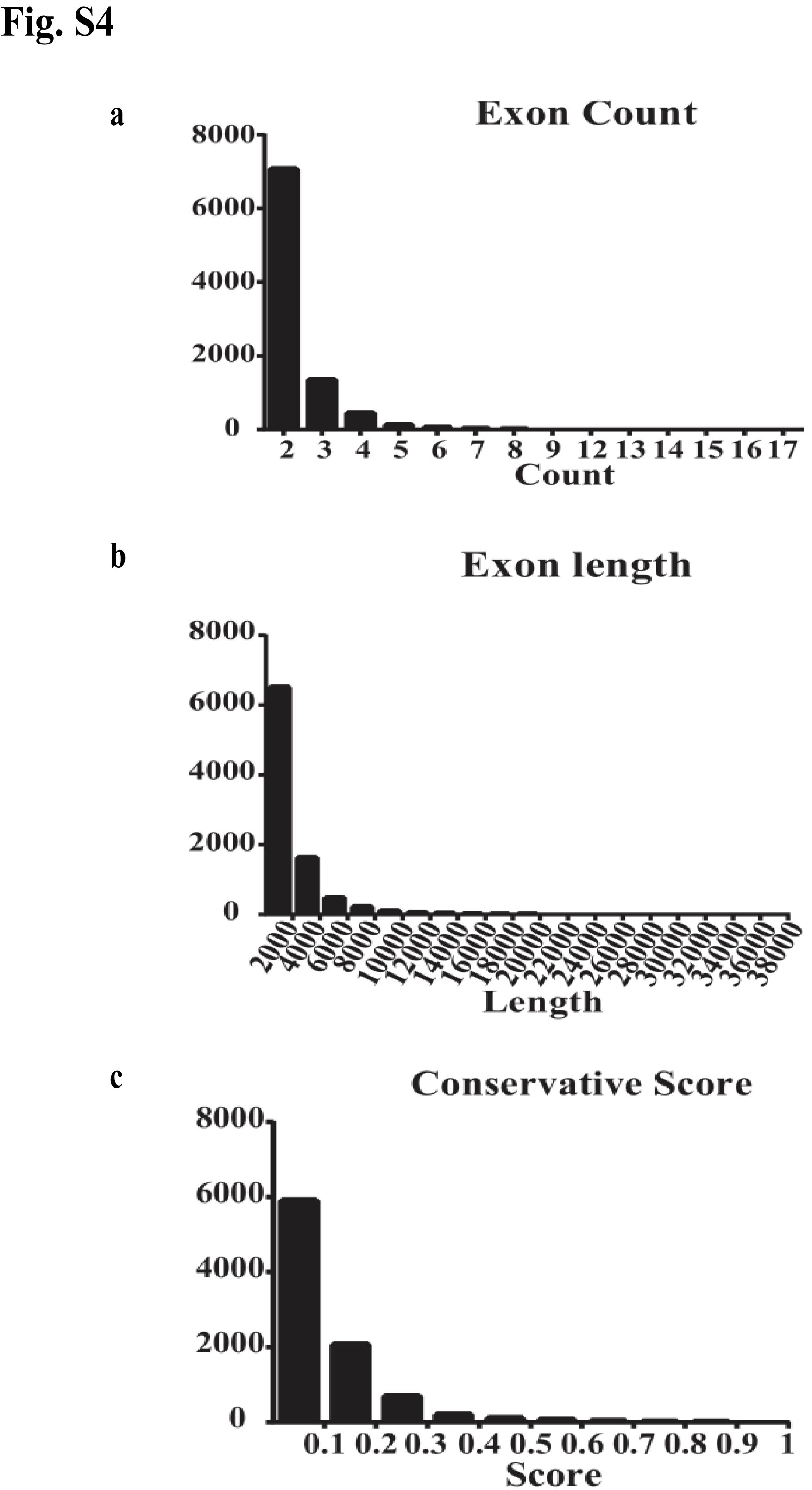

Supplement: Supplementary file 11 — Figure S4 [file 41420_2022_1019_MOESM11_ESM.png]

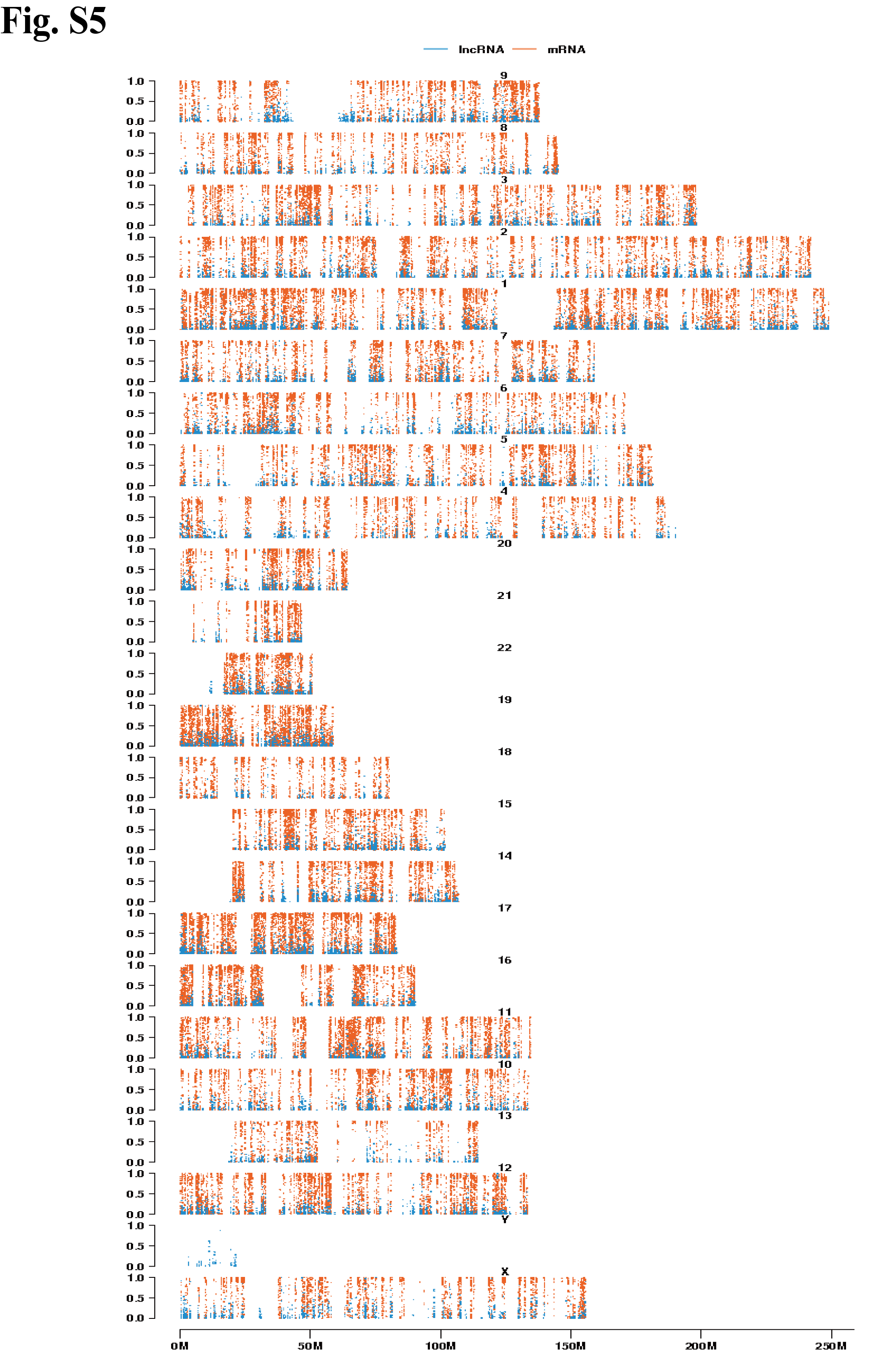

Supplement: Supplementary file 12 — Figure S5 [file 41420_2022_1019_MOESM12_ESM.png]

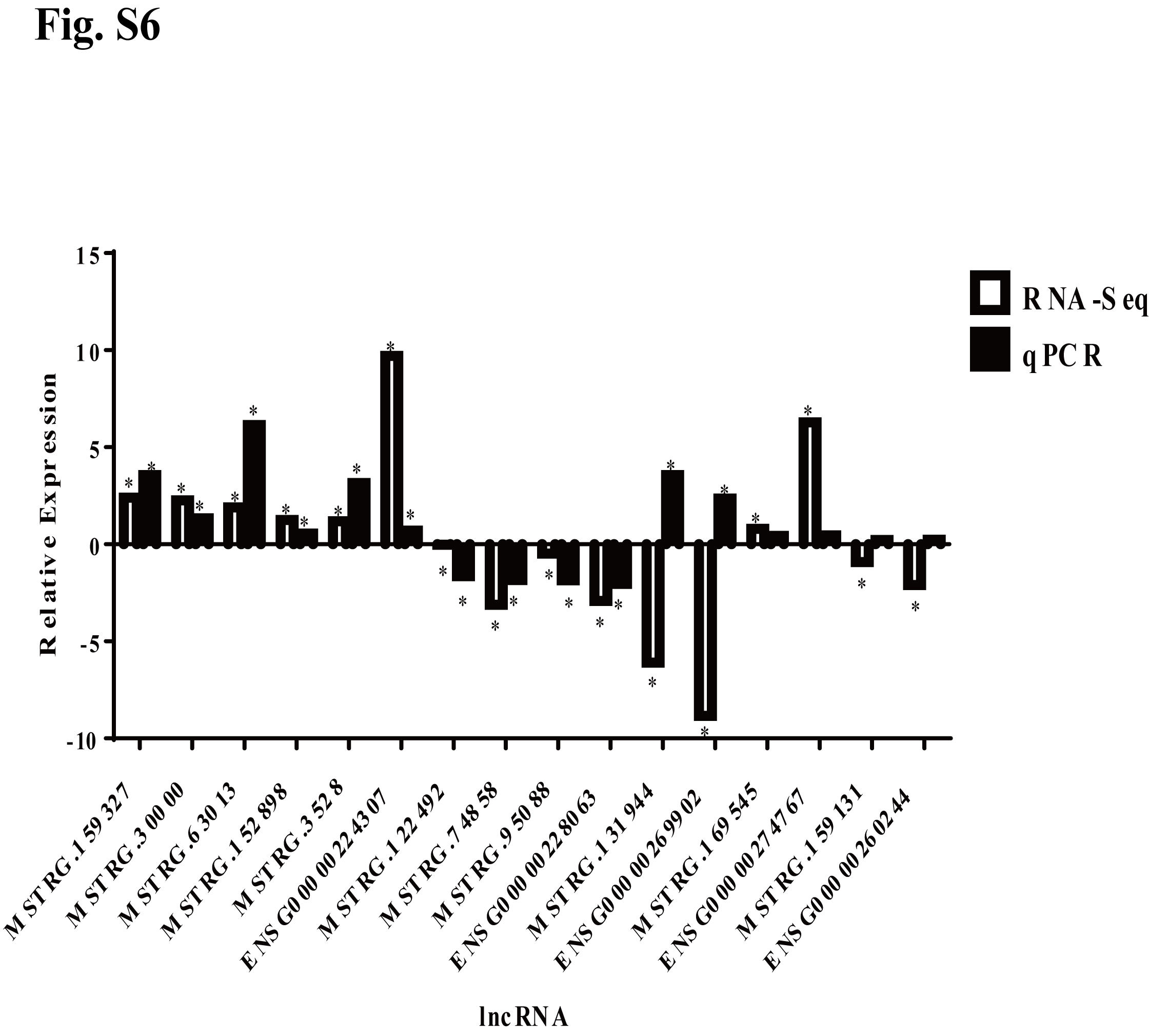

Supplement: Supplementary file 13 — Figure S6 [file 41420_2022_1019_MOESM13_ESM.png]

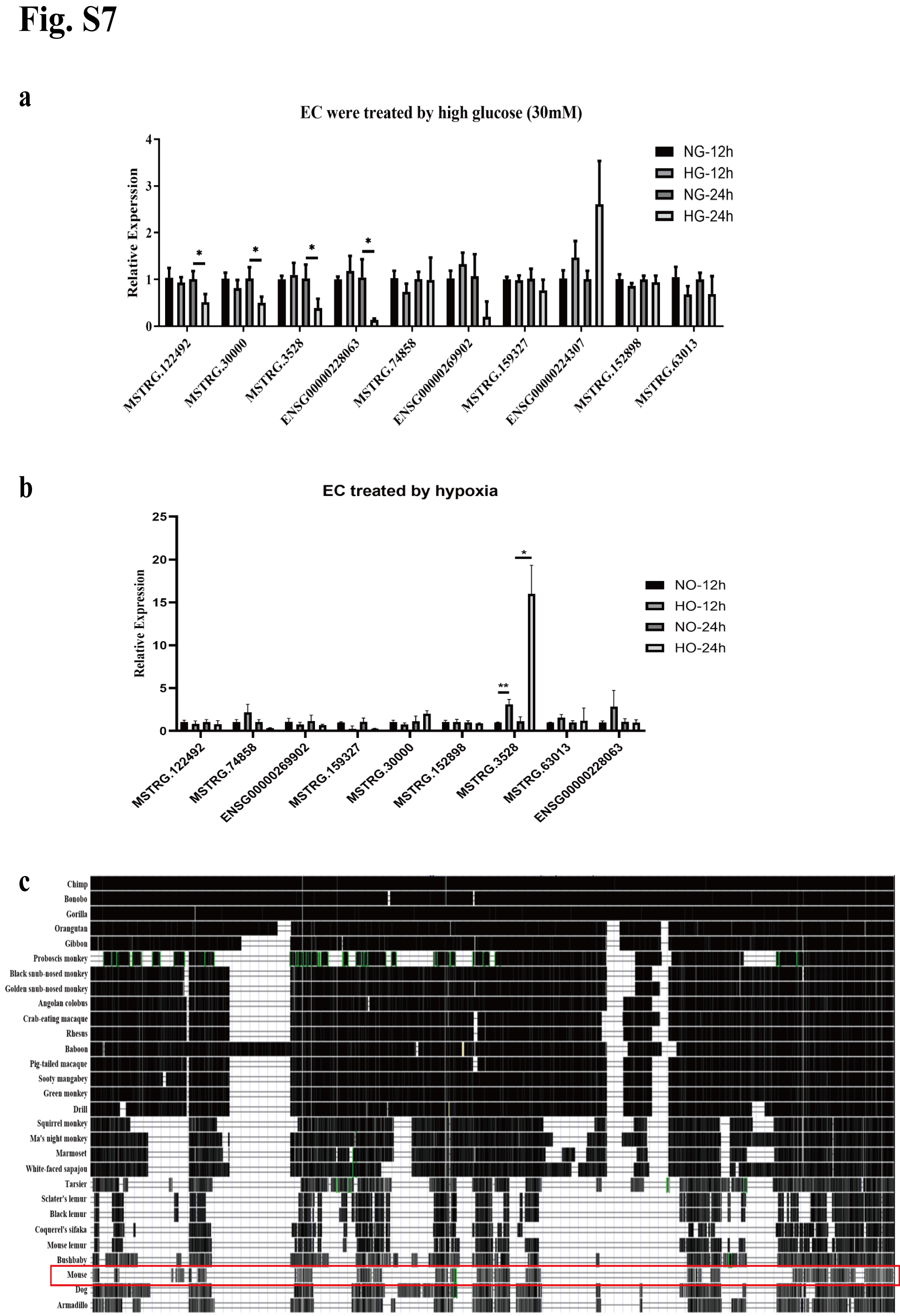

Supplement: Supplementary file 14 — Figure S7 [file 41420_2022_1019_MOESM14_ESM.png]

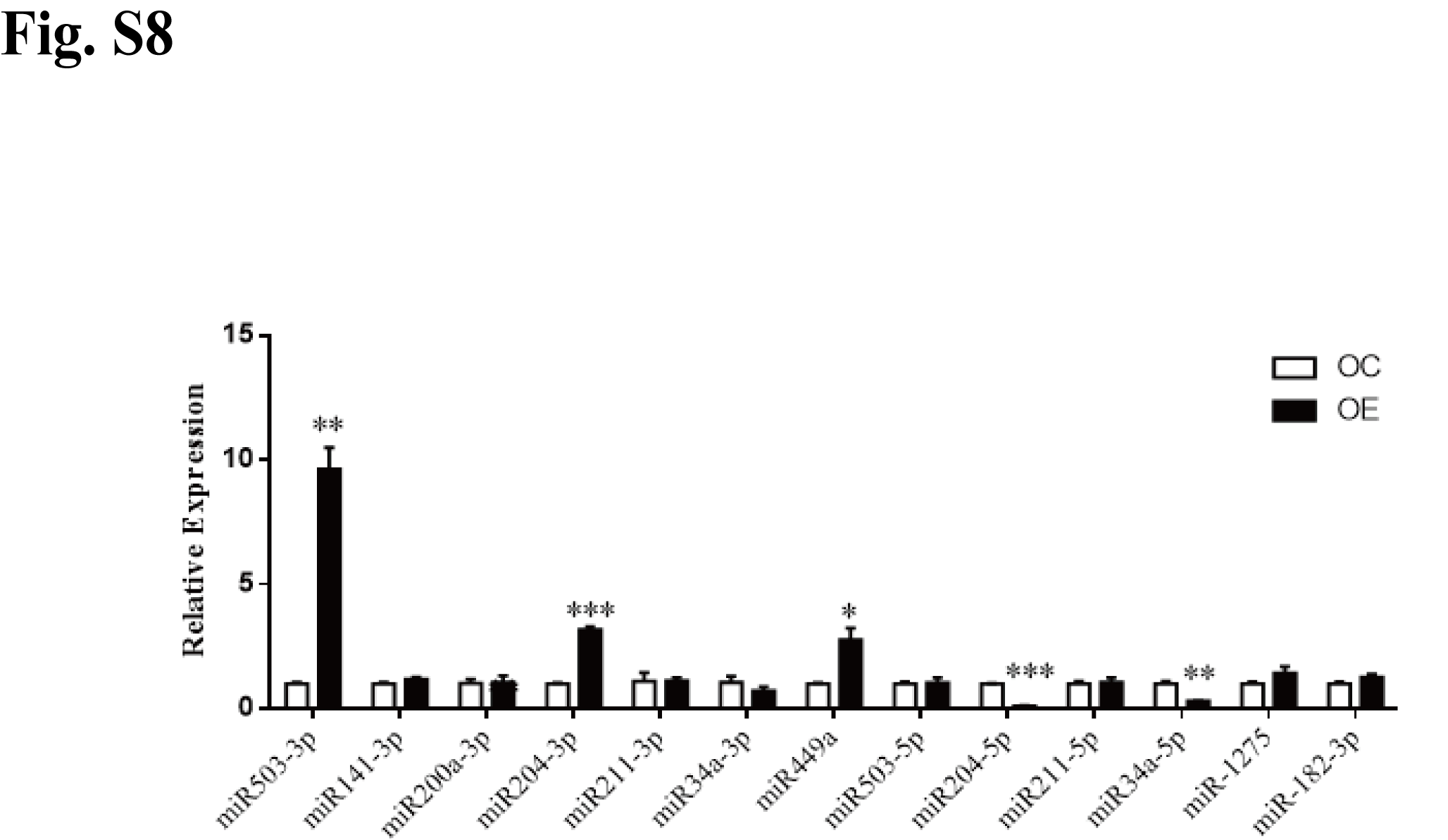

Supplement: Supplementary file 15 — Figure S8 [file 41420_2022_1019_MOESM15_ESM.png]

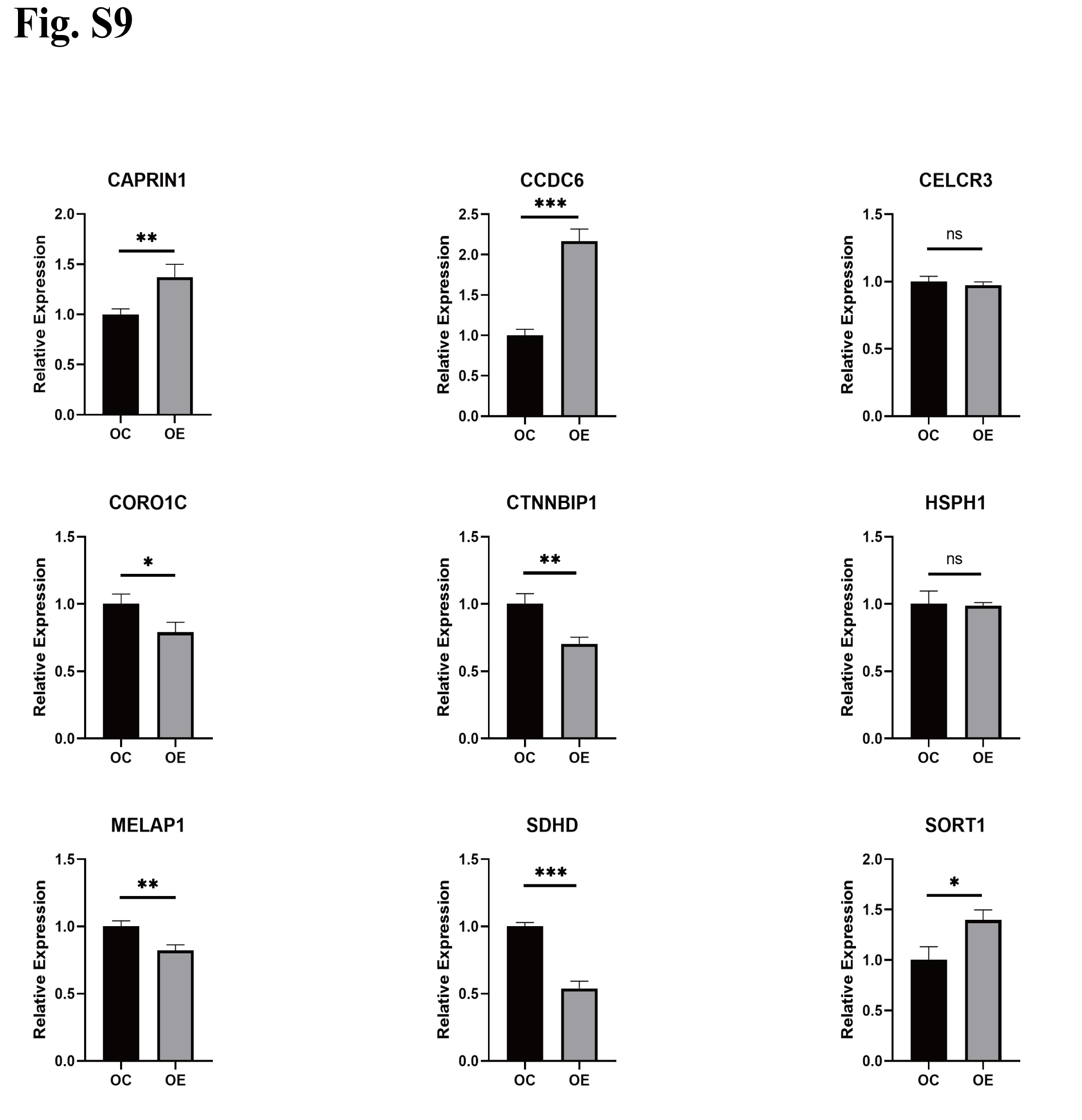

Supplement: Supplementary file 16 — Figure S9 [file 41420_2022_1019_MOESM16_ESM.png]

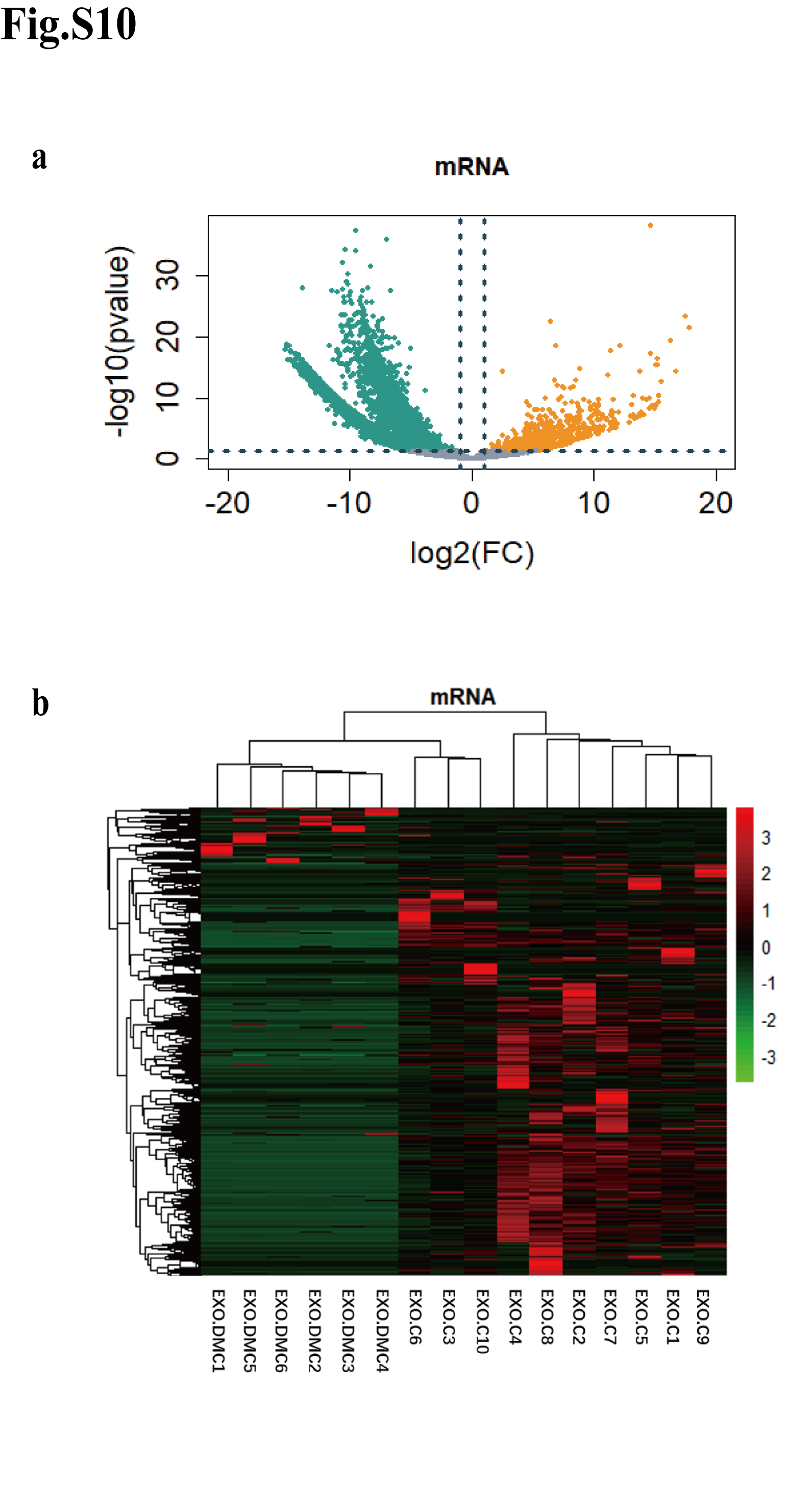

Supplement: Supplementary file 17 — Figure S10 [file 41420_2022_1019_MOESM17_ESM.png]

OC/OE SIRT1


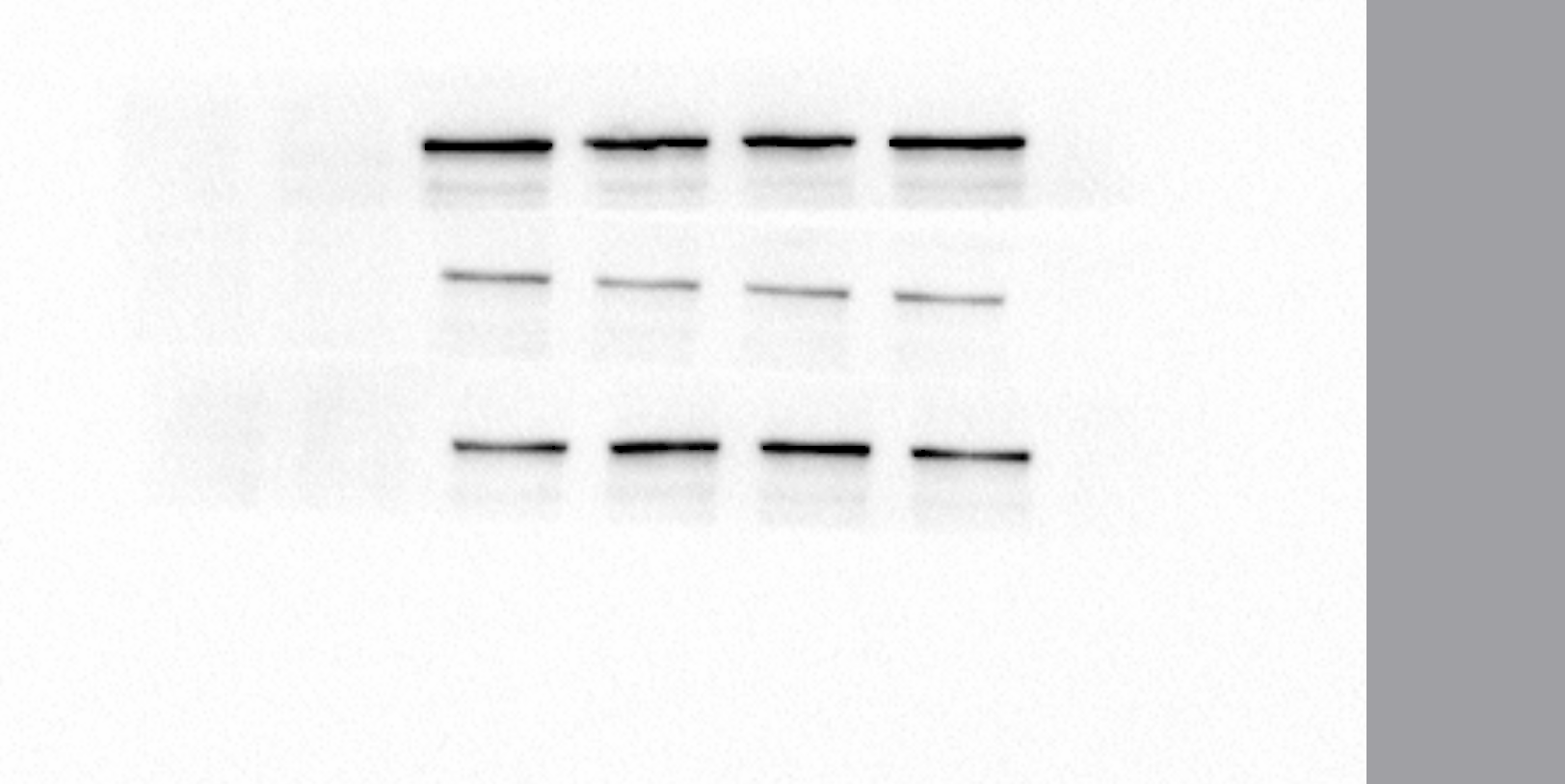


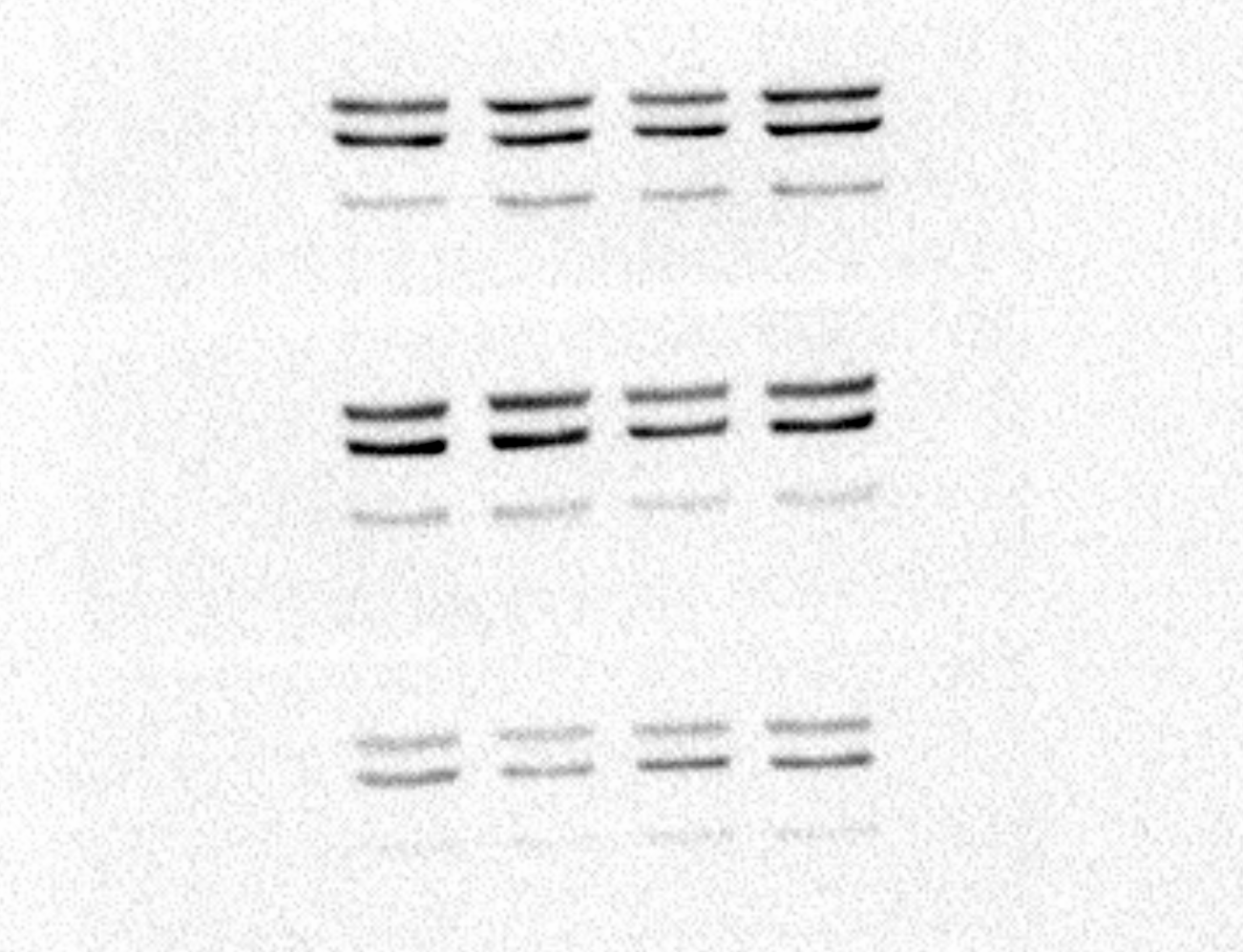


OE+NC/OE+204 SIRT1


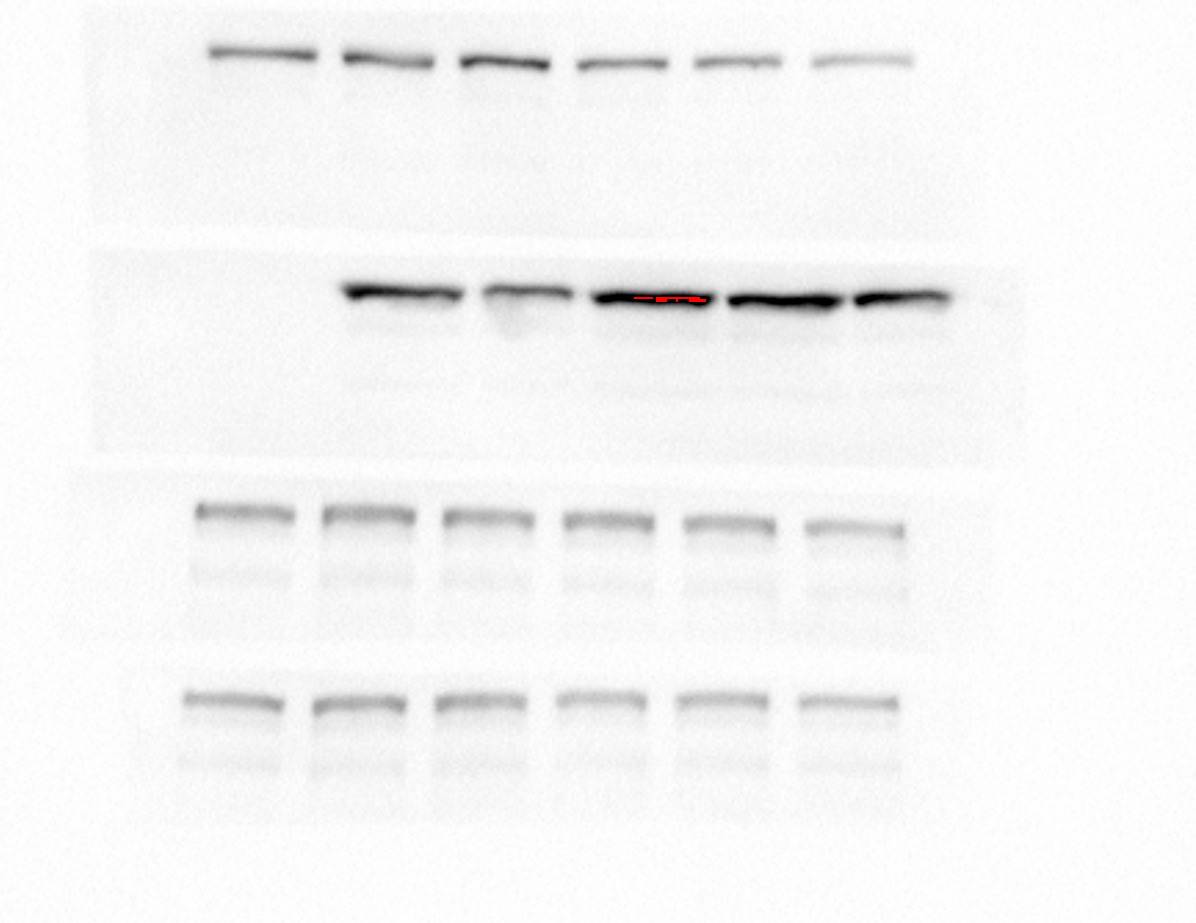


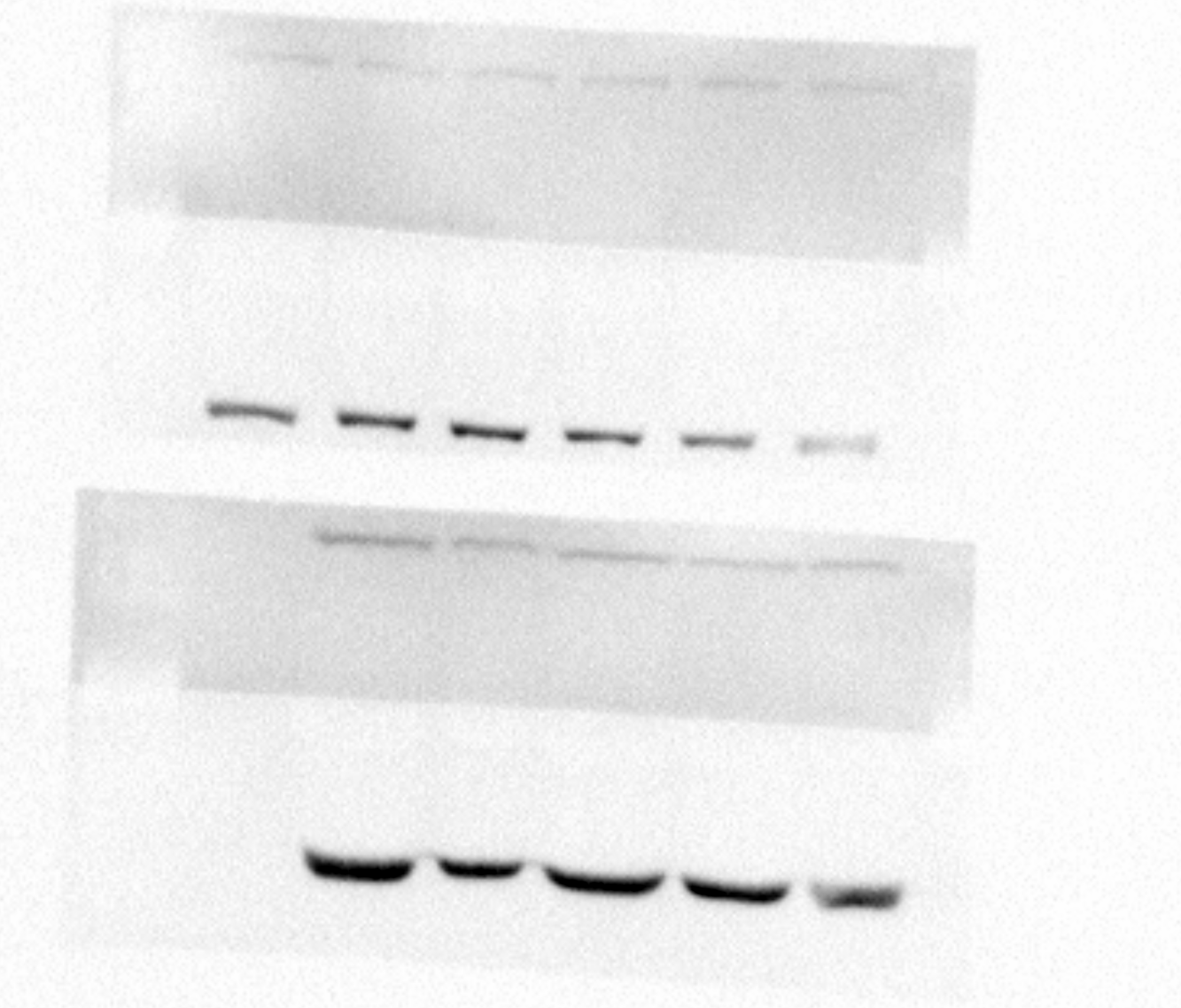


CD11b


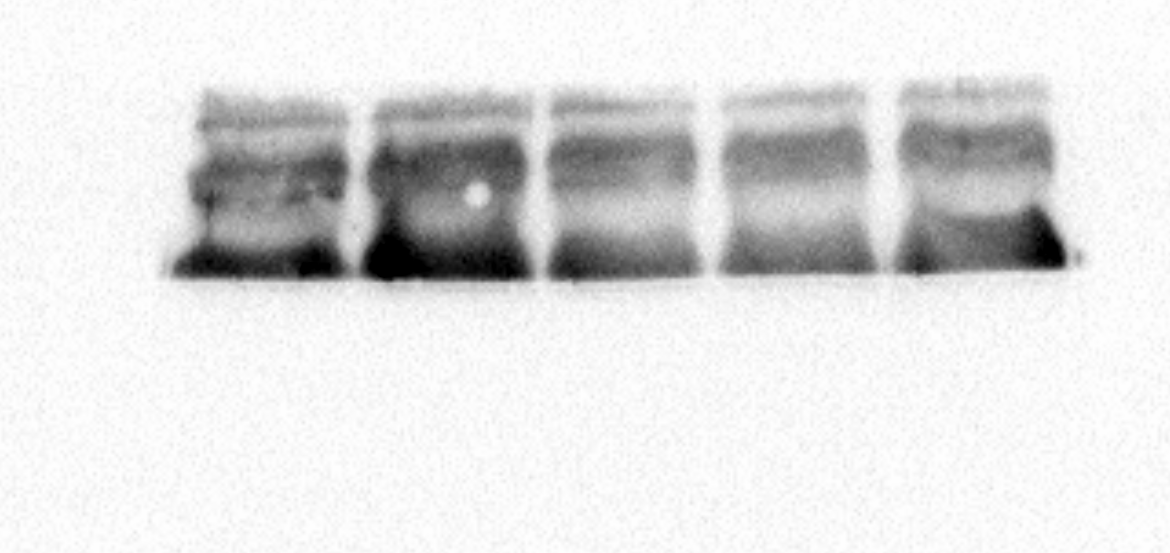

Supplement: Supplementary file 18 — WB [file 41420_2022_1019_MOESM18_ESM.docx]
