# Supplementary material for: LncRNA LYPLAL1-DT screening from type 2 diabetes with macrovascular complication contributes protective effects on human umbilical vein endothelial cells via regulating the miR-204-5p/SIRT1 axis
Source: Cell Death Discov. 2022 May 4;8:245. doi: 10.1038/s41420-022-01019-z (PMC9068612; doi:10.1038/s41420-022-01019-z)
Supplement: Supplementary file 19 — Cell Line STR Profile Report [file 41420_2022_1019_MOESM19_ESM.pdf]

Cell Line Authentication Service
STR Profile Report

GENETIC TESTING BIOTECHNOLOGY

**Sample Submitted By:**	Dr. Zuokang Zheng<br>Zhejiang Meisen Cell Technology Co.,Ltd.
**Email Address:**	947638289@qq.com
**Sales Order:**	201224B
**Cell Line Designation:**	HUVEC-T1
**Date Sample Received:**	Dec 24th, 2020
**Report Date:**	Jan 20th, 2021

Methodology: Nineteen short tandem repeat (STR) loci plus the gender determining locus, Amelogenin, were amplified using the commercially available EX20 Kit from AGCU. The cell line sample was processed using the ABI Prism® 3130 XL Genetic Analyzer. Data were analyzed using GeneMapper® ID v3.2 software (Applied Biosystems). Appropriate positive and negative controls were run and confirmed for each sample submitted.

Data Interpretation: Cell lines were authenticated using Short Tandem Repeat (STR) analysis as described in 2012 in ANSI Standard (ASN-0002) by the ATCC Standards Development Organization (SDO) and in Capes-Davis et al., Match criteria for human cell line authentication: Where do we draw the line? Int J Cancer. 2013;132(11):2510-9.

GTB™ performs STR Profiling following ISO 9001:2008 and ISO/IEC 17025:2005 quality standards.
There are no warranties with respect to the services or results supplied, express or implied, including, without limitation, any implied warranty of merchantability or fitness for a particular purpose. Genetic Testing Biotechnology (GTB) is not liable for any damages or injuries resulting from receipt and/or improper, inappropriate, negligent or other wrongful use of the test results supplied, and/or from misidentification, misrepresentation, or lack of accuracy of those results. Your exclusive remedy against GTB and those supplying materials used in the services for any losses or damage of any kind whatsoever, whether in contract, tort, or otherwise, shall be, at GTB's option, refund of the fee paid for such service or repeat of the service.

NOTE: According to the recommendations of *IJC* on cell line authentication, the report is valid for 3 years since the issue date.

Technical Questions?
GTB Technical Support
+86-512-67486171
service@jsdna.org
Section 505, Yixin BLD
SIP, Suzhou, 215123
Jiangsu, P.R. China

Ordering Questions?
order@jsdna.org
GTB Corporation
+86-512-62806339
Section 303, Yixin BLD
SIP, Suzhou, 215123
Jiangsu, P.R. China

GENETIC TESTING BIOTECHNOLOGY

Cell Line Authentication Service
STR Profile Report

Sales Order: 201224B

Loci	Test Results for Submitted Sample<br>Query Profile: HUVEC-T1		NICR Reference Database Profile<br>Database Profile: PUMC-HUVEC-T1	
Amelogenin	X		X	
D3S1358	15	17	15	17
D13S317	11	13	11	13
D7S820	8	11	8	11
D16S539	9	12	9	12
Penta E	15	17	15	17
TPOX	8	11	-	-
TH01	9		-	
D2S1338	17	19	17	19
CSF1PO	11	12	11	12
Penta D	10	12	-	-
D19S433	13		-	
vWA	14	16	14	16
D21S11	28	30	28	30
D18S51	16	17	16	17
D6S1043	11	14	11	14
D8S1179	13	14	13	14
D5S818	11	13	11	13
D12S391	20	21	-	-
FGA	24		24	

The allele match algorithm compares the 8 core loci plus amelogenin only, even though alleles from all loci will be reported when available.

Note: Loci highlighted in grey (8 core STR loci plus Amelogenin) can be made public to verify cell identity. In order to protect the identity of the donor, ***please do not publish*** *the allele calls from all the STR loci tested. The sample match is based on the reference data available at the time of comparison.*

Explanation of Test Results

Cell lines with ≥80% match are considered to be related; i.e., derived from a common ancestry. Cell lines with between a 55% to 80% match require further profiling for authentication of relatedness.

- ☐ The submitted sample profile is human, but not a match for any profile in the NICR STR database.
- ☑ The submitted profile is an exact match for the following human cell line(s) in the NICR STR database: PUMC-HUVEC-T1
- ☐ The submitted profile is similar to the following NICR human cell line(s):

e-Signature, Technician: Digitally signed by Xuekun Chen
DN: cn=Xuekun Chen, o=Genetic Testing Biotechnology (Suzhou), ou=DNA Typing Section, email=order@jsdna.org, c=CN
Date: 2021.01.20 16:48:11 +08'00'

GENETIC TESTING BIOTECHNOLOGY — GUARANTEE — CELL LINE AUTHENTICATION

e-Signature, Reviewer: Digitally signed by Xiankun Zhao
DN: cn=Xiankun Zhao, o=Genetic Testing Biotechnology (Suzhou), ou=Supervision Section, email=service@jsdna.org, c=CN
Date: 2021.01.20 16:48:40 +08'00'

Addendum: Electropherogram for the customer's sample set 1 of 1

GENETIC TESTING BIOTECHNOLOGY

Cell Line Authentication Service
STR Profile Report

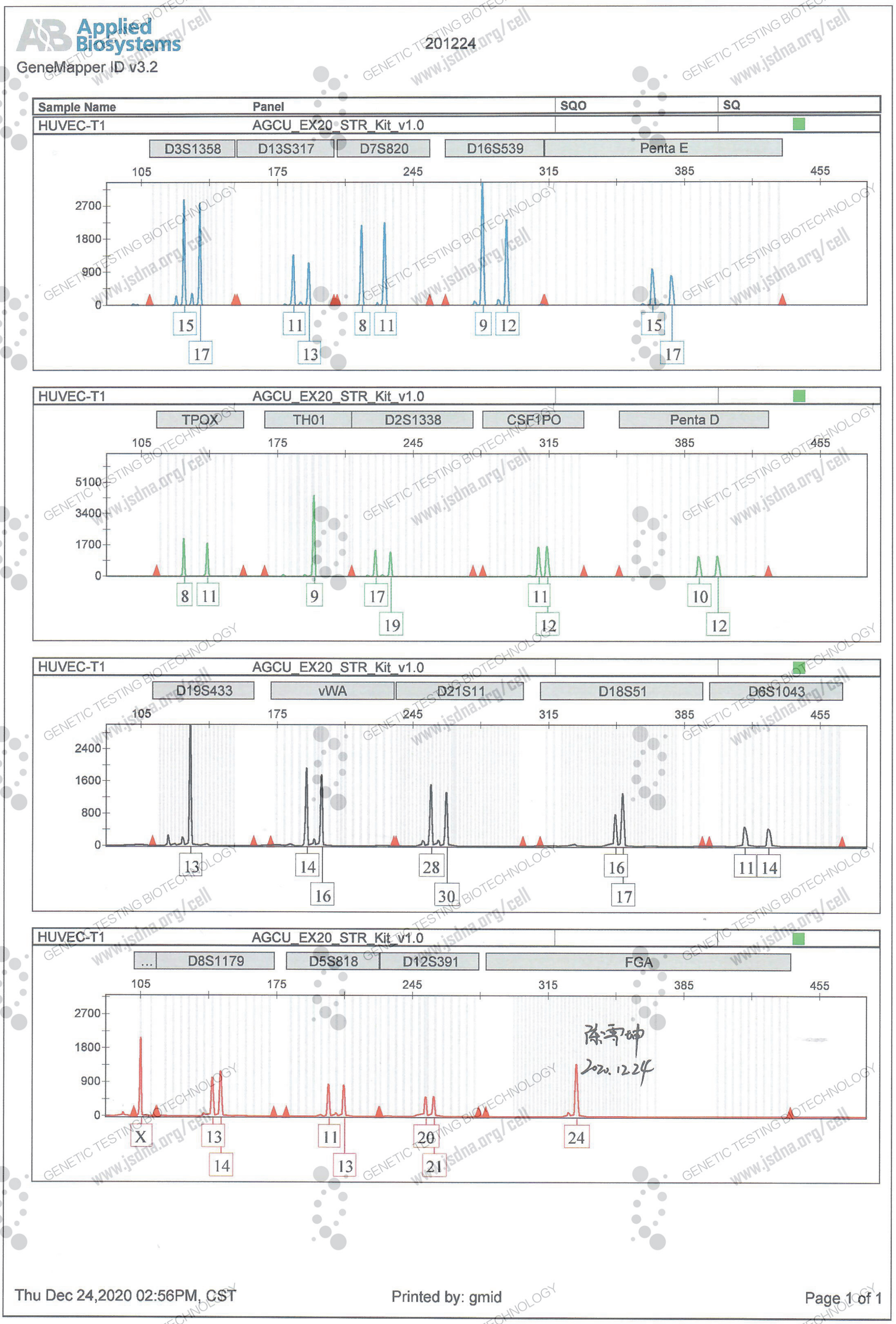

Cell Line Authentication Service
STR Profile Report

资源编号	3111C0001CCC000437
细胞名称	SV40T转化人脐静脉内皮细胞；PUMC-HUVEC-T1
形态特性	上皮样
生长特性	贴壁生长
特征特性	PUMC-HUVEC-T1形态呈现为典型的鹅卵石样排列，已证明在体外可传至40代。PUMC-HUVEC-T1内皮标记vWF、CD31、CD34均阳性，可结合凝[illegible]素，电镜下可见紧密连接和W-P小体，Matrigle上可形成管型。不同代数的HUVEC-T1细胞核型正常稳定（P12、P13、P19、P38），裸鼠体内接种不成瘤（P9和P33）。
培养条件	DMEM-H: Dulbecco ' s Modified Eagle ' s Medium (DME H-21 4.5g/Liter Glucose) 10%FBS + 1% NEAA
传代方法	1:3～1:4传代，一周1～2次
传代情况	P12
冻存条件	基础培养基+8%DMSO+20%FBS
支原体检测	培养法（-)
STR	Amelogenin：X；CSF1PO：11，12；D13S317：11，13；D16S539：9，12；D18S51：16，17；D21S11：28，30；D2S1338：17，19；D3S1358：15，17；D5S818：11，13；D6S1043：11，14；D7S820：8，11；D8S1179：13，14；FGA：24；Penta E：15，17；vWA：14，16；
同工酶	
染色体	46，XX
使用权限	A类
参考文献	
图片	PUMC——SV40T转化人脐静脉内皮细胞；PUMC-HUVEC-T1
